# Study protocol: optical coherence tomography angiography for the detection of neovascular age-related macular degeneration: a comprehensive multicentre diagnostic accuracy study in the UK—the ATHENA study

**DOI:** 10.1136/bmjopen-2022-070857

**Published:** 2024-05-31

**Authors:** Magdalena Niestrata, Jonathan J Deeks, Yemisi Takwoingi, Sobha Sivaprasad, Praveen J Patel, Pearse A Keane, Ashleigh Kernohan, Luke Vale, Alastair K Denniston, Richard Gale, Adam R Khan, William McKinnon, Ridhi Agarwal, Gabriella de Salvo, Evangelos Minos, Paulo Barbeiro, Usha Chakravarthy, Nadia K Waheed, Savita Madhusudhan, Tunde Peto, Konstantinos Balaskas

**Affiliations:** 1 NIHR Biomedical Research Centre, Moorfields Eye Hospital NHS Foundation Trust, London, UK; 2 Public Health, Epidemiology and Biostatistics, University of Birmingham, Birmingham, UK; 3 Public Health and Epidemiology, University of Birmingham, Birmingham, UK; 4 Newcastle University, Newcastle upon Tyne, UK; 5 University Hospitals Birmingham NHS Foundation Trust, Birmingham, UK; 6 York Teaching Hospital NHS Foundation Trust, York, UK; 7 University of Birmingham, Birmingham, UK; 8 Test Evaluation Research Group, Institute of Applied Health Research, University of Birmingham, Birmingham, UK; 9 Southampton University Hospitals NHS Trust, Southampton, UK; 10 Ophthalmology, North West Anglia NHS Foundation Trust, Peterborough, UK; 11 BlueWorks - United Kingdom, London, UK; 12 Ophthalmology, Queen's University Belfast, Belfast, UK; 13 New England Eye Center, Boston, Massachusetts, USA; 14 Liverpool University Hospitals NHS Foundation Trust, Liverpool, UK; 15 Faculty of Medicine Health and Life Sciences, Queen's University Belfast, Belfast, UK; 16 Institute of Ophthalmology, UCL, London, UK

**Keywords:** Ophthalmology, Medical retina

## Abstract

**Introduction:**

The diagnosis of neovascular age-related macular degeneration (nAMD), the leading cause of visual impairment in the developed world, relies on the interpretation of various imaging tests of the retina. These include invasive angiographic methods, such as Fundus Fluorescein Angiography (FFA) and, on occasion, Indocyanine-Green Angiography (ICGA). Newer, non-invasive imaging modalities, predominately Optical Coherence Tomography (OCT) and Optical Coherence Tomography Angiography (OCTA), have drastically transformed the diagnostic approach to nAMD. The aim of this study is to undertake a comprehensive diagnostic accuracy assessment of the various imaging modalities used in clinical practice for the diagnosis of nAMD (OCT, OCTA, FFA and, when a variant of nAMD called Polypoidal Choroidal Vasculopathy is suspected, ICGA) both alone and in various combinations.

**Methods and analysis:**

This is a non-inferiority, prospective, randomised diagnostic accuracy study of 1067 participants. Participants are patients with clinical features consistent with nAMD who present to a National Health Service secondary care ophthalmology unit in the UK. Patients will undergo OCT as per standard practice and those with suspicious features of nAMD on OCT will be approached for participation in the study. Patients who agree to take part will also undergo both OCTA and FFA (and ICGA if indicated). Interpretation of the imaging tests will be undertaken by clinicians at recruitment sites. A randomised design was selected to avoid bias from consecutive review of all imaging tests by the same clinician. The primary outcome of the study will be the difference in sensitivity and specificity between OCT+OCTA and OCT+FFA (±ICGA) for nAMD detection as interpreted by clinicians at recruitment sites.

**Ethics and dissemination:**

The study has been approved by the South Central—Oxford B Research Ethics Committee with reference number 21/SC/0412.

Dissemination of study results will involve peer-review publications, presentations at major national and international scientific conferences.

**Trial registration number:**

ISRCTN18313457.

**Strengths and limitations of this study**

- Large multicentre comprehensive diagnostic accuracy study in the UK of all imaging modalities in common clinical use for neovascular age-related macular degeneration (nAMD) diagnosis.
- The study design maximises its applicability to National Health Service clinical practice because test interpretation is made by clinicians at recruitment sites and it has an embedded economic evaluation.
- All imaging tests are transferred, randomly and after pseudonymisation, to a laboratory environment for unbiased review and determination of diagnostic accuracy of different imaging tests in isolation.
- The study design is complex because randomisation for sequence of image review is required to prevent bias from consecutive interpretation of all imaging tests for each case by the same clinician at recruitment sites.
- The eligibility criterion of presence of nAMD suspicious features on Optical Coherence Tomography (OCT) permits the assessment of positive predictive value only for OCT but this study design ensures the feasibility of the recruitment plan.

## Background and study aims

Age-related macular degeneration (AMD) is the most frequent cause of severe visual impairment and blindness in elderly populations in the developed world including the UK, Europe, the USA and Australia. With age being the strongest risk factor for the development of AMD, the numbers of affected patients are steadily increasing in view of the ageing population.^1^ Consequently, the incidence of visual impairment and blindness caused by AMD has significantly increased from 1990 to 2010.^2^ Worldwide, the number of patients affected by AMD is estimated to increase to 288 million in 2040.^3 4^

AMD is categorised into non-neovascular or dry and neovascular or wet, based on the absence or presence of macular neovascularisation (MNV).^5^ Neovascular AMD (nAMD) is characterised by fluid leakage or bleeding from new permeable vessels originating from the choroidal or deep retinal capillary network. MNV can be subclassified into type 1, otherwise known as occult, type 2 or classic and type 3 or retinal angiomatous proliferation, depending on the location of the MNV—beneath the retinal pigment epithelium, within the subretinal space and intraretinally, respectively.^6 7^

In nAMD regardless of MNV subtype, treatment with anti-vascular endothelial growth factor therapy has been shown to be effective. In those cases, early diagnosis and treatment are associated with improved visual prognosis and improved patient quality of life and reduced morbidity.^8^

The diagnosis relies on imaging techniques visualising the retina and choroidal circulation. Optical Coherence Tomography (OCT), which has revolutionised the diagnosis and management of numerous retinal pathologies, and features of nAMD disease activity including subretinal and intraretinal fluid, however, cannot directly detect a Choroidal Neovascularization (CNV). Until recently, Fundus Fluorescein Angiography (FFA) has been the gold standard for the diagnosis of nAMD as it allows for visualisation of leakage from MNV.^4^

Nonetheless, this procedure is associated with the risk of complications, such as discolouration of urine, nausea and vomiting, and carries an extremely low risk of anaphylactic reaction. It is also contraindicated in kidney failure, which is not uncommon in elderly patients with systemic comorbidities who are investigated for nAMD.^9^ The procedure is invasive, requiring intravenous cannulation and is unpleasant for patients. In addition, the acquisition of images from FFA is time-consuming and labour-consuming, leading to significant difficulty due to limited resources and increasing numbers of patients requiring diagnostics.^10^ Finally, occasionally FFA alone is not sufficient and additional imaging, such as Indocyanine-Green Angiography (ICGA) is required, particularly when polypoidal choroidal vasculopathy (PCV), a variant of nAMD, is suspected.

ICGA has long been considered as the standard for PCV diagnosis and is generally considered as essential to make a definitive diagnosis of PCV.^11^ Other imaging modalities have been assessed for the detection of PCV.^12^ Fukuyama *et al* evaluated 62 polypoidal lesions detected on ICGA and 79% were detected on OCTA.^13^ In a study which evaluated the Spectral-Domain Optical Coherence Tomography (SD-OCT) findings of 188 eyes with PCV or neovascular AMD, results suggested that SD-OCT had an 89% sensitivity and 85% specificity in identifying PCV when two of the three signs were present: Pigment Epithelium Detachment (PED), double-layer sign and thumb-like polyp.^14^

The advent of new non-invasive imaging techniques based on OCT, such as Optical Coherence Tomography Angiography (OCTA), has enabled a rapid direct visualisation of MNV without the need for intravenous contrast and without the side effects associated with FFA.

Faes and colleagues conducted a systematic review and meta-analysis of the performance of OCTA in detecting retinal disease. Over 1600 research articles involving OCTA have been published but only 17 studies were diagnostic accuracy studies. None of the studies were prospective and they provided scarce data on patient selection, data collection and analysis, masking, descriptions of the OCTA results and the reference standard. Out of the 17 studies, only five small studies evaluated the detection of nAMD. These reported a wide range of sensitivity (0.50–1.00) and specificity (0.68–1.00), reinforcing the need for a well-designed diagnostic accuracy study to inform National Health Service (NHS) decision-making on the role of OCTA.^15^

Cicinelli and colleagues have shown the overall sensitivity of OCTA for the detection of active CNV to be 80.7%.^16^ In addition, the OCTA images have been shown to be less affected by subretinal haemorrhage compared with FFA.^17 18^ In terms of PCV detection, Cheung and colleagues showed a combination of structural OCT and OCTA achieved 82.6% sensitivity and 100% specificity.^19^ As a result, RCOphth guidance and some countries, such as France, have already changed their guidelines advocating increasing use of OCTA instead of FFA for nAMD diagnosis.^20^ However, there are no prospective, statistically powered, diagnostic accuracy studies that could inform the optimal diagnostic imaging protocol for nAMD.

In addition to the clinical implications of the performance of OCTA compared with the other imaging strategies, there may be significant resource implications associated with the different strategies. As such, it is important to understand the relationship between any additional costs associated with certain imaging strategies and any benefits that they may provide. Alongside this diagnostic accuracy study, an economic decision modelling study will be used to assess the cost effectiveness of different strategies.

Due to the lack of high-quality evidence, there is variability in clinical practice with respect to the optimal imaging protocol for nAMD diagnosis. The original clinical interest in OCTA as a potential replacement for FFA in the diagnosis of nAMD has been superseded by the understanding that both techniques have relative and, on occasion, complementary merits, yet their optimal use for nAMD diagnosis remains unclear.^15^ The aim of ATHENA is to address these clinically relevant research questions.

There are also significant resource implications associated with the different strategies. Therefore, to guide decision- making the value for money of the different imaging strategies will be estimated.

## Methods

### Study design

The ATHENA study is a non-inferiority, prospective, randomised diagnostic accuracy study with embedded economic evaluation and an internal pilot to confirm the feasibility of the recruitment plan. The study started on 1 March 2022, with a planned end date of 31 December 2024.

### Patient and public involvement

There was extensive patient and public involvement, as detailed in the supplementary elements.

### Patient population

**Figure 1:**
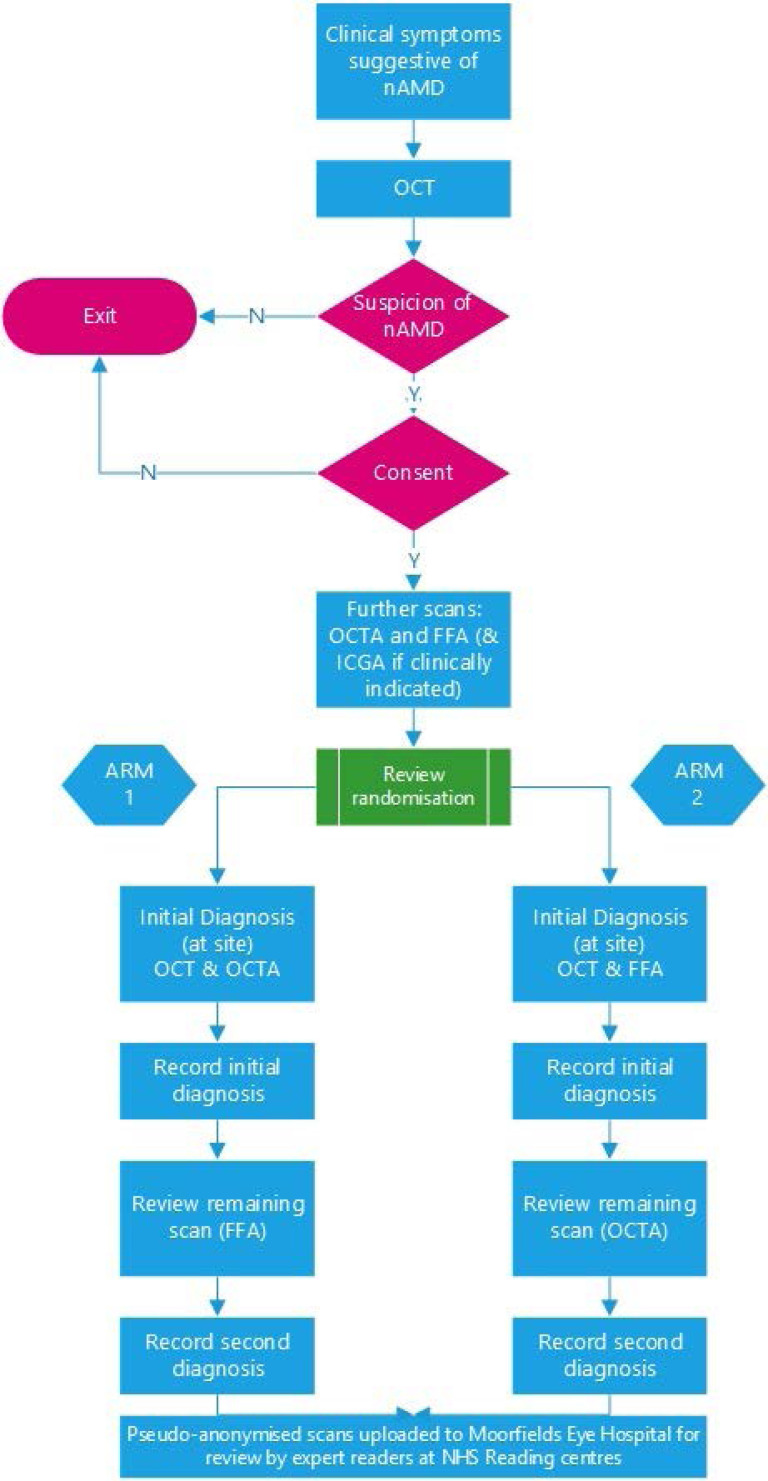
Study design.

Participants with a suspicion of nAMD in the first or second eye on OCT who present to an NHS secondary care ophthalmology unit in the UK will be recruited. They will be randomised to either OCT+FFA or OCT+OCTA study arms (figure 1).

### Study procedures

In addition to OCT, each patient will undergo both OCTA and FFA. Patients with clinical or OCT appearance suggestive of PCV will also undergo ICGA at the same time as FFA (figure 1).

In half the cases, selected randomly, an expert clinician at the recruitment site will review the OCT and then the OCTA and provide a clinical diagnosis (nAMD: yes/no). In the other half of the cases, the site clinician will review the OCT and then the FFA and provide a clinical diagnosis (nAMD: yes/no) (CD1) (figure 1). Subsequently, the same clinician will review the remaining imaging test in each case (FFA or OCTA) and will either confirm or change their initial clinical diagnosis (nAMD: yes/no)—recorded as clinical diagnosis 2 (CD2) (figure 1). Comparison of the two clinical diagnoses within each arm will provide additional evidence concerning the added value of using a combination of both OCTA and FFA in patients with an OCT suggestive of nAMD.

### Setting

Recruitment will take place in 20 hospital-based eye units across the UK. Of those six will be involved in the pilot study and all sites will be involved in the main study. The study coordinating centre (Trial Office) is the Birmingham Clinical Trials Unit (BCTU), based at the University of Birmingham.

Participants will be recruited among patients referred by their community optometrist, General Practitioner (GP), or self-referred to secondary care eye casualty departments with a suspicion of nAMD and among patients already under the care of NHS eye units for an established diagnosis of nAMD in one eye who develop symptoms of it in the fellow eye. Using eligibility criteria to define inclusion and exclusion criteria for potential patients (table 1).

**Table 1:** Eligibility criteria

Inclusion criteria	Exclusion criteria
Patients with suspicion of nAMD in the first or second eye who have	Significant media opacities (cataract, vitreous opacities) that would not allow good quality fundus imaging
Signed informed consent form	Diabetic retinopathy of severity worse than mild non-proliferative stage and with any degree of diabetic maculopathy
Ability to perform study-specific procedures	Other causes of choroidal neovascularisation (myopic, angioid streaks, inflammatory, retinal dystrophies, secondary to central serous chorioretinopathy, idiopathic)
	Inability to undergo dye-based imaging (FFA or ICGA) due to history of allergy

FFA, Fundus Fluorescein Angiography; ICGA, Indocyanine-Green Angiogrpahy; nAMD, neovascular age-related macular degeneration.

### Randomisation and blinding

The purpose of randomisation in this study is to minimise bias in the comparative accuracy of OCT+OCTA and OCT+FFA (primary objective) that may arise from non-masked interpretation of the findings of OCTA and FFA by the same clinician. Randomisation will be provided by a secure online randomisation system at BCTU (http://www.trials.bham.ac.uk/athena), which will be available 24 hours a day, 7 days a week, apart from short periods of scheduled maintenance. The system will randomise each patient to one of the two study arms as described in the study design (figure 1).

A minimisation algorithm will be used within the online randomisation system to ensure balance in the treatment allocation over the following variables: the recruiting centre, whether the affected eye is the first or second eye and whether the case is suspicious of PCV. A ‘random element’ will be included in the minimisation algorithm, so that each patient has a probability of being randomised to the opposite study arm than they would otherwise be assigned to.

### Sample size calculation

According to current literature, the sensitivity of OCT+OCTA and OCT+FFA for the detection of nAMD is 92%^15 21 22^ and 90%,^23^ respectively. Based on the above, 240 patients with nAMD will need to be recruited to ensure, with 90% certainty, that the upper limit of a one-sided 97.5% CI (or equivalently a 95% two-sided CI) will exclude a difference in favour of OCT+FFA of more than 10 percentage points (ie, the non-inferiority margin). A non-inferiority margin of 10 percentage points was deemed appropriate in view of the expected advantages of OCTA (ie, improved patient experience through the use of a quick non-invasive imaging technique) and an already established role in clinical practice.

Audit data from Moorfields Eye Hospital indicated a prevalence of 75% of confirmed nAMD in patients with a positive or suspicious OCT, based on which, 320 patients with suspected nAMD will be required. However, as the aim of the study is to compare specificity as well as sensitivity, using the same estimates as for sensitivity, it is anticipated that 960 patients will be required. Allowing for 10% withdrawal rate, missing data and inconclusive test results, we would aim to recruit a total of 1067 patients.

### Objectives—outcome measures and their analysis

The primary analysis, against the objectives (table 2), will be based on a pragmatic approach to image interpretation for nAMD detection by clinicians at recruitment sites (comparison of OCT+OCTA vs OCT+FFA in patients with positive or suspicious OCT). This approach reflects real-life clinical practice and will allow a meaningful assessment of the diagnostic accuracy of the most commonly used confirmatory tests for nAMD detection in a clinical setting. This approach will enhance the potential for generalisability of study results in the NHS.

**Table 2:** Objectives

Primary objective	Primary outcome measure
The main aim of this study is to assess whether the sensitivity and specificity of OCTA combined with OCT is non-inferior to that of FFA combined with OCT, for the detection of neovascular age-related macular degeneration, in patients with clinical and OCT features suggestive of the diagnosis; as interpreted by clinicians.	The primary outcome measure is the difference in sensitivity and specificity between OCTA combined with OCT and FFA combined with OCT. The unit of analysis is the patient (one ‘trial’ eye per patient). The sensitivity, specificity, PPV and NPV of the tests will be calculated. The diagnostic accuracy of OCT+OCTA and OCT+FFA will be compared with assess non-inferiority. The differences in sensitivity and specificity will be established and two-sided 95% CIs calculated using the Wilson CI score method. If the lower boundary of the two-sided 95% CI for the difference in sensitivity and specificity of OCT+OCTA relative to OCT+FFA is higher than −10 percentage points, the sensitivity of OCT+OCTA will be considered as non-inferior.
**Secondary objectives**	**Secondary outcome measures**
To assess the diagnostic accuracy of OCTA alone and FFA alone as reviewed by Reading Centre expert graders for the detection of nAMD in patients with a positive or suspicious OCT.	Sensitivity, specificity, PPV and NPV of OCTA and FFA in isolation will be assessed on the basis of masked review by expert graders in the Reading Centres; 95% CIs will be calculated using the Wilson method.
To assess the positive predictive value (PPV) of OCT for the detection of nAMD in all patients presenting with suspicion of nAMD.	The PPV of OCT for nAMD detection and its 95% CI will be estimated. Since only patients with a positive or suspicious OCT will be included, the sensitivity, specificity and NPV of OCT alone cannot be assessed.
To compare the diagnostic accuracy of the combination of OCT+FFA vs OCT+FFA+ OCTA as interpreted by retinal experts within the ‘OCT+FFA’ arm of the study.	In order to establish the added diagnostic value of using a third imaging technique for nAMD detection, OCT+FFA will be compared against OCT+FFA+ OCTA, in terms of differences in sensitivity and specificity together with their 95% CIs.
To compare the diagnostic accuracy of the combination of OCT+OCTA vs OCT+FFA+ OCTA as interpreted by retinal experts for the detection of nAMD within the ‘OCT+OCTA’ arm of the study.	In order to establish the added diagnostic value of using a third imaging technique for nAMD detection, OCT+OCTA will be compared against OCT+OCTA+ FFA, in terms of differences in sensitivity and specificity together with their 95% CIs.
For a subset of cases with OCT and clinical features suspicious of Polypoidal Choroidal Vasculopathy (PCV) that underwent Indocyanine-Green Angiography (ICGA), to assess diagnostic accuracy of OCTA, FFA, ICGA, alone and in combinations, for the detection of PCV.	For a subset of cases with clinical and OCT features suspicious of PCV, the diagnostic accuracy of OCTA, FFA and ICGA alone and in combination will be assessed by calculating their sensitivity, specificity, PPV and NPV (including their 95% CIs).
To assess intra-rater and inter-rater agreement in the detection of nAMD on OCTA and FFA as assessed by Reading Centre graders.	For OCTA and FFA, intra-rater and inter-rater agreement will be determined using percentage agreement and Gwet’s first-order agreement coefficient (Gwet’s AC1).^25^
To develop and validate criteria for the OCTA-based diagnosis of nAMD.	The framework for OCTA-based nAMD diagnostic criteria created through this study will be assessed against a reference standard from the Reading Centres using all available multimodal imaging (OCT, OCTA, FFA and ICGA where available) enhanced with clinical vignette describing patient symptoms and follow-up clinical data.
To estimate the incremental cost per true positive detected and incremental cost per correct diagnosis for nAMD through a model-based cost-effectiveness analysis.	A model-based cost-effectiveness analyses (CEA) will be conducted to estimate the incremental cost per true positive detected and incremental cost per correct diagnosis for nAMD.
To assess the diagnostic accuracy of OCTA by lesion type (PCV, type 1-, type 2-, type 3-nAMD).	Sensitivity, specificity, PPV and NPV (including their 95% CIs) of OCTA for detection on nAMD by lesion type (PCV, type 1-, type 2-, type 3-nAMD) will be computed based on assessment by expert graders in the Reading Centres.
To report limitations of OCTA use and adverse events.	Number and classification of adverse events.

FFA, Fundus Fluorescein Angiography; nAMD, neovascular age-related macular degeneration; NPV, negative predictive value; OCT, Optical Coherence Tomography; OCTA, Optical Coherence Tomography Angiography.

**Figure 2:**
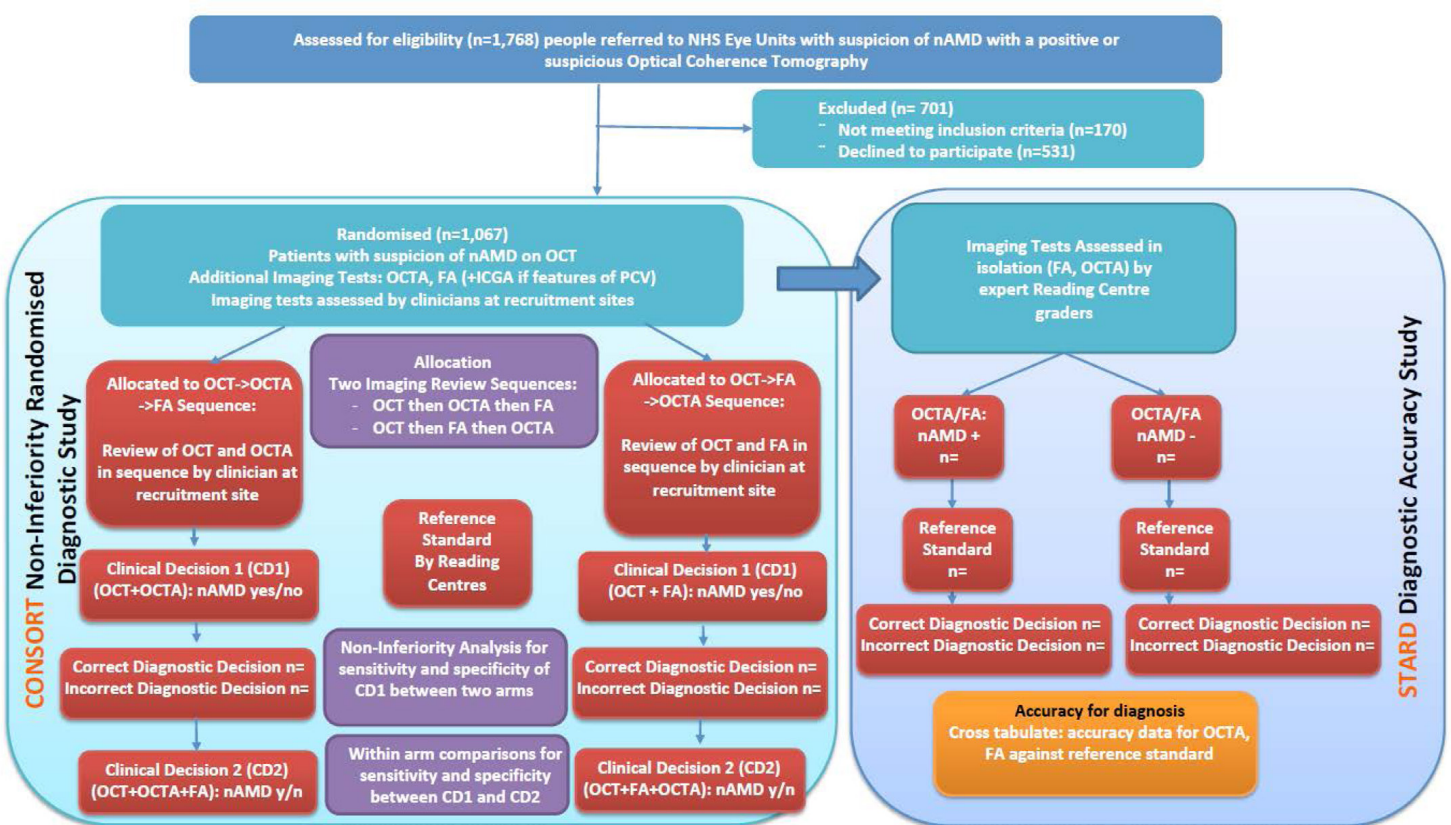
Methodological flowchart.

All images (OCT, OCTA, FA and where available ICGA) will also be transferred to NetwORC UK Reading Centres Network using a secure web-based image management system. The accuracy of the individual imaging tests in isolation (OCT, OCTA, FA) will be assessed in a masked and random fashion by expert graders in the controlled environment of the Reading Centres. This will ensure the masked assessment of individual imaging tests independent of each other with the use of a sophisticated grading queue that ensures random allocation of tests and minimises bias. Grading of the images will be performed at the three Reading Centres by trained graders who have achieved a minimum of 90% agreement in the accreditation test, as per figure 2 which highlights the unique methodology that combines an RCT (CONSORT diagram) with a Diagnostic Accuracy study (STARD diagram). The allocation of images will take into account the study design so that graders involved in grading of the reference standard will not be involved in grading the index tests. Dedicated image management software will be used for the purposes of aggregating imaging data from recruitment sites and developing grading queues for Reading Centre graders. The imaging tests will be presented in random order to the graders to avoid bias associated with grading images from the same patient in sequence. Graders will be masked to all clinical information as well as whether the imaging tests being reviewed originate from the same patient or not.

### Reference standard

To provide the reference standard we will use all available imaging information from multimodal imaging (OCT, OCTA, FFA and ICGA where available) enhanced with clinical vignette describing patient symptoms and available follow-up clinical data. The images will be interpreted by Reading Centre graders different to those involved in the masked assessment of the individual imaging tests and they will review all imaging and clinical information for each patient. Uncertain cases by primary grading will be reviewed by a senior adjudication panel (directors of the three Reading Centres) who will provide the reference standard in these cases.

Two versions of the reference standard will be provided:

- RS1 based on all imaging modalities except OCTA.
- RS2 based on all imaging modalities including OCTA.

This will allow assessment of the potential effect of incorporation bias from inclusion of the primary index test (OCTA) in determining the reference standard. The primary analyses will be based on RS1.

### Economic evaluation

In addition to the clinical diagnostic accuracy study, a model-based cost effectiveness analysis will be carried out to estimate the costs and benefits of each approach. The model will compare different strategies for tests (ie, different combinations of tests, different rules for diagnosis or subsequent testing). The time horizon for the model will cover the diagnostic testing period only and the evaluation perspective will be from the NHS perspective. The model structure will be finalised throughout the course of the study but will likely take the form of a decision tree. The model results will be expressed an incremental cost effectiveness ratio. Specifically, this will be presented as a cost per true positive detected for each of the strategies.

Both deterministic sensitivity analysis (DSA) and probabilistic sensitivity analysis (PSA) will be carried out. The DSA will assess plausible variation for parameters (eg, differences in number of resources or unit costs) and how this impacts conclusions. The PSA will assess joint impression around the parameters and how this impacts the model estimates. This will likely take the form of a Monte Carlo simulation to address uncertainty. This model will adhere to best practices for economic decisions.^24^

### Data management

Processes will be employed to ensure the accuracy of data included in the final report. Coding and validation will be agreed on by the study manager, statistician and programmer; and the study database will be signed off once the implementation of these has been assured.

Data will be entered onto the study database by staff involved in the study or delegated staff at sites. Data entries within the electronic database will be scrutinised for completeness and accuracy, with discrepancies checked against paper data forms. The data capture system will conduct automatic range checks for specific data values to ensure high levels of data quality. Any queries raised will be registered using the integrated data query system in the study database, with the expectation to be resolved within 30 days.

Every attempt will be made to collect full imaging data on all study participants, and the proportion of missing data is expected to be minimal. Participants with indeterminate or missing results will not be included in the primary analysis.

### Study termination

The end of study will be 6 months after the last data capture. This will allow sufficient time for the completion of protocol procedures, data collection and data input. The Trials Office will notify the Research Ethics Committee (REC) that the study has ended within 90 days of completion. In the instance of terminating the study early, the Trials Office will inform the REC within 15 days of study termination. The REC and Trial Office will receive a summary of the clinical study report within 12 months of study completion.

### Consent and ethics

The study will be performed in accordance with the recommendations guiding physicians in biomedical research involving human subjects, adopted by the 18th World Medical Association General Assembly, Helsinki, Finland, 1964, amended by the 48th WMA General Assembly, Somerset West, Republic of South Africa, 1996

Favourable opinion has been provided by the Research Ethics Committee and the study has received approval by the UK Health Research Authority.

Study participants will be required to give informed consent and sign a consent form. Trial participation is entirely voluntary, and the consent may be withdrawn at any time without this affecting patients’ care.

### Dissemination

#### Study dataset dissemination

The final pseudonymised study dataset will be made available to members of the study management group so that publications describing the outcomes (table 2) can be prepared.

Following publication of findings, the fully anonymised study dataset will be made available to external researchers on approval from the study management group, the study steering committee, and the sponsor in line with best data sharing practices for clinical study datasets.

#### Dissemination of results

Results of this study will be submitted for publication in high impact peer reviewed journals.

The study question is important and has the potential to change clinical practice. Should the study results support this, the change in practice is expected to be rapidly incorporated into

guidelines including the National Institute of Health and Care Excellence and the Scientific Advisory Committees in the Department of Health.

The findings will be presented and disseminated via national and international conferences. A copy of the monograph will be lodged with the NIHR Journals Library as a permanent and comprehensive record of the study. In consultation with the investigators and relevant journal, a press release will be issued to the media on publication of the results.

Results of the study will be shared with study participants, staff members at research sites and investigators of other studies related to any of the imaging techniques investigated in ATHENA. Outreach to other key stakeholders (study networks, health advocates) involved in related studies is planned.

## Supplementary Material

Reviewer comments

Author's manuscript
