# Supplementary material for: Study protocol: optical coherence tomography angiography for the detection of neovascular age-related macular degeneration: a comprehensive multicentre diagnostic accuracy study in the UK—the ATHENA study
Source: BMJ Open. 2024 May 31;14(5):e070857. doi: 10.1136/bmjopen-2022-070857 (PMC11149127; doi:10.1136/bmjopen-2022-070857)
Supplement: Author's manuscript [file bmjopen-2022-070857.draft_revisions.pdf]

BMJ Open is committed to open peer review. As part of this commitment we make the peer review history of every article we publish publicly available.

When an article is published we post the peer reviewers' comments and the authors' responses online. We also post the versions of the paper that were used during peer review. These are the versions that the peer review comments apply to.

The versions of the paper that follow are the versions that were submitted during the peer review process. They are not the versions of record or the final published versions. They should not be cited or distributed as the published version of this manuscript.

BMJ Open is an open access journal and the full, final, typeset and author-corrected version of record of the manuscript is available on our site with no access controls, subscription charges or pay-per-view fees (http://bmjopen.bmj.com).

If you have any questions on BMJ Open's open peer review process please email info.bmjopen@bmj.com

BMJ Open

Optical Coherence Tomography Angiography for the detection of Neovascular Age-related Macular Degeneration: a Comprehensive Diagnostic Accuracy Study - the ATHENA study

Journal:	*BMJ Open*
Manuscript ID	bmjopen-2022-070857
Article Type:	Protocol
Date Submitted by the Author:	30-Dec-2022

Complete List of Authors:	Niestrata, Magdalena; Moorfields Eye Hospital City Road Campus, Ophthalmology<br>Deeks, Jonathan; University of Birmingham, Public Health, Epidemiology and Biostatistics<br>Takwoingi, Yemisi; University of Birmingham, Public Health and Epidemiology<br>Sivaprasad, Sobha; Moorfields Eye Hospital City Road Campus<br>Patel, Praveen; Moorfields Eye Hospital NHS Foundation Trust, NIHR Biomedical Research Centre<br>Keane, Pearse; Moorfields Eye Hospital NHS Foundation Trust<br>Kernohan, Ashleigh; Newcastle University<br>Vale, Luke; Newcastle University<br>Denniston, Alastair; Queen Elizabeth Hospital Birmingham, UK<br>Gale, Richard; York Teaching Hospital NHS Foundation Trust<br>Khan, Adam R.; University of Birmingham<br>McKinnon, William; University of Birmingham<br>Agarwal, Ridhi; University of Birmingham, Test Evaluation Research Group, Institute of Applied Health Research,<br>de Salvo, Gabriella; Southampton University Hospitals NHS Trust<br>Minos, Evangelos; North West Anglia NHS Foundation Trust, Ophthalmology<br>Barbeiro, Paulo; BlueWorks - United Kingdom<br>Chakravarthy, Usha; Queen's University Belfast, Ophthalmology<br>Waheed, Nadia K; New England Eye Center<br>Madhusudhan, Savita; Liverpool University Hospitals NHS Foundation Trust<br>Peto, Tunde; Queen's University Belfast Faculty of Medicine Health and Life Sciences<br>Balaskas, Konstantinos; NIHR Moorfields Biomedical Research Centre, NHS Foundation Trust and UCL Institute of Ophthalmology

Keywords:	Ophthalmology < SURGERY, OPHTHALMOLOGY, Medical retina < OPHTHALMOLOGY

SCHOLARONE™
Manuscripts

Optical Coherence Tomography Angiography for the detection of Neovascular Age-related Macular Degeneration: a Comprehensive Diagnostic Accuracy Study - the ATHENA study

Magdalena Niestrata [1], Jon Deeks [2], Yemisi Takwoingi [2], Sobha Sivaprasad [1], Praveen J. Patel [1], Pearse A. Keane [1], Ashleigh Kernohan [3], Luke Vale [3], Alastair K. Denniston [4], Richard Gale [5], Adam R. Khan [6], William McKinnon [6], Ridhi Agarwal [2], Gabriella de Salvo [7], Evangelos Minos [8], Paulo Barbeiro [9], Usha Chakravarthy [10], Nadia Waheed [11], Savita Madhusudhan [12], Tunde Peto [10], Konstantinos Balaskas [1]

1. NIHR Biomedical Research Centre at Moorfields Eye Hospital NHS Foundation Trust and UCL Institute of Ophthalmology, London, UK
2. Institute of Applied Health Research, University of Birmingham, Birmingham, UK
3. Population Health Sciences Institute, Newcastle University, Newcastle, UK
4. University Hospitals Birmingham NHS Foundation Trust, Birmingham, UK
5. York Teaching Hospital NHS Foundation Trust, York, UK
6. Birmingham Clinical Trials Unit, University of Birmingham, Birmingham, UK
7. Southampton University Hospital NHS Foundation Trust, Southampton, UK
8. North West Anglia NHS Foundation Trust, Hinchingbrooke and Peterborough, UK
9. Blueworks Medical Expert Diagnosis, Lisbon, Portugal
10. Queen's University Belfast, Belfast, UK
11. New England Eye Centre, Boston, USA
12. Liverpool University Hospitals NHS Foundation Trust, Liverpool, UK

Corresponding author: Konstantinos Balaskas, Moorfields Reading Centre and Clinical AI Lab, Moorfields Eye Hospital NHS Foundation Trust, 162 City Rd, London EC1V 2PD, k.balaskas@nhs.net

Wordcount: 3692

MeSH terms:

- Diagnostic Techniques, Ophthalmological
- Humans
- Reproducibility of Results
- Sensitivity and Specificity
- Tomography, Optical Coherence / methods
- Wet Macular Degeneration / diagnosis

For peer review only

Abstract

Introduction

The diagnosis of neovascular Age-related Macular Degeneration (nAMD), the leading cause of visual impairment in the developed world, relies on the interpretation of various imaging tests of the retina. These include invasive angiographic methods, such as Fundus Fluorescein Angiography (FFA) and, on occasion, Indocyanine-Green Angiography (ICGA). Newer, non-invasive imaging modalities, predominately Optical Coherence Tomography (OCT) and Optical Coherence Tomography Angiography (OCTA), have drastically transformed the diagnostic approach to nAMD. The aim of this study is to undertake a comprehensive diagnostic accuracy assessment of the various imaging modalities used in clinical practice for the diagnosis of nAMD (OCT, OCTA, FFA and, when a variant of nAMD called Polypoidal Choroidal Vasculopathy is suspected, ICGA) both alone and in various combinations.

Methods and analysis

This is a non-inferiority, prospective, randomised diagnostic accuracy study. Participants are patients with clinical features consistent with nAMD who present to a National Health Service (NHS) secondary care ophthalmology unit in the UK. Patients will undergo OCT as per standard practice and those with suspicious features of nAMD on OCT will be approached for participation in the study. Patients who agree to take part will also undergo both OCTA and FFA (and ICGA if indicated). Interpretation of the imaging tests will be undertaken by clinicians at recruitment sites. A randomised design was selected to avoid bias from consecutive review of all imaging tests by the same clinician. The primary outcome of the study will be the difference in sensitivity and specificity between OCT+OCTA and OCT+FFA (+/- ICGA) for nAMD detection as interpreted by clinicians at recruitment sites.

Ethics and dissemination

The study has been approved by the South Central - Oxford B Research Ethics Committee with reference number 21/SC/0412.

Dissemination of study results will involve peer-review publications, presentations at major national and international scientific conferences.

For peer review only

Strengths and limitations

Strengths

- Large multicentre comprehensive diagnostic accuracy study of all imaging modalities in common clinical use for nAMD diagnosis.
- The study design maximises its applicability to NHS clinical practice because test interpretation is made by clinicians at recruitment sites (rather than in silico lab conditions).
- First prospective, statistically powered diagnostic accuracy study to deliver a definitive comparison of FFA and OCTA for the diagnosis of nAMD

Limitations

- The study design is complex because randomisation for sequence of image review is required to prevent bias from consecutive interpretation of all imaging tests for each case by the same clinician at recruitment sites.
- The eligibility criterion of presence of nAMD suspicious features on OCT permits the assessment of Positive Predictive Value only for OCT but this study design ensures the feasibility of the recruitment plan.

Background and Study Aims

Age-related macular degeneration (AMD) is the most frequent cause of severe visual impairment and blindness in elderly populations in the developed world including the UK, Europe, the United States and Australia. [1-5] With age being the strongest risk factor for the development of AMD, the numbers of affected patients are steadily increasing in view of the aging population. [6] Consequently, the incidence of visual impairment and blindness caused by AMD has significantly increased from 1990 to 2010. [7] Worldwide, the number of patients affected by AMD is estimated to increase to 288 million in 2040. [8] [9]

AMD is categorised into non-neovascular or dry and neovascular or wet, based on the absence or presence of macular neovascularisation (MNV) [10] Neovascular AMD (nAMD) is characterised by fluid leakage or bleeding from new permeable vessels originating from the choroidal or deep retinal capillary network. MNV can be subclassified into type 1, otherwise known as occult, type 2, or classic and type 3 or retinal angiomatous proliferation (RAP), depending on the location of the MNV - beneath the retinal pigment epithelium (RPE), within the subretinal space and intraretinally, respectively. [11] [12]

In nAMD regardless of MNV subtype, treatment with anti-vascular endothelial growth factor (VEGF) therapy has been shown to be effective.[13-17] In those cases, early diagnosis and treatment is associated with improved visual prognosis and, therefore, improved patient quality of life and reduced morbidity. [18]

The diagnosis relies on imaging techniques visualising the retina and choroidal circulation. Optical Coherence Tomography (OCT), which has revolutionised the diagnosis and management of numerous retinal pathologies, features of nAMD disease activity including subretinal and intraretinal fluid, however, cannot directly detect a CNV. Until recently, FFA has been the gold-standard for the diagnosis of nAMD as it allows for visualisation of leakage from MNV. [9]

Nonetheless, this procedure is associated with the risk of complications, such as discoloration of urine, nausea and vomiting, and carries an extremely low risk of anaphylactic reaction. It is also contraindicated in kidney failure, which is not uncommon in elderly patients with systemic comorbidities who are investigated for nAMD. [19] The procedure is invasive, requiring intravenous cannulation and unpleasant for patients. In addition, the acquisition of images from FFA is time and labour-consuming, leading to significant difficulty due to limited resources and increasing numbers of patients requiring diagnostics.[1] Finally, occasionally FFA alone is not sufficient and additional imaging, such as Indocyanine-Green Angiography (ICGA) is required, particularly when Polypoidal Choroidal Vasculopathy (PCV), a variant of nAMD, is suspected.

For peer review only

ICGA has long been considered as the standard for PCV diagnosis and is generally considered as essential to make a definitive diagnosis of PCV.[20] Other imaging modalities have been assessed for the detection of PCV.[21] Fukuyama et al evaluated 62 polypoidal lesions detected on ICGA and 79% were detected on OCTA. [22] In a study which evaluated the SD-OCT findings of 188 eyes with PCV or neovascular AMD, results suggested that SD-OCT had a 89% sensitivity and 85% specificity in identifying PCV when 2 of the 3 signs were present: PED, double-layer sign, and thumb-like polyp.[23]

The advent of new non-invasive imaging techniques based on OCT, such as Optical Coherence Tomography Angiography (OCTA) has enabled a rapid direct visualisation of MNV without the need for intravenous contrast and without the side effects associated with FFA. [24-26]

Faes and colleagues conducted a systematic review and meta analysis of the performance of OCTA in detecting retinal disease. Over 1600 research articles involving OCTA have been published but only 17 studies were diagnostic accuracy studies. None of the studies were prospective and they provided scarce data on patient selection, data collection and analysis, masking, descriptions of the OCTA results and the reference standard. Out of the 17 studies, only 5 small studies evaluated detection of nAMD. These reported a wide range of sensitivity (0.50 to 1.00) and specificity (0.68 to 1.00), reinforcing the need for a well-designed diagnostic accuracy study to inform NHS decision-making on the role of OCTA. [27]

Cicinelli and colleagues (2020) have shown the overall sensitivity of OCTA for detection of active CNV to be 80.7% [28] In addition, the OCTA images have been shown to be less affected by subretinal haemorrhage compared to FFA. [29] [30] In terms of PCV detection, Cheung and colleagues showed a combination of structural OCT and OCTA achieved 82.6% sensitivity and 100% specificity [31] As a result, RCOphth guidance and some countries, such as France, have already changed their guidelines advocating increasing use of OCTA instead of FFA for nAMD diagnosis [32] However, there are no prospective, statistically powered, diagnostic accuracy studies that could inform the optimal diagnostic imaging protocol for nAMD.

Due to the lack of high quality evidence, there is variability in clinical practice with respect to the optimal imaging protocol for nAMD diagnosis. The original clinical interest in OCTA as a potential replacement of FFA in the diagnosis of nAMD has been superseded by the understanding that both techniques have relative and, on occasion, complementary merits, yet their optimal use for nAMD diagnosis remains unclear [27] The aim of ATHENA is to address these clinically relevant research questions

Methods

Study Design

The ATHENA study is a non-inferiority, prospective, randomised diagnostic accuracy study with an internal pilot to confirm feasibility of the recruitment plan.

For peer review only

Patient and Public Involvement

There was extensive patient and public involvement, as detailed in the supplementary elements.

Patient Population

Participants with a suspicion of nAMD in the first or second eye on OCT that present to an NHS secondary care ophthalmology unit in the UK will be recruited. They will be randomised to either OCT + FFA or OCT + OCTA study arms (**Figure 1**).

Study Procedures

In addition to OCT, each patient will undergo both OCTA and FFA. Patients with clinical or OCT appearance suggestive of PCV will also undergo ICGA at the same time as FFA (**Figure 1**).

In half the cases, selected randomly, an expert clinician at the recruitment site will review the OCT and then the OCTA and provide a clinical diagnosis (nAMD: yes/no). In the other half of the cases the site clinician will review the OCT and then the FFA and provide a clinical diagnosis (nAMD: yes/no) (CD1). (**Figure 1**) Subsequently, the same clinician will review the remaining imaging test in each case (FFA or OCTA) and will either confirm or change their initial clinical diagnosis (nAMD: yes/no) – recorded as clinical diagnosis 2 (CD2). (**Figure 1**) Comparison of the two clinical diagnoses within each arm will provide additional evidence concerning the added value of using a combination of both OCTA and FFA in patients with an OCT suggestive of nAMD.

Figure 1. Study design

Setting

Recruitment will take place in 20 hospital-based eye units across the UK. Of those 6 will be involved in the pilot study and all sites will be involved in the main study. The study coordinating centre (Trial Office) is the Birmingham Clinical Trials Unit (BCTU), based at the University of Birmingham.

Participants will be recruited amongst patients referred by their community optometrist, GP, or self-referred to secondary care eye casualty departments with a suspicion of nAMD and amongst patients already under the care of NHS eye units for an established diagnosis of nAMD in one eye who develop symptoms of it in the fellow eye. Using Eligibility criteria to define inclusion and exclusion criteria for potential patients **(Table 1).**

Eligibility Criteria

Table 1

Inclusion Criteria:	Exclusion Criteria:
Patients with suspicion of nAMD in the first or second eye who have:	Significant media opacities (cataract, vitreous opacities) that would not allow good quality fundus imaging;

For peer review only

Signed informed consent form;	Diabetic retinopathy of severity worse than mild non-proliferative stage and with any degree of diabetic maculopathy;
Ability to perform study specific procedures.	Other causes of choroidal neovascularisation (myopic, angioid streaks, inflammatory, retinal dystrophies, secondary to central serous chorioretinopathy, idiopathic);
	Inability to undergo dye-based imaging (FFA or ICGA) due to history of allergy.

Randomisation and Blinding

The purpose of randomisation in this study is to minimise bias in the comparative accuracy of OCT+OCTA and OCT+FFA (primary objective) that may arise from non-masked interpretation of the findings of OCTA and FFA by the same clinician. Randomisation will be provided by a secure online randomisation system at BCTU (http://www.trials.bham.ac.uk/athena), which will be available 24 hours a day, 7 days a week, apart from short periods of scheduled maintenance. The system will randomise each patient to one of the two study arms as described in study design (**Figure 1**).

A minimisation algorithm will be used within the online randomisation system to ensure balance in the treatment allocation over the following variables: the recruiting centre, whether the affected eye is the first or second eye and whether the case is suspicious of PCV. A 'random element' will be included in the minimisation algorithm, so that each patient has a probability of being randomised to the opposite study arm than they would otherwise be assigned to.

Sample size calculation

According to current literature, the sensitivity of OCT + OCTA and OCT + FFA for detection of nAMD is 92% [27] [33] [34] and 90% [35], respectively. Based on the above, 240 patients with nAMD will need to be recruited to ensure, with 90% certainty, that the upper limit of a one-sided 97.5% confidence interval (or equivalently a 95% two-sided confidence interval) will exclude a difference in favour of OCT+FFA of more than 10 percentage points (i.e. the non-inferiority margin). A non-inferiority margin of 10 percentage points was deemed appropriate in view of the expected advantages of OCTA (i.e. improved patient experience through the use of a quick non-invasive imaging technique) and an already established role in clinical practice.

Audit data from Moorfields Eye Hospital indicated a prevalence of 75% of confirmed nAMD in patients with a positive or suspicious OCT, based on which, 320 patients with suspected nAMD will be required. However, as the aim of the study is to compare specificity as well as sensitivity, using the same estimates as for sensitivity, it is anticipated that 960 patients will be required. Allowing for 10% withdrawal rate, missing data and inconclusive test results, we would aim to recruit a total of 1,067 patients.

For peer review only

Objectives - Outcome Measures and their Analysis

The primary analysis, against the objectives **(Table 2)**, will be based on a pragmatic approach to image interpretation for nAMD detection by clinicians at recruitment sites (comparison of OCT+OCTA versus OCT+FFA in patients with positive or suspicious OCT). This approach reflects real-life clinical practice and will allow a meaningful assessment of the diagnostic accuracy of the most commonly used confirmatory tests for nAMD detection in a clinical setting. This approach will enhance the potential for generalisability of study results in the NHS.

Figure 2: Methodological flowchart

All images (OCT, OCTA, FA and where available ICGA) will also be transferred to NetwORC UK Reading Centres Network using a secure web-based image management system. The accuracy of the individual imaging tests in isolation (OCT, OCTA, FA) will be assessed in a masked and random fashion by expert graders in the controlled environment of the Reading Centres. This will ensure the masked assessment of individual imaging tests independent of each other with the use of a sophisticated grading queue that ensures random allocation of tests and minimises bias. Grading of the images will be performed at the three Reading Centres by trained graders who have achieved a minimum of 90% agreement in the accreditation test, as per figure 2 above which highlights the unique methodology that combines an RCT (CONSORT diagram) with a Diagnostic Accuracy study (STARD diagram). The allocation of images will take into account the study design so that graders involved in grading of the reference standard will not be involved in grading the index tests. Dedicated image management software will be used for the purposes of aggregating imaging data from recruitment sites and developing grading queues for Reading Centre graders. The imaging tests will be presented in random order to the graders to avoid bias associated with grading images from the same patient in sequence. Graders will be masked to all clinical information as well as whether the imaging tests being reviewed originate from the same patient or not.

Objectives

Table 2

Primary Objective	Primary Outcome Measure

For peer review only

**The main aim of this study is to assess whether the sensitivity and specificity of OCTA combined with OCT is non-inferior to that of FFA combined with OCT, for the detection of neovascular age-related macular degeneration, in patients with clinical and OCT features suggestive of the diagnosis; as interpreted by clinicians.**		The primary outcome measure is the difference in sensitivity and specificity between OCTA combined with OCT and FFA combined with OCT. The unit of analysis is the patient (one 'trial' eye per patient). The sensitivity, specificity, PPV and NPV of the tests will be calculated. The diagnostic accuracy of OCT+OCTA and OCT+FFA will be compared to assess non-inferiority. The differences in sensitivity and specificity will be established and two-sided 95% confidence intervals calculated using the Wilson CI score method. If the lower boundary of the two-sided 95% confidence interval (CI) for the difference in sensitivity and specificity of OCT+OCTA relative to OCT+FFA is higher than −10 percentage points, the sensitivity of OCT+OCTA will be considered as non-inferior

For peer review

Secondary Objectives	Secondary Outcome Measures
**1. To assess the diagnostic accuracy of OCTA alone and FFA alone as reviewed by Reading Centre expert graders for the detection of nAMD in patients with a positive or suspicious OCT.**	Sensitivity, specificity, PPV and NPV of OCTA and FFA in isolation will be assessed on the basis of masked review by expert graders in the Reading Centres; 95% confidence intervals will be calculated using the Wilson method.
**2. To assess the positive predictive value (PPV) of OCT for the detection of nAMD in all patients presenting with suspicion of nAMD.**	The PPV of OCT for nAMD detection and its 95% CI will be estimated. Since only patients with a positive or suspicious OCT will be included, the sensitivity, specificity and NPV of OCT alone cannot be assessed.
**3. To compare the diagnostic accuracy of the combination of OCT+FFA versus OCT+FFA+OCTA as interpreted by retinal experts within the 'OCT+FFA' arm of the study.**	In order to establish the added diagnostic value of using a third imaging technique for nAMD detection, OCT+FFA will be compared against OCT+FFA+OCTA, in terms of differences in sensitivity and specificity together with their 95% Cis.

**4. To compare the diagnostic accuracy of the combination of OCT+OCTA versus OCT+FFA+OCTA as interpreted by retinal experts for the detection of nAMD within the 'OCT+OCTA' arm of the study.**	In order to establish the added diagnostic value of using a third imaging technique for nAMD detection, OCT+OCTA will be compared against OCT+OCTA+FFA, in terms of differences in sensitivity and specificity together with their 95% Cis.
**5. For a subset of cases with OCT and clinical features suspicious of Polypoidal Choroidal Vasculopathy (PCV) that underwent Indocyanine-Green Angiogrpahy (ICGA), to assess diagnostic accuracy of OCTA, FFA, ICGA, alone and in combinations, for the detection of PCV.**	For a subset of cases with clinical and OCT features suspicious of PCV, the diagnostic accuracy of OCTA, FFA and ICGA alone and in combination will be assessed by calculating their sensitivity, specificity, PPV and NPV (including their 95% CIs).
**6. To assess intra- and inter-rater agreement in the detection of nAMD on OCTA and FFA as assessed by Reading Centre graders.**	For OCTA and FFA, intra- and inter-rater agreement will be determined using percentage agreement and Gwet's first-order agreement coefficient (Gwet's AC1). [36] [37]
**7. To develop and validate criteria for the OCTA-based diagnosis of nAMD.**	The framework for OCTA-based nAMD diagnostic criteria created through this study will be assessed against a reference standard from the Reading Centres using all available multi-modal imaging (OCT, OCTA, FFA and ICGA where available) enhanced with clinical vignette describing patient symptoms and follow-up clinical data.
**8. To estimate the incremental cost per true positive detected and incremental cost per correct diagnosis for nAMD through a within study cost-effectiveness analysis.**	A model-based cost-effectiveness analyses (CEA) will be conducted to estimate the incremental cost per true positive detected and incremental cost per correct diagnosis for nAMD.
**9. To assess the diagnostic accuracy of OCTA by lesion type (PCV, type 1-, type 2-, type 3- nAMD).**	Sensitivity, specificity, PPV and NPV (including their 95% CIs) of OCTA for detection on nAMD by lesion type (PCV, type 1-, type 2-, type 3- nAMD) will be computed based on assessment by expert graders in the Reading Centres.
**10. To report limitations of OCTA use and adverse events.**	Number and classification of adverse events

For peer review only

Reference Standard

To provide the reference standard we will use all available imaging information from multi-modal imaging (OCT, OCTA, FFA and ICGA where available) enhanced with clinical vignette describing patient symptoms and available follow-up clinical data. The images will be interpreted by Reading Centre graders different to those involved in the masked assessment of the individual imaging tests and they will review all imaging and clinical information for each patient. Uncertain cases by primary grading will be reviewed by a senior adjudication panel (directors of the three Reading Centres) who will provide the reference standard in these cases.

Two versions of the reference standard will be provided:

- RS1 based on all imaging modalities except OCTA
- RS2 based on all imaging modalities including OCTA

This will allow assessment of the potential effect of incorporation bias from inclusion of the primary index test (OCTA) in determining the reference standard. The primary analyses will be based on RS1.

Economic Evaluation

A within study cost-effectiveness analysis (CEA) will be conducted to estimate the incremental cost per true positive detected and incremental cost per correct diagnosis for nAMD. The interventions to be analysed will include OCT+FA, OCT+OCTA and OCT+FA+ICGA, given PCV. Estimates of diagnostic performance will be based on the study analysis and costs will be estimated from the NHS and PSS perspective up to the end of the diagnostic phase. Detailed methodology of the cost-effectiveness analysis is described in the Supplementary Material (Page 3)

A decision model will also be developed. The decision model will facilitate the comparison of alternative strategies made up of alternative testing strategies (i.e. different combination of tests, different rules for diagnosis or subsequent testing). The structure of the model is likely to take the form of a decision tree. The model will use the data collected within the clinical study and the within study economic evaluation. The time horizon for the model-based analysis will cover the diagnostic testing period only and results will be presented using the same outcomes as presented above. This strategy will produce a cost per true positive or cost per avoidance of false negative for the strategies assessed. Both the within study CEA and decision model will aid in the identification of the most efficient strategy for identifying cases of sight threating nAMD.

Data Management

Processes will be employed to ensure the accuracy of data included in the final report. Coding and validation will be agreed on by the study manager, statistician and programmer; and the study database will be signed off once the implementation of these has been assured.
Data will be entered onto the study database by staff involved in the study or delegated staff at sites. Data entries within the electronic database will be scrutinised for completeness and accuracy, with discrepancies checked against paper data forms. The data

For peer review only

capture system will conduct automatic range checks for specific data values to ensure high levels of data quality. Any queries raised will be registered using the integrated data query system in the study database, with the expectation to be resolved within 30 days.
Every attempt will be made to collect full imaging data on all study participants, and the proportion of missing data is expected to be minimal. Participants with indeterminate or missing results will not be included in the primary analysis.

Study Termination

The end of study will be 6 months after the last data capture. This will allow sufficient time for the completion of protocol procedures, data collection and data input. The Trials Office will notify the Research Ethics Committee (REC) that the study has ended within 90 days of completion. In the instance of terminating the study early, the Trials Office will inform the REC within 15 days of study termination. The REC and Trial Office will receive a summary of the clinical study report within 12 months of study completion.

Consent and Ethics

The study will be performed in accordance with the recommendations guiding physicians in biomedical research involving human subjects, adopted by the 18th World Medical Association General Assembly, Helsinki, Finland, 1964, amended by the 48th WMA General Assembly, Somerset West, Republic of South Africa, 1996
Favourable opinion has been provided by the Research Ethics Committee and the study has received approval by the UK Health Research Authority.
Study participants will be required to give informed consent and sign a consent form. Trial participation is entirely voluntary, and the consent may be withdrawn at any time without this affecting patients' care.

Dissemination

Study dataset dissemination

The final pseudonymised study dataset will be made available to members of the study management group so that publications describing the outcomes (Table 2) can be prepared. Following publication of findings, the fully anonymised study dataset will be made available to external researchers upon approval from the study management group, the study steering committee, and the Sponsor in line with best data sharing practices for clinical study datasets.

Dissemination of Results

Results of this study will be submitted for publication in high impact peer reviewed journals.

The study question is important and has the potential to change clinical practice. Should the study results support this, the change in practice is expected to be rapidly incorporated into guidelines including the National Institute of Health and Care Excellence (NICE) and the Scientific Advisory Committees in the Department of Health.

The findings will be presented and disseminated via national and international conferences. A copy of the monograph will be lodged with the NIHR Journals Library as a permanent and comprehensive record of the study. In consultation with the investigators

For peer review only

and relevant journal, a press release will be issued to the media upon publication of the results.

Results of the study will be shared with study participants, staff members at research sites and investigators of other studies related to any of the imaging techniques investigated in ATHENA. Outreach to other key stakeholders (study networks, health advocates) involved in related studies is planned.

Acknowledgement and Contributors

The research team expresses its gratitude to the Study Oversight Committees for their valuable expert advice, steer, and support throughout the entire duration of the study.

Study Steering Committee: Rupert Bourne (Chair, Cambridge University Hospital and Anglia Ruskin University), Catey Bunce (The Royal Marsden NHS Foundation Trust), Geraldine Hoad (the Macular Society).

Data Monitoring Committee: Yit Yang (Chair, The Royal Wolverhampton NHS Trust), Irene Stratton (Gloucestershire Hospitals NHS Foundation Trust), Tomas Cudrnak (University Hospitals Plymouth NHS Foundation Trust).

We would also wish to acknowledge the valuable contribution to study pathway development and delivery of the Moorfields Ophthalmic Reading Centre team: Khadra Adan, Rosana Lima, Georgina Wignall, Alexandra Winston, Afreen Begum.

Conflicts of Interest

None

Funding Statement

The Sponsor for this study is Moorfields Eye Hospital NHS Foundation Trust who are also the Head Organisation (i.e. the contracting party with the funder).

Funding for the ATHENA study is provided by an award from the National Institute of Health Research, Health Technology Assessment Programme with Reference: NIHR131432.

References

1. Coleman HR, Chan CC, Ferris FL, Ferris 3rd CEY. Age-related macular degeneration. Lancet. 2008; 372(9652): 1835-45.

For peer review only

2. Lim LS, Mitchell P, Seddon JM, Holz FG, Wong TY. Age-related macular degeneration. Lancet. 2012; 379(9827): 1728-38.

3. Klein R, Klein BEK, Tomany SC, Meuer SM, Huang GH. Ten-year incidence and progression of age-related maculopathy: The Beaver Dam eye study. Ophthalmology. 2002; 109(10): 1767-79.

4. Wang JJ, Rochtchina E, Lee AJ, Chia EM, Smith W, Cumming R, et al. Ten-year incidence and progression of age-related maculopathy: the blue Mountains Eye Study. Ophthalmology. 2007; 114(1): 92-8.

5. Economics A. The economic impact of sight loss and blindness in the UK adult population. ; 2019.

6. Colijn JM, Buitendijk GHS, Prokofyeva E, al. e. Prevalence of Age-Related Macular Degeneration in Europe: The Past and the Future. Ophthalmology. 2017; 124(12): 1753-1763.

7. Jonas JB. Global prevalence of age-related macular degeneration.. Lancet Glob Health. 2014; 2(2): 65–66.

8. Wong WL, X S, Li X, Cheung CM, Klein R, Cheng CY, et al. Global prevalence of age-related macular degeneration and disease burden projection for 2020 and 2040: a systematic review and meta-analysis.. Lancet Glob Health. 2014; 2(2): 106–116.

9. Deng Y, Qiao L, Du M, Qu C, Wan L, Li J, et al. Age-related macular degeneration: Epidemiology, genetics, pathophysiology, diagnosis, and targeted therapy. Genes Dis. 2022; 9(1): 62–79.

10. Bird AC, Bressler NM, Bressler SB, Chisholm IH, Coscas G, Davis MD, et al. An international classification and grading system for age-related maculopathy and age-related macular degeneration. The International ARM Epidemiological Study Group. Surv Ophthalmol. 1995; 39(5): 367-74.

11. Bae K, Noh SR, Kang SW, Kim ES, Yu SY. Angiographic Subtypes of Neovascular Age-related Macular Degeneration in Korean: A New Diagnostic Challenge. Sci Rep. 2019; 9(1): 9701.

12. Farecki ML, Gutfleisch M, Faatz H, Rothaus K, Heimes B, Spital G, et al. Characteristics of type 1 and 2 CNV in exudative AMD in OCT-Angiography. Graefes Arch Clin Exp Ophthalmol. 2017; 255(5): 913-921.

13. Brown DM, Kaiser PK, Michels M, Soubrane G, Heier JS, Kim RY, et al. Ranibizumab versus verteporfin for neovascular age-related macular degeneration. N Engl J Med. 2006; 355(14): 1432-44.

14. Agarwal A, Aggarwal K, Gupta V. Management of Neovascular Age-related Macular Degeneration: A Review on Landmark Randomized Controlled Trials. Middle East Afr J Ophthalmol. 2016; 23(1): 27-37.

15. Oishi A,KH,MM,HS,MT, Oh H, Kita M, Nagai T, Fujihara M, Bessho N, et al. Comparison of the effect of ranibizumab and verteporfin for polypoidal choroidal vasculopathy: 12-month LAPTOP study results. Am J Ophthalmol. 2013; 156(4): 644-51.

16. Heier JS, Brown DM, Chong V, Korobelnik JF, Kaiser PK, Nguyen QD, et al. Intravitreal aflibercept (VEGF trap-eye) in wet age-related macular degeneration. Ophthalmology. 2012; 119(12): 2537-48.

For peer review only

17. Tufail A, Patel P, Egan C, Hykin P, da Cruz L, Gregor Z, et al. Bevacizumab for neovascular age related macular degeneration (ABC Trial): multicentre randomised double masked study. BMJ. 2010.

18. Wong T, Chakravarthy U, Klein R, Mitchell P, Zlateva G, Buggage R, et al. The natural history and prognosis of neovascular age-related macular degeneration: a systematic review of the literature and meta-analysis. Ophthalmology. 2008; 115(1): 116-26.

19. Gess AJ, Fung AE, Rodriguez JG. Imaging in Neovascular Age-Related Macular Degeneration. Semin Ophthalmol. 2011; 26(3): 225-33.

20. Tan CS NWCJTNLT. EVEREST study report 2: imaging and grading protocol, and baseline characteristics of a randomised controlled trial of polypoidal choroidal vasculopathy. Br J Ophthalmol. 2015; 99(1): 624–628.

21. Koh AH CLCSea. Expert PCV Panel Polypoidal choroidal vasculopathy: evidence-based guidelines for clinical diagnosis and treatment. Retina. 2013; 33(1): 686–716.

22. Fukuyama H IHATIHINGF. Indocyanine green dye filling time for polypoidal lesions in polypoidal choroidal vasculopathy affects the visibility of the lesions on OCT angiography. Ophthalmol Retina. 2018; 2(1): 803–807.

23. Liu R LJLZea. Distingishing polypoidal choroidal vasculopathy from typical neovascular age-related macular degeneration based on spectral domain optical coherence tomography. Retina. 2016; 36(1): 778–786.

24. Coscas G, Lupidi M, Coscas F. Image Analysis of Optical Coherence Tomography Angiography. Dev Ophthalmol. 2016; 56: 30.

25. de Carlo TE, Romano A, Waheed NK, Duker JS. A review of optical coherence tomography angiography (OCTA). Int J Retina Vitreous. 2015; 1: 5.

26. Rocholz R, Corvi F, Weichsel J, Schmidt S, Staurenghi G. OCT Angiography (OCTA) in Retinal Diagnostics. In Bille JF, editor. High Resolution Imaging in Microscopy and Ophthalmology: New Frontiers in Biomedical Optics.: Springer; 2019.

27. Faes L, Bodmer N, Locher S, Keane PA, Balaskas K, Bachmann LM, et al. Test performance of optical coherence tomography angiography in detecting retinal diseases: a systematic review and meta-analysis. Eye (Lond). 2019; 33(8): 1327-1338.

28. Cicinelli MV, Cavalleri M, Consorte A, Rabiolo A, Sacconi R, Bandello F, et al. Swept-source and spectral domain optical coherence tomography angiography versus dye angiography in the measurement of type 1 neovascularization. Retina. 2020; 40(3): 499-506.

29. Moult E, Choi W, Waheed N, Adhi M, Lee B, Lu C, et al. Ultrahigh-speed swept-source OCT angiography in exudative AMD. Ophthalmic Surg Lasers Imaging Retina. 2014; 45(6):496-505(45(6):496-505): 45(6):496-505.

30. Deng Y, Qiao L, Du M, Qu C, Wan L, Li J, et al. Quantitative optical coherence tomography angiography of choroidal neovascularization in age-related macular degeneration. Ophthalmology. 2014; 121(7): 1435–1444.

For peer review only

31. Cheung CMG, Yanagi Y, Akiba M, Tan A, Mathur R, Chan CM, et al. Improved detection and diagnosis of polypoidal choroidal vasculopathy using a combination of optical coherence tomography and optical coherence tomography angiography.. Retina. 2019; 39(9): 9.

32. Wolff B, De Bats F, Tick S, Cornut P, Souied É, Cohen S. Update from France Macula Federation: Diagnosis of wet AMD. J Fr Ophtalmol. 2018; 41(9): 857-861.

33. Faridi A, Jia Y, Gao SS, Huang D, Bhavsar K, Wilson DJ, et al. Sensitivity and Specificity of OCT Angiography to Detect Choroidal Neovascularization. Ophthalmol Retina. 2017; 1(4): 294-303.

34. Gong J, Yu S, Gong Y, Wang F, Sun X. The Diagnostic Accuracy of Optical Coherence Tomography Angiography for Neovascular Age-Related Macular Degeneration: A Comparison with Fundus Fluorescein Angiography. J Ophthalmol. 2016.

35. Gualino V, Tadayoni R, Cohen SY, Erginay A, Fajnkuchen F, Haouchine B, et al. OPTICAL COHERENCE TOMOGRAPHY, FLUORESCEIN ANGIOGRAPHY, AND DIAGNOSIS OF CHOROIDAL NEOVASCULARIZATION IN AGE-RELATED MACULAR DEGENERATION. Retina. 2019; 39(9): 1664-1671.

36. K. G. Computing inter-rater reliability and its variance in the presence of high agreement. British Journal of Mathematical and Statistical Psychology. 2010; 60(1): 29-48.

37. De Raadt A WMBRKH. Educational and Psychological Measurement. Educ Psychol Meas. 2019; 79(3): 558-576.

For peer review only

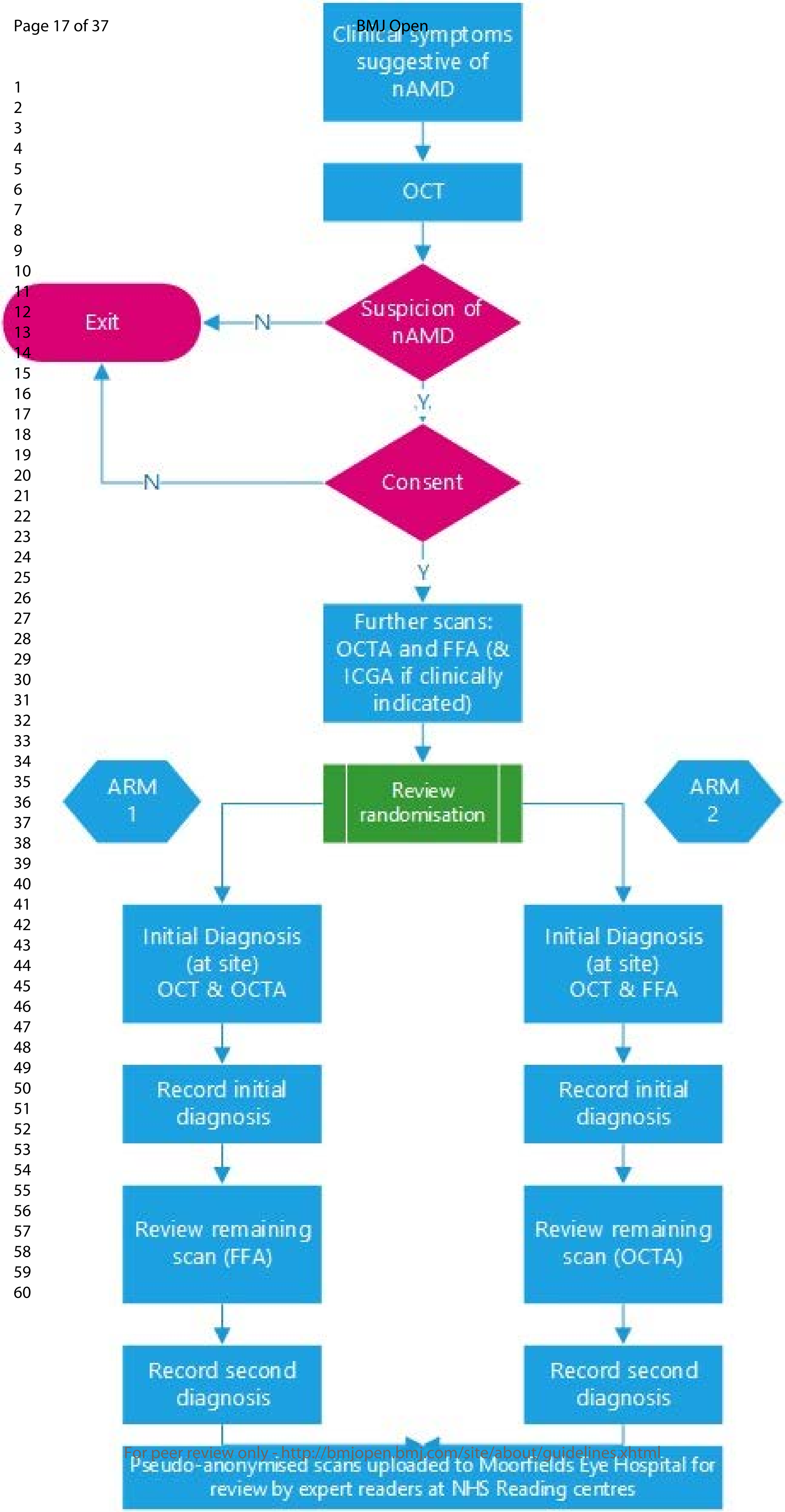

For peer review only - http://bmjopen.bmj.com/site/about/guidelines.xhtml

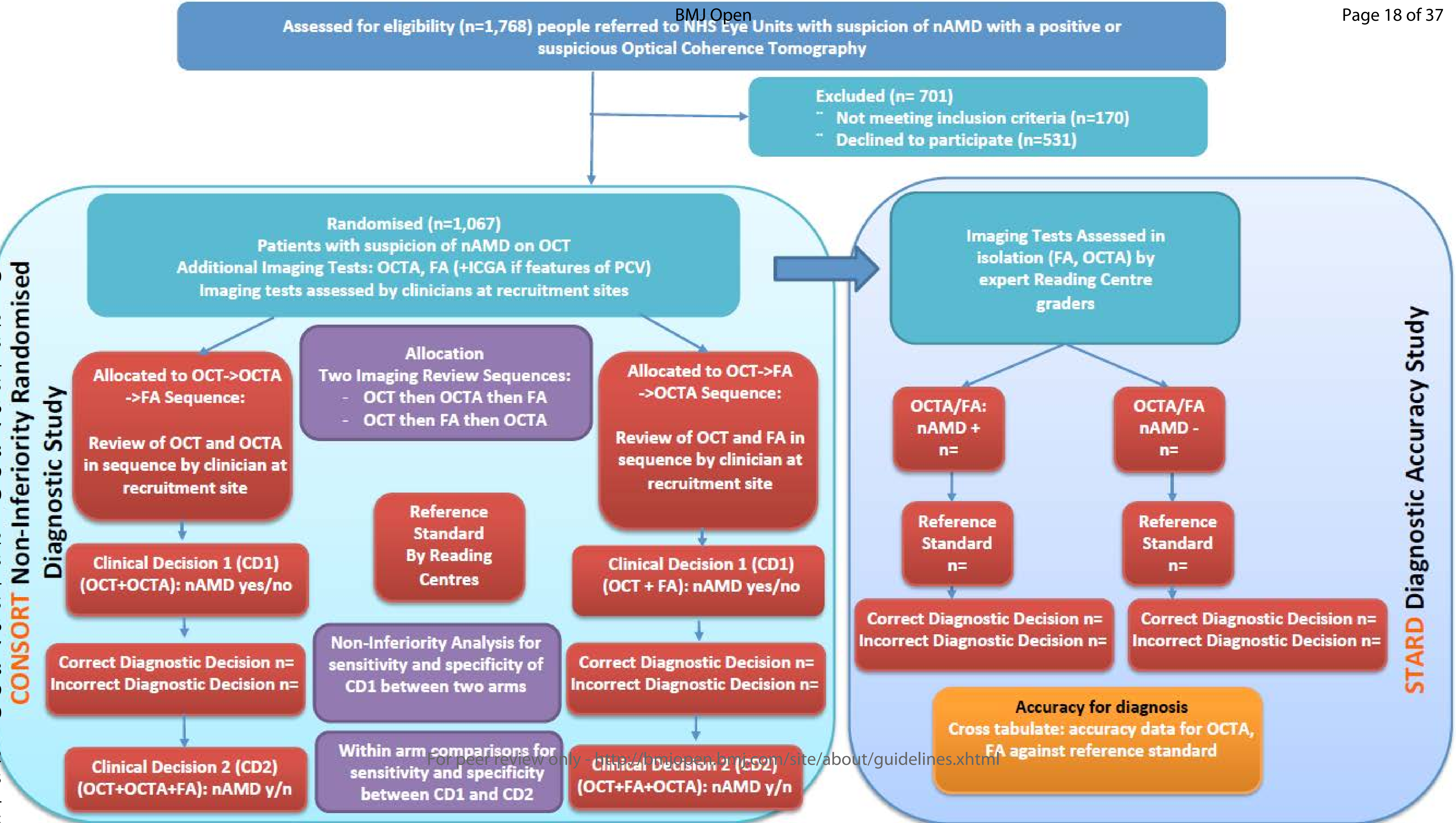
Assessed for eligibility (n=1,768) people referred to NHS Eye Units with suspicion of nAMD with a positive or suspicious Optical Coherence Tomography
Excluded (n= 701)
Not meeting inclusion criteria (n=170)
Declined to participate (n=531)
CONSORT Non-Inferiority Randomised Diagnostic Study
Randomised (n=1,067)
Patients with suspicion of nAMD on OCT
Additional Imaging Tests: OCTA, FA (+ICGA if features of PCV)
Imaging tests assessed by clinicians at recruitment sites
Allocated to OCT->OCTA ->FA Sequence:
Review of OCT and OCTA in sequence by clinician at recruitment site
Allocation
Two Imaging Review Sequences:
- OCT then OCTA then FA
- OCT then FA then OCTA
Allocated to OCT->FA ->OCTA Sequence:
Review of OCT and FA in sequence by clinician at recruitment site
Reference Standard By Reading Centres
Clinical Decision 1 (CD1) (OCT+OCTA): nAMD yes/no
Clinical Decision 1 (CD1) (OCT + FA): nAMD yes/no
Correct Diagnostic Decision n=
Incorrect Diagnostic Decision n=
Non-Inferiority Analysis for sensitivity and specificity of CD1 between two arms
Correct Diagnostic Decision n=
Incorrect Diagnostic Decision n=
Clinical Decision 2 (CD2) (OCT+OCTA+FA): nAMD y/n
Within arm comparisons for sensitivity and specificity between CD1 and CD2
Clinical Decision 2 (CD2) (OCT+FA+OCTA): nAMD y/n
STARD Diagnostic Accuracy Study
Imaging Tests Assessed in isolation (FA, OCTA) by expert Reading Centre graders
OCTA/FA: nAMD + n=
OCTA/FA nAMD - n=
Reference Standard n=
Reference Standard n=
Correct Diagnostic Decision n=
Incorrect Diagnostic Decision n=
Correct Diagnostic Decision n=
Incorrect Diagnostic Decision n=
Accuracy for diagnosis
Cross tabulate: accuracy data for OCTA, FA against reference standard

Supplementary Material

Contents

Contents	Page
Expertise of the Research Team	2
Success Criteria and Barriers to proposed work	2
Study Management and Oversight	2
Timelines	3
Economic Evaluation: Methodology	3
Training of Reading Centre graders and Clinicians at Recruitment Sites	4
Data Collection and Management	4
Image Management and Grading Process	5-6
Patient and Public Involvement	6-7
Consent Form	8-10
Patient Information Sheet	11-20

For peer review only

Expertise of the Research Team:
A multidisciplinary team covering the core competencies is combining forces to guarantee successful delivery of all aspects of the trial. The trial is supported by the Birmingham CTU. The Chief Investigator has extensive experience in leading multi-site NIHR funded research projects and is director of the Moorfields Ophthalmic Reading Centre. The directors of Belfast and Liverpool Reading Centres are also co-applicants, Prof Tunde Peto as lead co-applicant given her extensive expertise in imaging research in the UK and Savita Madhusudhan, respectively. Clinician academics with track record on imaging and OCTA research as well as clinical trial methodology (Pearse Keane, Praveen Patel, Sobha Sivaprasad, Alastair Denniston, Richard Gale) are combining efforts for the design and delivery of the study. Test evaluation and statistical expertise will be provided by Prof Jon Deeks and Prof Yemisi Takwoingi, recognised international experts in this field. Economic evaluation is delivered by Dr Ashleigh Kernohan at the University of Newcastle under the expert mentorship and supervision of Prof Luke Vale, an internationally recognised expert in the field. Prof John Lawrenson, City University of London will support and supervise the delivery of the on-line training programme on OCTA interpretation for clinicians at recruitment sites. Prof Chakravarthy, a senior academic of international standing, will support the study as collaborator and will co-ordinate activities of the International OCTA Nomenclature group. A distinguished member of that group and recognised world expert on OCTA research, Prof Nadia Waheed, University of Boston, USA is joining the team as collaborator. A detailed plan on PPI involvement is provided separately.

Success Criteria and Barriers to proposed work:
Success criteria:
- Timely identification and set-up of recruitment sites Recruitment to time and target
- Complete imaging datasets (OCT, FA, OCTA and when appropriate ICGA) for each patient

Risks:
- Difficulties with recruitment due to local R&D set-up delays. Unwillingness of sites to undertake FA in all cases of suspected nAMD
- Risks associated with the COVID-19 pandemic (study set-up, recruitment, changes in routine care)

  Mitigation Strategy:
- Efficient project management to assist sites with set-up and monitor performance
- Strong links of research team with ophthalmology departments nationally and track record of successful delivery of multi-site NIHR funded research
- Close engagement of the research team with local Clinical Research Networks

Study Management and Oversight:
The overall management structure of this study will consist of a Trial Management Group (TMG), a Trial Steering Committee (TSC) and a Data Monitoring Committee (DMC). The TMG will be responsible for the day-to-day running and management of the trial. The Group will meet formally and informally regularly to discuss progress of the trial and examine mitigating strategies in case of issues arising.
The TSC will ensure the overall integrity of the study by monitoring its progress and taking account of regular reports from the DMC and TMG. The TSC will meet during the development phase, at the end of the pilot and then annually (or more often, if determined by the Chair). The TSC will have an independent chair. The TSC will also include a representative of the Macular Society.
The independent DMC will monitor trial data to ensure that the trial is being implemented in accordance with the highest standards of patient safety and ethical conduct. Throughout the trial, the DMC will monitor data on recruitment, adverse events, emerging external evidence, sample characteristics and make recommendations on whether an interim analysis is required.

For peer review only - http://bmjopen.bmj.com/site/about/guidelines.xhtml

For peer review only

Timelines:

- Development phase: 6 months
- Internal Pilot: 6 months
- Main study: 18 months
- Reading Centre adjudication and quality assurance, statistical analysis, economic analysis, reporting of results: 6 months

Economic Analysis (Cost-Effectiveness Analysis Methodology):

The costs of the different tests will be based upon micro costing of the resources needed to deliver the tests (Drummond 2015). The type and frequency of resources used to diagnose nAMD will be collected using a bespoke Case Report Form (CRF). This will include costs for outpatient appointments, procedures (e.g. injections of diagnostic dyes) and different ocular imaging techniques (e.g. OCT, OCTA and FA). Any drugs required in the process of diagnosis will also be included, these costs will be derived from the British National Formulary [1]. Cost estimates for the resources will be derived from published sources such as NHS reference costs and Unit Costs of Social Care [2, 3]. Where costs are not available, a micro-costing approach will be used to estimate costs using data collected from the CRFs and expert clinical opinion of the co-applicants. Regression techniques will be applied to the costs data to determine any difference in costs between/among interventions.

Sensitivity analysis will be carried out using both deterministic and stochastic approaches. The sensitivity analysis will be used to assess parameter uncertainty, including any difference identified between the hospital based diagnostic accuracy study and the diagnostic accuracy study based in the Reading Centres. Deterministic sensitivity analysis is used to explore plausible alternatives (for example, quantity of resources used or unit costs) and stochastic or probabilistic sensitivity analysis is used to explore statistical imprecision in estimates of costs, effects and cost-effectiveness.

For peer review only

For peer review only - http://bmjopen.bmj.com/site/about/guidelines.xhtml

Training of Reading Centre graders and Clinicians at Recruitment Sites:

The grading of stand-alone imaging tests (OCT, OCTA, FA, ICGA) collected in the context of this study will be performed by trained graders based at NetwORC UK Consortium (Moorfields Reading Centre, Belfast Reading Centre, and Liverpool Reading Centre). The Reading Centres will also provide the reference standard for all diagnostic accuracy assessments. The training and accreditation process will be overseen by the three Reading Centre directors and will follow established standard operating procedures for the training of graders involved in ophthalmic imaging research (including training and accreditation datasets, accreditation thresholds and resit opportunities, accreditation maintenance and assessment of temporal drift for graders). The Reading Centres will develop image acquisition protocols for all imaging modalities collected in this study to ensure consistency of image acquisition.

The Moorfields Reading Centre (https://readingcentre.org) in collaboration with City, University of London (Prof John Lawrenson) will establish an on-line training platform for the training of clinicians (retinal experts) at recruitment sites in the interpretation of OCTA. The same interpretation protocol used for the training of the Reading Centre graders will form the basis of the clinician training programme. Clinicians (retinal experts) are very familiar with the interpretation of OCT and FFA for the detection of nAMD and in the pragmatic context of this study design, no additional training in the interpretation of these imaging modalities will be provided. Given, however, the lack of consensus on the interpretation of OCTA and the objective of this study to develop and validate such a consensus interpretation protocol, we will provide training for clinicians on OCTA interpretation to ensure consistency and allow a credible assessment of diagnostic accuracy.
The training for ATHENA will incorporate the same open-source online learning platform 'Moodle', to provide relevant learning materials for OCTA image interpretation/standardisation. Training materials will include a combination of online lectures and webinars to describe the consensus interpretation protocol.

Data Collection and Management:

The Birmingham CTU team will provide input at all stages of the application. A trial manager will take responsibility for the day-to-day co-ordination of the project, and assist in obtaining research approvals, maintaining the trial master file, monitoring recruitment rates and protocol compliance, and reporting any adverse events. A team leader will provide oversight of the trial, and a quality monitor will provide governance oversight. An analyst programmer will support development and maintenance of the database to capture baseline and outcome data, for contemporaneous data cleaning, to produce reports and maintain a full audit trail. A data manager will help with chasing missing and inconsistent data. An analyst programmer, under the guidance of the programming team lead, will develop a bespoke database. The programming team will also assist in producing reports and maintain a full audit trail relating to database development and any subsequent changes. Trial data will be retained for a minimum of 25 years in Birmingham archives and the trial database will be legacy archived as per the University of Birmingham archiving policy.

For peer review only - http://bmjopen.bmj.com/site/about/guidelines.xhtml

Image Management and Grading Process:

The Moorfields Ophthalmic Reading Centre in partnership with an experienced image management software developer ('Blueworks') will develop and maintain a bespoke image transfer and management platform. This will allow secure transfer of data from recruitment sites to the Reading Centres and facilitate the grading process by providing sophisticated grading queues with embedded rules to minimise bias and inform the inter- and intra-grader agreement study outcomes.
The bespoke software will enable critical functions of study imaging data collection, transfer, and processing. This will include the following functionalities:
1. Simplified upload and anonymisation: The system will provide photographers with a simple interface allowing to select study-related exams from a list of exams on the more commonly used imaging devices for OCTA, OCT, FA and ICGA (Topcon, Heidelberg), and will pick up on user exported files for the same exams on Zeiss and Optovue systems. In both cases, users will select details to use on anonymisation, and auto-upload exams to the trial coordination centre (Moorfields Reading Centre) via SFTP (Secure File Transfer Protocol), in a way that allows contents to be audited regarding origin.
2. Automated download and validation of single exams: A functionality of the software will monitor the SFTP and check for new uploads. Each upload detected will be recorded into a "staging database", including details on the source SFTP, and the files will be moved from SFTP to a secure folder on the Trial Coordination Server. The software component monitoring the staging database will apply at least the following validation rules and the outcomes will be stored on the staging database:

- If FFA or ICG - sequence of laterality + time interval rules
- If OCT - number of scans, anatomical size, scanned area

3. Automated validation and integration of multiple single exams into unified trial records: The software at the trial coordination centre will process the contents of valid exams on the staging database, and aggregate those as a single Study ID session of multiple exams. The following validation rules will be automatically applied on the imaging tests:

- Log errors if Study ID details are not a precise match across all devices
- Log error if for a Study ID with OCTA, no ICGA or FFA exists, or if for ICGA or FFA, no OCTA exists
- Log error if exams do not match on eyes (for example, OCT for both eyes and FFA/ICGA just for one, or FFA/ICGA for both eyes but OCT just for one)

4. Automated reporting to trial coordination staff of validation failures/successes: A software tool will run at the trial coordination centre which will expand the staging database to contain NHS e-mail addresses of key contacts at each site and monitor uploaded data. The tool will send daily summary of uploaded data to the local trial coordinator, and to each uploader, on the data they have uploaded. The tool will keep a matched table between type of error and type of alarm to trigger and send e-mails to each uploader with detected errors with periodicity as defined on type of alarm.

5. Grading platform: A bespoke grading interface will be provided through the Image Management Software, allowing to collect grading outcomes and validate them for basic filling typos. An advanced rule-based system will define the type and sequence of exams to display to each grader. Exams are displayed one at a time with ability to navigate the multiple images of each FFA, ICGA or OCT volume, and if desired temporarily manipulate them (e.g. zoom, pan, OCT colour, adjustments to brightness, contrast, gamma and histogram). It will only display Study ID but not the exam date or site where the images were acquired. For OCTA exams, the system will display the message "please

For peer review only - http://bmjopen.bmj.com/site/about/guidelines.xhtml

check this exam on the [Heyex / ImageNet / Forum / Other] system by searching for Study ID."
The platform will support the following grading tasks:

- Single grading with request for adjudication
- 20% re-grading for intra-grader agreement (exam presented to the same grader some time after the initial grading)
- 20% random double grading by different graders for inter-grader agreement o 10% of random single grading directed to adjudication for quality assurance
- The graders will not be able to grade more than one imaging modality for the same Study ID, except if the last exam seen for that Study ID had been 10 hours earlier, or the grader had since graded another 20 exams.

Patient and Public Involvement

Pre-application Stage

In preparation for this proposal, A PPI event was held at Queen's University Belfast under the supervision of Prof Tunde Peto. This involved patients with retino-choroidal disease, including nAMD. A sub-group of PPI contributors with nAMD and previous experience of undergoing FA was identified and invited to participate in a faciliated group discussion. The research question and key concepts of the study design were discussed with the group. The facilitated discussion was informed by guidance provided by the Moorfields Biomedical Research Centre Patient and Public Involvement and Education group (PPIE) and included the following topics:

1. What are your experiences of Fluorescein Angiography?

What do you like? What do you dislike?

2. Do you have any concerns or anxieties when you are coming in for a Fluorescein Angiography appointment?

Number of procedures

Side effects of the dye (especially nausea, yellow colouring of the skin) Length of appointment

3. When you are asked to consent to the procedure are you clear what procedure you are consenting to?

Do you consent to more than one procedure?

Is the consent process clearly explained?

Is the process confusing or distressing in any way?

4. If we could capture the same information by a different method, what would your main priorities be? Speed

Safety

Invasive/non-invasive

Convenience

For peer review only - http://bmjopen.bmj.com/site/about/guidelines.xhtml

PPI contributors expressed a strong preference towards non-invasive diagnostic procedures and confirmed that the first hospital visit for diagnosis of nAMD is often long and confusing, mainly due to the inconvenient and time-consuming nature of FA. When looking at an alternative test to replace FA, PPI contributors would want to ensure the new test is as good as the existing one for a safe diagnosis- once this is ensured, their priorities for the new test in order of preference were: non-invasive; convenient; fast. PPI contributors also expressed a preference for a pragmatic study design that will ensure the research has a direct practical implication for patient care. This informed the selection of the primary objective for this study; the review of imaging tests by clinicians in hospital sites who are directly providing care for patients with nAMD.

PPI contributors would welcome a non-invasive test to replace the unpleasant experience of FA and help clinicians confirm the diagnosis of nAMD faster and start vision-saving treatment sooner. In the open-ended question ‘what would help you gain greater trust in the new test’ several patients placed emphasis on patient information to be provided prior to their appointments explaining the new test and its potential benefits. Captured comments from patients will be crucial for ensuring that information material is appropriate and suitably worded to infrom on the proposed research. They will also help determine the most appropriate strategies for approaching patients to discuss participation in the study and ensure patients have adequate time to consider fully the patient information material and have any queries addressed before deciding on participation.

The Lay Summary was shared with 11 PPI contributors through established links with the MoorfieldsBRC PPIE group. 8 patients returned valuable feedback that was incorporated in the summary. All patients were happy that the Lay Summary is clear, understandable and written in appropriately plain language and that all jargon is adequately explained.

During Study Delivery

- Development phase: Taking on board recommendations from the pre-application PPI event, PPI representative on the TSC will review draft patient information material, advise on wording and provide recommendations to the TSC. A meeting of the TSC is planned at the onset of the development period of the study (first 6 months of the study) either face-to-face or via teleconference. The TSC, on advice from its PPI member, will review the study protocol, consider potential barriers to recruitment, issues around location and geographical spread of recruitment sites and transport arrangements and any other elements that would enhance patient trust.

- Pilot and Main study: A study-specific PPI group will be recruited at Moorfields, where the study principle investigators are based. The group will consist of 4-6 PPI contributors recruited through existing links at Moorfields BRC and by inviting suggestions from the eye charities (Action Against AMD, Macular Society) for patient experts engaged in educational and patient support initiatives of the charities. The PPI contributor sitting on the TSC will be invited to also participate in the study-specific PPI group. Their role will involve contribution to training of PPI group members. The study-specific PPI group will meet at the end of the pilot phase to consider any potential barriers to recruitment and contribute feedback to the TSC to inform the decision on progression to the main study. The PPI group will also transition to the main study and will meet at least once a year with provision for additional face-to-face or ‘virtual’ meetings when input is required for potential protocol amendments or issues arising during the study. An end of study debrief is planned with all PPI contributors which will include discussions of the prioritisation and dissemination of study results to patients and the public.

For peer review only

Consent Form

<Insert local trust header> Participant Trial Number: ☐☐☐☐☐☐

OpticAl Coherence TomograpHy Angiography for the dEtection of Neovascular Age-related Macular Degeneration a Comprehensive Diagnostic Accuracy Study

ATHENA

ATHENA Participant Consent Form

Chief Investigator	Dr Konstantinos Balaskas
R&D Number	BALK1008
Site Name	<Insert local site name>
Principal Investigator	<Insert local PI name>

Please initial inside each box to indicate that you provide consent to participate in each part of the trial

1	I confirm that I have read and understand the participant information sheet (version__.__ dated __/__/____) for the ATHENA trial. I agree that I have had the opportunity to take time to consider my involvement in the trial and I have had the chance to ask questions, all of which have been answered to my satisfaction.	
2	I agree that my involvement in the ATHENA trial is voluntary, and I am free to withdraw at any time without the quality of my medical care or my legal rights being affected. I agree that if I decide to withdraw from the trial, any information that has already been analysed cannot be withdrawn. I understand that should I want to withdraw from the trial, then I will be contacted by a member of the trial team and given the options described in the ATHENA Participant Information Sheet about what other data can be collected from me and what happens to it, and that my response will be recorded on a withdrawal form.	
3	I agree that my hospital research team can provide a copy of my consent form, and relevant personal information, including my name, date of birth, and NHS number to the ATHENA trial office based at the University of Birmingham for use in the ATHENA trial.<br>I agree that my hospital research team can provide copies of my eye tests and a short description of my relevant medical history to the researchers based in Moorfields Eye Hospital for use in the ATHENA trial.	
4	I agree that relevant sections of my medical notes, and all copies of the tests from my eyes will be made available to members of the ATHENA research team based at specialist NHS Reading Centres (The Network of Ophthalmic Reading Centres - NetwORC UK) where they will be reviewed by specialist eye doctors.	

For peer review only - http://bmjopen.bmj.com/site/about/guidelines.xhtml

<Insert local trust header> Participant Trial Number: ☐☐☐☐☐☐

	I understand that my personal information including my name, date of birth, and NHS number will not be available to these doctors.	
5	I agree that copies of the results of the tests from my eyes, my unique trial ID number, and my age in months will be made available to members of the ATHENA research team based at Newcastle University, for review by experts for health economic analysis. I understand that my personal information including my name, date of birth, and NHS number will not be available to these researchers.	
6	I agree that my data will be anonymised and used in combination with that of others to produce research outputs such as reports, presentations, publications and websites connected to the ATHENA trial. I understand that I will not be individually identified in any publicly available output.	
7	I understand that all information collected from me for this trial will be subject to the General Data Protection Regulation and Data Protection Act 2018. This information will be stored securely by Moorfields Eye Hospital, which is the data controller for the ATHENA trial, for a minimum period of 10 years.	
8	I agree that collaborators of the ATHENA trial, and authorised representatives from the trial sponsor (Moorfields Eye Hospital), regulatory authorities and my NHS trust can access my data where relevant, such as my taking part in this research and for safety monitoring.	
9	I agree that my general practitioner (GP) can be informed of my participation in the ATHENA trial.	
10	I understand the information that has been given to me about the ATHENA trial and I agree to take part in this trial.	

Optional Consent Items

Please initial inside the boxes if you agree to the following optional consent items. Please note – the following items will not affect your eligibility to take part in the trial.

11	I agree that tests from my eyes are to be fully anonymised and can be used to teach eye specialists in the future.	
12	I agree that some anonymous information collected from me may be shared and/or made publicly available for other researchers to support other research in the future.	
13	I agree that I may be contacted by the research team in the future regarding further research that is linked to this trial.	

<Insert local trust header> Participant Trial Number: ☐☐☐☐☐☐

Name of Participant	
Signature	
Date	

Name of Person Taking Consent	
Signature	
Date	

Master copy for Site File, 1 copy for participant notes, 1 copy for Participant, 1 copy for ATHENA Trial Office

Patient Information Sheet

ATHENA Patient Information Sheet v2.0, 14-Jan-2022

< Insert local headed letterhead>

OpticAl Coherence TomograpHy Angiography for the dEtection of Neovascular Age-related Macular Degeneration: a Comprehensive Diagnostic Accuracy Study – the ATHENA Study

ATHENA

ATHENA Participant Information Sheet
Version 2.0, 14th January 2022

We would like to invite you to take part in the ATHENA trial. This is a research trial looking at the best way to confirm the presence of an eye condition known as neovascular age-related macular degeneration (nAMD), a common condition that affects the central part of your vision. We are especially interested in understanding the best way to look at the back of your eye to get a definite diagnosis.

It is important that you understand why this research is being done and what it will mean for you if you decide to take part. A member of our team will go through the trial with you and answer any questions that you might have. Please take your time to read the information carefully, and ask any questions, so that you can make an informed choice about taking part in the ATHENA trial.

If you have any questions or would like any further information about the ATHENA trial then please contact your local ATHENA research team using the details below:

Local PI: <Insert contact name>

Local PI email: <insert contact email>

Local Research Nurse: <Insert contact name>

Local Research Nurse email: <insert contact email>

IRAS No. 295260 ISRCTN18313457 R&D No. BALK1008

1

< Insert local headed letterhead>

Support and confidential advice can also be found through your hospital's NHS Patient Advisory Liaison Service (PALS) or equivalent:

Telephone: <Insert local PALS contact numbers> Email: <Insert local PALS email address>

Background of this trial

Age-related macular degeneration (AMD) is a fairly common condition that affects the light-sensitive cells at the back of your eye and can affect the central part of your vision. A more serious form of AMD called neovascular AMD (nAMD) can cause bleeds and swelling at the back of the eye leading to significant vision loss if left untreated. It usually affects people over the age of 55 and becomes more common with advancing age.

Eye specialists use several different devices to take images of the retina, a light-sensitive layer at the back of the eye which allows us to see. These images alone or in different combinations help eye specialists detect and diagnose nAMD. There are a number of ways of doing this, the most common being a test known as Optical Coherence Tomography (OCT), which is a quick, painless test using light to assess the back of your eye. Occasionally, OCT alone is not sufficient to confirm the diagnosis of nAMD with certainty. In these occasions, a second diagnostic test could be performed.

One such test is called Fundus Fluorescein Angiography (FFA) and has been used for many years:

- FFA uses a fluorescent dye (fluorescein) and a specialised camera for examining the circulation of the retina, which is in the back of the eye
- Fluorescein dye is injected into a vein in the arm, this is intravenous fluorescein angiography (IVFA)
- A number of pictures of the back of the eye are taken over several minutes and looked at by the eye specialist to help diagnose nAMD

Issues with current diagnostic process:

As well as being labour-intensive, time-consuming and inconvenient for the patient, FFA is not always informative. Sometimes a further dye test called Indocyanine-Green Angiography (ICGA) is required to confirm diagnosis of nAMD, especially when a form of nAMD called Polypoidal Choroidal Vasculopathy (PCV), is suspected.

Newer diagnostic tests are now available:

Another diagnostic test is called Optical Coherence Tomography-Angiography (OCTA). This is a more recent technology already available in many NHS Trusts. It is

For peer review only - http://bmjopen.bmj.com/site/about/guidelines.xhtml

quick, easy and painless and can provide information similar to FFA. Many eye units in the UK already use OCTA as their preferred first test to detect nAMD.

Purpose and brief overview of this trial

The purpose of this trial is to assess the different tests that are used to diagnose nAMD to see which tests, alone and in combination, provide the most accurate diagnosis.

We will recruit 1067 patients who have symptoms that suggest they have nAMD, from hospital eye units in the UK. In order to be eligible, patients will need to have the suspicion of nAMD confirmed by an OCT test, will need to be able to tolerate the tests used as part of the trial (OCTA, FFA, ICGA), and will need to provide informed consent.

Once recruited, participants will have both the OCTA and the FFA test (and ICGA test if there is suspicion of PCV). The images from the participant's tests will then be reviewed by their clinical care team and independent eye specialists, alone and in combination, to assess which tests provide the most accurate diagnosis of nAMD. For more detail, please see the section below, titled "What would taking part in the trial involve?".

Why have I been asked to take part?

Your hospital team have found that you may be able to take part in this trial if you want to. This means that you have had an OCT test and your clinical team think that you are likely to have nAMD in at least one of your eyes. We would like you to help us to understand which are the best combinations of imaging tests to use to diagnose nAMD in other people in the future.

Do I have to take part?

No. Taking part in this trial is entirely voluntary, so it is up to you if you want to take part or not. If you don't want to take part you do not have to give us a reason why. Please rest assured that no matter what you decide, your health care team will always work in your best interests and the quality of your care will not be affected in any way.

What would taking part in the trial involve?

You will have already had an OCT test that shows suspicion that you may have nAMD. If you take part in this trial you will be asked to have two further tests: an OCTA test and an FFA test (and the ICGA test if there is suspicion of PCV). If you decide not to take part in ATHENA then it is likely that you will have at least one, if not both, of these other tests to confirm your diagnosis.

We will ask the eye specialists who are looking after you to look at the tests and decide whether you have nAMD and how confident they are of this diagnosis. In half of the cases, chosen by chance, the eye specialists will base their first diagnosis on the OCT and OCTA

< Insert local headed letterhead>

tests, and in the other half of cases the eye specialist will base their diagnosis on the OCT and FFA tests.

The eye specialists will then be asked to look at the third test to confirm or change their initial diagnosis. We will compare the results of both combinations of tests to see if they are equally as good for detecting nAMD.

If you do decide to take part, and give your permission, we would like to transfer all the images taken in the diagnosis of your nAMD alongside a brief description of your relevant medical history to members of the ATHENA research team based at specialist NHS Reading Centres (The Network of Ophthalmic Reading Centres - NetwORC UK) in the UK. The ATHENA research team at the NetwORK UK centres are eye specialists (expert image graders). These specialists will review the images and medical history, and will make a diagnosis on these independently to your usual eye doctors. The results from the specialist reading centres will not be made available to your doctors and they will not change the care you will receive from your doctors.

If you give your permission, we would also like to transfer the results of your eye tests, any adverse events that occur whilst participating in the trial, information on any relevant medication that you may be taking, and your age in years, to experts at Newcastle University for health economic analysis. This will allow the ATHENA team at Newcastle University to assess how cost-effective the tests are.

By comparing the results of the combination of tests reviewed by your eye care team, and by independent eye experts, we will be able to decide how good each of these tests is at detecting nAMD.

Are there any benefits for me taking part in the trial?

This trial will help us to determine the best combination of tests to use to diagnose nAMD, and you will have all three of the commonly used tests (normally you would only have two tests). All three tests will be available to your treating doctor for them to make a diagnosis. Whilst there may be no other immediate benefit to you, the information that you provide to us will help in the long-term to improve the diagnosis of nAMD in the future.

Are there any risks in taking part?

All procedures have some element of risk associated with them, however low. The main risk in ATHENA is that the dye used in FFA imaging may make you feel unwell, and a very small number of people (1:100,000) may be allergic to it. However, as you have already been referred with suspected nAMD it is likely that you would undergo this procedure anyway unless you knew you were allergic to dyes, thus taking part in this trial is not associated with any additional risk above that which you would get with your usual care.

Will my GP know about this?

ATHENA Patient Information Sheet v2.0, 14-Jan-2022

< Insert local headed letterhead>

Yes, and with your permission, your GP practice will be notified about your participation in the ATHENA trial. They will be given details about how they access more information about the trial.

Who is organising and funding this research?

This important research is being funded by the National Institute for Health Research (NIHR) Health Technology Assessment (HTA) programme (reference number: NIHR131432). This is UK government's largest funder of healthcare research. This trial is sponsored by Moorfields Eye Hospital NHS Foundation Trust who have reviewed the research plans and have accepted legal and ethical responsibilities for the trial (reference number: BALK1008). The research will be run by the University of Birmingham Clinical Trials Unit (BCTU) which is a specialist department that plans, organises and conducts clinical trials such as ATHENA.

Has this trial been reviewed and approved?

Any research that is conducted within the NHS must follow the strict guidelines and regulations that are in place to ensure patient safety. The South Central - Oxford B Research Ethics Committee (REC) are an independent group that have approved this trial after ensuring that your safety, rights, well-being and dignity will not be compromised if you decide to take part. In addition, the research & development (R&D) department of each hospital involved in this trial has reviewed the ATHENA trial and have approved it to be carried out within their hospital.

Have any patients or members of the public been involved in this trial?

When designing this trial, it was important for us to ensure that the comments and suggestions of the public are taken on board, so that we could ensure that that this trial was suitable for people with suspected nAMD. To do this, this trial was developed with the help of the people with suspected nAMD in Belfast and London who agreed that they would welcome a non-invasive test to replace FFA and help clinicians confirm the diagnosis of nAMD more quickly.

Representatives of people who have undergone the tests are involved in the group who oversee the management of the ATHENA trial.

What personal information will you be collecting?

In order to carry out this research project we will need to collect information about you and some of this information will be personal data. Under the data protection law, we have to provide you with very specific information about what we do with your data and about your rights. We have set out below the key information you need to know about how we will use your personal data.

ATHENA Patient Information Sheet v2.0, 14-Jan-2022

< Insert local headed letterhead>

More information on how the University of Birmingham processes personal data can be found on the University's website on the page called 'Data Protection - How the University Uses Your Data' (https:// www.birmingham.ac.uk/privacy/index.aspx).

Personal information we will collect includes your name, biological sex, NHS number, date of birth, copies of your eye tests and a short description of your relevant medical history.

Who is the Data Controller?

Moorfields Eye Hospital NHS Foundation Trust is the data controller for the personal data that we process in relation to you.

What data are we processing and for what purpose will we use it?

We will collect and process your personal data to conduct the research project as explained in this Participant Information Sheet.

What is our legal basis for processing your data?

The legal justification we have under data protection law for processing your data is that the sponsor is undertaking medical research in the public interest. We do not envisage any problems occurring as a result of your participation in the trial. However, all patients are covered for negligent harm according to NHS indemnity guidelines. If you have a concern about any aspect of this trial, you should ask to speak to a member of the research team who will do their best to answer your questions (contact details are on the front cover of this information leaflet).

How will we use information about you?

We will need to use information from you and your medical records for this research project.

People will use this information to do the research or to check your records to make sure that the research is being done properly.

Once we have finished the trial, we will keep some of the data so we can check the results. With your permission we would like to fully anonymise your tests and use them to teach eye specialists in the future. We will write our reports in a way which makes sure that no-one can work out that you took part in the trial.

Who will my personal data be shared with?

The personal data that we will collect about you will include your name, date of birth, biological sex, NHS number, and a short description of your relevant medical history. The only people who will see this information are the people who look after you at your hospital, a very small number of the trial team at the University of Birmingham who are directly managing the ATHENA trial, and any representatives from regulatory bodies who

ATHENA Patient Information Sheet v2.0, 14-Jan-2022

< Insert local headed letterhead>

need to ensure that the research is being done properly. Relevant aspects of your data will be provided by your clinical care team to the ATHENA trial team based at the University of Birmingham and Newcastle University as necessary, via specifically designed databases. Only your unique trial number will be used as an identifier in any correspondence between your clinical care team and the other institutions where the ATHENA trial team are based.

The trial team at the University of Birmingham will store this information on a secure database hosted at the University of Birmingham which will produce a unique trial number, which they will give to your hospital. This trial number will be used as a link to identify your information for the rest of the trial team.

Staff at your treating hospital will produce copies of your tests that have all your personal information removed and replaced by the unique trial number, which acts as a linker. These tests plus some relevant medical information and your treating clinician's diagnosis will be uploaded onto a computer database based at Moorfields Eye Hospital NHS Foundation Trust.

Using a secure portal, NHS staff at the reading centres will be shown the image alongside the relevant medical information and asked to make a diagnosis from this. This diagnosis from the reading centre will be recorded on the database hosted at Moorfields Eye Hospital NHS Foundation Trust.

The results of the tests from your hospital and the NHS reading centre will be sent back to the University of Birmingham, where the ability of these tests to successfully diagnose nAMD will be looked at. People doing this analysis will not have access to your name.

At the end of the trial your unique trial number, age in months and the diagnosis from the tests will be transferred to Newcastle University who will look at the value for money that each of the tests provides. People doing this analysis will not have access to your name or full date of birth.

We will keep the identifiable information about you that is collected from this trial for a minimum of 10 years after the trial has finished. The identifiable data we hold about you will not be shared with any third party during this storage period.

When you agree to take part in a research trial, the anonymised information about your health and care may be provided to researchers who may be based in universities, NHS organisations or companies involved in health and care research in this country or abroad. Your anonymised information will only be used by organisations and researchers to conduct research in accordance with the UK Policy Framework for Health and Social Care Research. If this information is shared it will not identify you and will not be combined with other information in a way that could identify you. The information will only be used for the purpose of health and care research and cannot be used to contact you or to affect your care. It will not be used to make decisions about future services available to you (e.g. insurance).

ATHENA Patient Information Sheet v2.0, 14-Jan-2022

< Insert local headed letterhead>

How will my personal data be kept secure?

All data will be handled in accordance with the General Data Protection Regulations (GDPR) and Data Protection Act 2018.

All Moorfields Eye Hospital NHS Foundation Trust clinical trial databases are stored in secure, monitored servers. All Moorfields Eye Hospital NHS Foundation Trust clinical trial databases are part of the Moorfields Eye Hospital NHS Foundation Trust disaster recovery strategy and have a 5-day Recovery Time Objective.

Where data is transferred electronically to Moorfields Eye Hospital NHS Foundation Trust this will be in accordance with the General Data protection Regulations (GDPR) and UK Data Protection Act 2018 as well as Moorfields Eye Hospital NHS Foundation Trust Information Security Policy and Trust Information Governance Policy. There will be a documented record of any data transfer regarding the trial management.

Access to study data stored in Moorfields Eye Hospital NHS Foundation Trust servers will be granted to authorised representatives from the Sponsor, host institution and the regulatory authorities to permit trial-related monitoring, audits and inspections- in line with participant consent.

The University of Birmingham ensures that personal data is handled, stored and disposed of confidentially and securely. Staff receive regular data protection training, and the University has put in place organisational and technical measures so that personal data is processed in accordance with the data protection principles set out in data protection law.

The University of Birmingham has an Information Security Management System based on ISO27001 with a range of controls covering the protection of personal information. Annual security awareness training is mandatory for staff and the University is accredited under the NHS Information Governance Toolkit, the Payment Card Industry Data Security Standard and is in the process of gaining Cyber Essentials Plus for defined services. In relation to this project, electronic data will be kept on secure, encrypted IT servers within the University of Birmingham. Any physical paperwork containing identifiable data will be kept inside a locked filing cabinet, in an access-controlled room within an access-controlled building.

How long will my personal data be kept?

Your data will be retained for a minimum of 10 years after the publication of the research outcomes. If you withdraw from the project, we will keep the information we have already obtained and processed but, to safeguard your rights, we will collect the minimum amount of information that personally identifies you.

Your rights in relation to your data

Your rights to access, change or move your information are limited, as we need to manage your information in certain ways in order for the research to be reliable and accurate. If you withdraw from the project, we will keep the information we have already obtained but, to

ATHENA Patient Information Sheet v2.0, 14-Jan-2022

< Insert local headed letterhead>

safeguard your rights, we will use the minimum amount of personally-identifiable information that we are able to.

You can withdraw your consent to our processing of your data at any time. We will store the information about you that we have already obtained if this has already been analysed, but we will clarify with you whether we can use your stored data, if at all, for any new analysis.

In the unlikely event that you lose the capacity to consent during the trial you will be withdrawn, and we will use any data already collected.

Should you wish to withdraw from the trial, but do not let the trial staff or trial office know of the type of withdrawal you want, then we will assume that you are happy for us to continue using the data we have collected on you and we will also assume that you do not want the trial office to contact you in the future.

If you decide to withdraw and let us know that you do not want us to use your data then no new analysis will be started using your information. However, we cannot remove your data from any analysis that was completed prior to your withdrawal from the trial.

Under the provisions of the General Data Protection Regulation (GDPR) 2018, you have the right to know what information the Trials Office has recorded about you. If you wish to view this information, or find more about how we use this information, please contact Legal Services at the address below. Please note that a small fee may be payable to retrieve this information.

If you would like more information on your rights, would like to exercise any right or have any queries relating to our processing of your personal data, or if you wish to make a complaint about how your data is being or has been processed, please contact:

Moorfields Data Protection Officer: Jo Downing, Head of Information Governance, Moorfields Eye Hospital Email: Moorfields.ig@nhs.net

The Information Compliance Manager, Legal Services, The University of Birmingham, Edgbaston, Birmingham B15 2TT
Email: dataprotection@contacts.bham.ac.uk
Telephone: +44 (0)121 414 3916

What will you do with the results of the research trial?

The results of the ATHENA trial will be made available in specialist medical journals, on publicly available websites, and will be presented at conferences. This will help doctors, patients, and key decision makers within the NHS to get a better understanding of which type of diagnostic test is most useful and is the best for nAMD patients. If we do publish the results of this trial, please rest assured that any information relating to you will be completely anonymous and there will be no way that you can be identified. If you would like to know the final results of this trial, please let a member of the research team know and we will be more than happy to provide you with a summary of the research once it has finished.

ATHENA Patient Information Sheet v2.0, 14-Jan-2022

< Insert local headed letterhead>

Do you have any further questions?

Please take the time to decide whether you wish to take part in the ATHENA trial. Please feel free to discuss this invitation to join the ATHENA trial with your friends, relatives, or anybody else that you want to. It is however likely that you will be asked to make a decision on whether you take part in this study on the same day as when the trial is first discussed with you. This is because nAMD is an urgent condition and the diagnosis needs to be confirmed on the day of your referral visit so that we can start vision-saving treatment as soon as we can.

Having read this information sheet, we hope that you will choose to take part in the ATHENA trial. If you have any questions about the trial now or later, feel free to ask your medical team, or the person who is responsible for the ATHENA trial at your hospital. Their name and telephone number are shown at the start of this document. Only the clinical staff looking after you can give you advice about your medical care and the treatment options that may be available to you.

If you require any general information about research, the UK Clinical Research Collaboration has produced a useful guide entitled, 'Understanding Clinical Studies'. This can be downloaded from their website: www.ukcrn.org.uk. If you require specific information about the research project, please either contact any of the ATHENA staff listed at the start of the document or visit our website: www.birmingham.ac.uk/ATHENA.

For peer review only - http://bmjopen.bmj.com/site/about/guidelines.xhtml

BMJ Open

Study Protocol: Optical Coherence Tomography Angiography for the detection of Neovascular Age-related Macular Degeneration: a Comprehensive Diagnostic Accuracy Study - the ATHENA study

Journal:	*BMJ Open*
Manuscript ID	bmjopen-2022-070857.R1
Article Type:	Protocol
Date Submitted by the Author:	15-May-2023
Complete List of Authors:	Niestrata, Magdalena; Moorfields Eye Hospital City Road Campus, Ophthalmology<br>Deeks, Jonathan; University of Birmingham, Public Health, Epidemiology and Biostatistics<br>Takwoingi, Yemisi; University of Birmingham, Public Health and Epidemiology<br>Sivaprasad, Sobha; Moorfields Eye Hospital City Road Campus<br>Patel, Praveen; Moorfields Eye Hospital NHS Foundation Trust, NIHR Biomedical Research Centre<br>Keane, Pearse; Moorfields Eye Hospital NHS Foundation Trust<br>Kernohan, Ashleigh; Newcastle University<br>Vale, Luke; Newcastle University<br>Denniston, Alastair; Queen Elizabeth Hospital Birmingham, UK<br>Gale, Richard; York Teaching Hospital NHS Foundation Trust<br>Khan, Adam R.; University of Birmingham<br>McKinnon, William; University of Birmingham<br>Agarwal, Ridhi; University of Birmingham, Test Evaluation Research Group, Institute of Applied Health Research,<br>de Salvo, Gabriella; Southampton University Hospitals NHS Trust<br>Minos, Evangelos; North West Anglia NHS Foundation Trust, Ophthalmology<br>Barbeiro, Paulo; BlueWorks - United Kingdom<br>Chakravarthy, Usha; Queen's University Belfast, Ophthalmology<br>Waheed, Nadia K; New England Eye Center<br>Madhusudhan, Savita; Liverpool University Hospitals NHS Foundation Trust<br>Peto, Tunde; Queen's University Belfast Faculty of Medicine Health and Life Sciences<br>Balaskas, Konstantinos; NIHR Moorfields Biomedical Research Centre, NHS Foundation Trust and UCL Institute of Ophthalmology
**Primary Subject Heading**:	Ophthalmology
Secondary Subject Heading:	Ophthalmology
Keywords:	Ophthalmology < SURGERY, OPHTHALMOLOGY, Medical retina < OPHTHALMOLOGY

SCHOLARONE™
Manuscripts

Study Protocol: Optical Coherence Tomography Angiography for the detection of Neovascular Age-related Macular Degeneration: a Comprehensive Diagnostic Accuracy Study - the ATHENA study

Magdalena Niestrata [1], Jon Deeks [2], Yemisi Takwoingi [2], Sobha Sivaprasad [1], Praveen J. Patel [1], Pearse A. Keane [1], Ashleigh Kernohan [3], Luke Vale [3], Alastair K. Denniston [4], Richard Gale [5], Adam R. Khan [6], William McKinnon [6], Ridhi Agarwal [2], Gabriella de Salvo [7], Evangelos Minos [8], Paulo Barbeiro [9], Usha Chakravarthy [10], Nadia Waheed [11], Savita Madhusudhan [12], Tunde Peto [10], Konstantinos Balaskas [1]

1. NIHR Biomedical Research Centre at Moorfields Eye Hospital NHS Foundation Trust and UCL Institute of Ophthalmology, London, UK
2. Institute of Applied Health Research, University of Birmingham, Birmingham, UK
3. Health Economics Group, Population Health Sciences Institute, Newcastle University, Newcastle, UK
4. University Hospitals Birmingham NHS Foundation Trust, Birmingham, UK
5. York Teaching Hospital NHS Foundation Trust, York, UK
6. Birmingham Clinical Trials Unit, University of Birmingham, Birmingham, UK
7. Southampton University Hospital NHS Foundation Trust, Southampton, UK
8. North West Anglia NHS Foundation Trust, Hinchingbrooke and Peterborough, UK
9. Blueworks Medical Expert Diagnosis, Lisbon, Portugal
10. Queen's University Belfast, Belfast, UK
11. New England Eye Centre, Boston, USA
12. Liverpool University Hospitals NHS Foundation Trust, Liverpool, UK

Corresponding author: Konstantinos Balaskas, Moorfields Reading Centre and Clinical AI Lab, Moorfields Eye Hospital NHS Foundation Trust, 162 City Rd, London EC1V 2PD, k.balaskas@nhs.net

Wordcount: 3692

MeSH terms:

- Diagnostic Techniques, Ophthalmological
- Humans
- Reproducibility of Results
- Sensitivity and Specificity
- Tomography, Optical Coherence / methods
- Wet Macular Degeneration / diagnosis

For peer review only

Abstract

Introduction

The diagnosis of neovascular Age-related Macular Degeneration (nAMD), the leading cause of visual impairment in the developed world, relies on the interpretation of various imaging tests of the retina. These include invasive angiographic methods, such as Fundus Fluorescein Angiography (FFA) and, on occasion, Indocyanine-Green Angiography (ICGA). Newer, non-invasive imaging modalities, predominately Optical Coherence Tomography (OCT) and Optical Coherence Tomography Angiography (OCTA), have drastically transformed the diagnostic approach to nAMD. The aim of this study is to undertake a comprehensive diagnostic accuracy assessment of the various imaging modalities used in clinical practice for the diagnosis of nAMD (OCT, OCTA, FFA and, when a variant of nAMD called Polypoidal Choroidal Vasculopathy is suspected, ICGA) both alone and in various combinations.

Methods and analysis

This is a non-inferiority, prospective, randomised diagnostic accuracy study of 1067 Participants. Participants are patients with clinical features consistent with nAMD who present to a National Health Service (NHS) secondary care ophthalmology unit in the UK. Patients will undergo OCT as per standard practice and those with suspicious features of nAMD on OCT will be approached for participation in the study. Patients who agree to take part will also undergo both OCTA and FFA (and ICGA if indicated). Interpretation of the imaging tests will be undertaken by clinicians at recruitment sites. A randomised design was selected to avoid bias from consecutive review of all imaging tests by the same clinician. The primary outcome of the study will be the difference in sensitivity and specificity between OCT+OCTA and OCT+FFA (+/- ICGA) for nAMD detection as interpreted by clinicians at recruitment sites.

Ethics and dissemination

The study has been approved by the South Central - Oxford B Research Ethics Committee with reference number 21/SC/0412.

Dissemination of study results will involve peer-review publications, presentations at major national and international scientific conferences.

Strengths and limitations

Strengths

- Large multicentre comprehensive diagnostic accuracy study of all imaging modalities in common clinical use for nAMD diagnosis.
- The study design maximises its applicability to NHS clinical practice because test interpretation is made by clinicians at recruitment sites and it has an embedded economic evaluation.

For peer review only

- All imaging tests are transferred, randomly and after pseudonymisation, to a laboratory environment for unbiased review and determination of diagnostic accuracy of different imaging tests in isolation.

Limitations

- The study design is complex because randomisation for sequence of image review is required to prevent bias from consecutive interpretation of all imaging tests for each case by the same clinician at recruitment sites.
- The eligibility criterion of presence of nAMD suspicious features on OCT permits the assessment of Positive Predictive Value only for OCT but this study design ensures the feasibility of the recruitment plan.

For peer review only

Background and Study Aims

Age-related macular degeneration (AMD) is the most frequent cause of severe visual impairment and blindness in elderly populations in the developed world including the UK, Europe, the United States and Australia. With age being the strongest risk factor for the development of AMD, the numbers of affected patients are steadily increasing in view of the aging population. [1] Consequently, the incidence of visual impairment and blindness caused by AMD has significantly increased from 1990 to 2010. [2] Worldwide, the number of patients affected by AMD is estimated to increase to 288 million in 2040. [3] [4]

AMD is categorised into non-neovascular or dry and neovascular or wet, based on the absence or presence of macular neovascularisation (MNV) [5] Neovascular AMD (nAMD) is characterised by fluid leakage or bleeding from new permeable vessels originating from the choroidal or deep retinal capillary network. MNV can be subclassified into type 1, otherwise known as occult, type 2, or classic and type 3 or retinal angiomatous proliferation (RAP), depending on the location of the MNV - beneath the retinal pigment epithelium (RPE), within the subretinal space and intraretinally, respectively. [6] [7]

In nAMD regardless of MNV subtype, treatment with anti-vascular endothelial growth factor (VEGF) therapy has been shown to be effective.[13-17] In those cases, early diagnosis and treatment is associated with improved visual prognosis and, improved patient quality of life and reduced morbidity. [8]

The diagnosis relies on imaging techniques visualising the retina and choroidal circulation. Optical Coherence Tomography (OCT), which has revolutionised the diagnosis and management of numerous retinal pathologies, features of nAMD disease activity including subretinal and intraretinal fluid, however, cannot directly detect a CNV. Until recently, FFA has been the gold-standard for the diagnosis of nAMD as it allows for visualisation of leakage from MNV. [4]

Nonetheless, this procedure is associated with the risk of complications, such as discoloration of urine, nausea and vomiting, and carries an extremely low risk of anaphylactic reaction. It is also contraindicated in kidney failure, which is not uncommon in elderly patients with systemic comorbidities who are investigated for nAMD. [9] The procedure is invasive, requiring intravenous cannulation and unpleasant for patients. In

addition, the acquisition of images from FFA is time and labour-consuming, leading to significant difficulty due to limited resources and increasing numbers of patients requiring diagnostics. [10] Finally, occasionally FFA alone is not sufficient and additional imaging, such as Indocyanine-Green Angiography (ICGA) is required, particularly when Polypoidal Choroidal Vasculopathy (PCV), a variant of nAMD, is suspected.

ICGA has long been considered as the standard for PCV diagnosis and is generally considered as essential to make a definitive diagnosis of PCV. [11] Other imaging modalities have been assessed for the detection of PCV. [12] Fukuyama et al evaluated 62 polypoidal lesions detected on ICGA and 79% were detected on OCTA. [13] In a study which evaluated the SD-OCT findings of 188 eyes with PCV or neovascular AMD, results suggested that SD-OCT had a 89% sensitivity and 85% specificity in identifying PCV when 2 of the 3 signs were present: PED, double-layer sign, and thumb-like polyp. [14]

The advent of new non-invasive imaging techniques based on OCT, such as Optical Coherence Tomography Angiography (OCTA) has enabled a rapid direct visualisation of MNV without the need for intravenous contrast and without the side effects associated with FFA.

Faes and colleagues conducted a systematic review and meta-analysis of the performance of OCTA in detecting retinal disease. Over 1600 research articles involving OCTA have been published but only 17 studies were diagnostic accuracy studies. None of the studies were prospective and they provided scarce data on patient selection, data collection and analysis, masking, descriptions of the OCTA results and the reference standard. Out of the 17 studies, only 5 small studies evaluated detection of nAMD. These reported a wide range of sensitivity (0.50 to 1.00) and specificity (0.68 to 1.00), reinforcing the need for a well-designed diagnostic accuracy study to inform NHS decision-making on the role of OCTA. [15]

Cicinelli and colleagues (2020) have shown the overall sensitivity of OCTA for detection of active CNV to be 80.7% [16] In addition, the OCTA images have been shown to be less affected by subretinal haemorrhage compared to FFA. [17] [18] In terms of PCV detection, Cheung and colleagues showed a combination of structural OCT and OCTA achieved 82.6% sensitivity and 100% specificity [19] As a result, RCOphth guidance and some countries, such as France, have already changed their guidelines advocating increasing use of OCTA instead of FFA for nAMD diagnosis [20] However, there are no prospective, statistically powered, diagnostic accuracy studies that could inform the optimal diagnostic imaging protocol for nAMD.

In addition to the clinical implications of the performance of OCTA compared with the other imaging strategies there may be significant resource implications associated with the different strategies. As such it is important to understand the relationship between any additional costs associated with certain imaging strategies and any benefits that they may provide. Alongside this diagnostic accuracy study, an economic decision modelling study will be used to assess the cost effectiveness of different strategies.

Due to the lack of high quality evidence, there is variability in clinical practice with respect to the optimal imaging protocol for nAMD diagnosis. The original clinical interest in OCTA as a potential replacement of FFA in the diagnosis of nAMD has been superseded by the

For peer review only

understanding that both techniques have relative and, on occasion, complementary merits, yet their optimal use for nAMD diagnosis remains unclear [15] The aim of ATHENA is to address these clinically relevant research questions.

There are also significant resource implications associated with the different strategies. Therefore, to guide decision-making the value for money of the different imaging strategies will be estimated.

Methods

Study Design

The ATHENA study is a non-inferiority, prospective, randomised diagnostic accuracy study with embedded economic evaluation and an internal pilot to confirm feasibility of the recruitment plan. The study started on the 1st March 2022, with a planned end date of 31st December 2024.

Patient and Public Involvement

There was extensive patient and public involvement, as detailed in the supplementary elements.

Patient Population

Participants with a suspicion of nAMD in the first or second eye on OCT that present to an NHS secondary care ophthalmology unit in the UK will be recruited. They will be randomised to either OCT + FFA or OCT + OCTA study arms (**Figure 1**).

Study Procedures

In addition to OCT, each patient will undergo both OCTA and FFA. Patients with clinical or OCT appearance suggestive of PCV will also undergo ICGA at the same time as FFA (**Figure 1**).

In half the cases, selected randomly, an expert clinician at the recruitment site will review the OCT and then the OCTA and provide a clinical diagnosis (nAMD: yes/no). In the other half of the cases the site clinician will review the OCT and then the FFA and provide a clinical diagnosis (nAMD: yes/no) (CD1). (**Figure 1**) Subsequently, the same clinician will review the remaining imaging test in each case (FFA or OCTA) and will either confirm or change their initial clinical diagnosis (nAMD: yes/no) – recorded as clinical diagnosis 2 (CD2). (**Figure 1**) Comparison of the two clinical diagnoses within each arm will provide additional evidence concerning the added value of using a combination of both OCTA and FFA in patients with an OCT suggestive of nAMD.

Figure 1. Study design

Setting

Recruitment will take place in 20 hospital-based eye units across the UK. Of those 6 will be involved in the pilot study and all sites will be involved in the main study. The study

For peer review only

coordinating centre (Trial Office) is the Birmingham Clinical Trials Unit (BCTU), based at the University of Birmingham.

Participants will be recruited amongst patients referred by their community optometrist, GP, or self-referred to secondary care eye casualty departments with a suspicion of nAMD and amongst patients already under the care of NHS eye units for an established diagnosis of nAMD in one eye who develop symptoms of it in the fellow eye. Using Eligibility criteria to define inclusion and exclusion criteria for potential patients **(Table 1).**

Eligibility Criteria

Table 1

Inclusion Criteria:	Exclusion Criteria:
Patients with suspicion of nAMD in the first or second eye who have:	Significant media opacities (cataract, vitreous opacities) that would not allow good quality fundus imaging;
Signed informed consent form;	Diabetic retinopathy of severity worse than mild non-proliferative stage and with any degree of diabetic maculopathy;
Ability to perform study specific procedures.	Other causes of choroidal neovascularisation (myopic, angioid streaks, inflammatory, retinal dystrophies, secondary to central serous chorioretinopathy, idiopathic);
	Inability to undergo dye-based imaging (FFA or ICGA) due to history of allergy.

Randomisation and Blinding

The purpose of randomisation in this study is to minimise bias in the comparative accuracy of OCT+OCTA and OCT+FFA (primary objective) that may arise from non-masked interpretation of the findings of OCTA and FFA by the same clinician. Randomisation will be provided by a secure online randomisation system at BCTU (http://www.trials.bham.ac.uk/athena), which will be available 24 hours a day, 7 days a week, apart from short periods of scheduled maintenance. The system will randomise each patient to one of the two study arms as described in study design (**Figure 1**).

A minimisation algorithm will be used within the online randomisation system to ensure balance in the treatment allocation over the following variables: the recruiting centre, whether the affected eye is the first or second eye and whether the case is suspicious of PCV. A ‘random element’ will be included in the minimisation algorithm, so that each patient has a probability of being randomised to the opposite study arm than they would otherwise be assigned to.

Sample size calculation

According to current literature, the sensitivity of OCT + OCTA and OCT + FFA for detection of nAMD is 92% [15] [21] [22] and 90% [23], respectively. Based on the above, 240 patients with nAMD will need to be recruited to ensure, with 90% certainty, that the upper limit of a

For peer review only - http://bmjopen.bmj.com/site/about/guidelines.xhtml

one-sided 97.5% confidence interval (or equivalently a 95% two-sided confidence interval) will exclude a difference in favour of OCT+FFA of more than 10 percentage points (i.e. the non-inferiority margin). A non-inferiority margin of 10 percentage points was deemed appropriate in view of the expected advantages of OCTA (i.e. improved patient experience through the use of a quick non-invasive imaging technique) and an already established role in clinical practice.

Audit data from Moorfields Eye Hospital indicated a prevalence of 75% of confirmed nAMD in patients with a positive or suspicious OCT, based on which, 320 patients with suspected nAMD will be required. However, as the aim of the study is to compare specificity as well as sensitivity, using the same estimates as for sensitivity, it is anticipated that 960 patients will be required. Allowing for 10% withdrawal rate, missing data and inconclusive test results, we would aim to recruit a total of 1,067 patients.

Objectives - Outcome Measures and their Analysis

The primary analysis, against the objectives **(Table 2)**, will be based on a pragmatic approach to image interpretation for nAMD detection by clinicians at recruitment sites (comparison of OCT+OCTA versus OCT+FFA in patients with positive or suspicious OCT). This approach reflects real-life clinical practice and will allow a meaningful assessment of the diagnostic accuracy of the most commonly used confirmatory tests for nAMD detection in a clinical setting. This approach will enhance the potential for generalisability of study results in the NHS.

Figure 2: Methodological flowchart

All images (OCT, OCTA, FA and where available ICGA) will also be transferred to NetwORC UK Reading Centres Network using a secure web-based image management system. The accuracy of the individual imaging tests in isolation (OCT, OCTA, FA) will be assessed in a masked and random fashion by expert graders in the controlled environment of the Reading Centres. This will ensure the masked assessment of individual imaging tests independent of each other with the use of a sophisticated grading queue that ensures random allocation of tests and minimises bias. Grading of the images will be performed at the three Reading Centres by trained graders who have achieved a minimum of 90% agreement in the accreditation test, as per figure 2 above which highlights the unique methodology that combines an RCT (CONSORT diagram) with a Diagnostic Accuracy study (STARD diagram). The allocation of images will take into account the study design so that graders involved in grading of the reference standard will not be involved in grading the index tests. Dedicated image management software will be used for the purposes of aggregating imaging data from recruitment sites and developing grading queues for Reading Centre graders. The imaging tests will be presented in random order to the graders to avoid bias associated with grading images from the same patient in sequence. Graders will be masked to all clinical information as well as whether the imaging tests being reviewed originate from the same patient or not.

For peer review only

Objectives

Table 2

Primary Objective		Primary Outcome Measure
**The main aim of this study is to assess whether the sensitivity and specificity of OCTA combined with OCT is non-inferior to that of FFA combined with OCT, for the detection of neovascular age-related macular degeneration, in patients with clinical and OCT features suggestive of the diagnosis; as interpreted by clinicians.**		The primary outcome measure is the difference in sensitivity and specificity between OCTA combined with OCT and FFA combined with OCT. The unit of analysis is the patient (one 'trial' eye per patient). The sensitivity, specificity, PPV and NPV of the tests will be calculated. The diagnostic accuracy of OCT+OCTA and OCT+FFA will be compared to assess non-inferiority. The differences in sensitivity and specificity will be established and two-sided 95% confidence intervals calculated using the Wilson CI score method. If the lower boundary of the two-sided 95% confidence interval (CI) for the difference in sensitivity and specificity of OCT+OCTA relative to OCT+FFA is higher than −10 percentage points, the sensitivity of OCT+OCTA will be considered as non-inferior

review only

Secondary Objectives	Secondary Outcome Measures
**1. To assess the diagnostic accuracy of OCTA alone and FFA alone as reviewed by Reading Centre expert graders for the detection of nAMD in patients with a positive or suspicious OCT.**	Sensitivity, specificity, PPV and NPV of OCTA and FFA in isolation will be assessed on the basis of masked review by expert graders in the Reading Centres; 95% confidence intervals will be calculated using the Wilson method.
**2. To assess the positive predictive value (PPV) of OCT for the detection of nAMD in all patients presenting with suspicion of nAMD.**	The PPV of OCT for nAMD detection and its 95% CI will be estimated. Since only patients with a positive or suspicious OCT will be included, the sensitivity, specificity and NPV of OCT alone cannot be assessed.

**3. To compare the diagnostic accuracy of the combination of OCT+FFA versus OCT+FFA+OCTA as interpreted by retinal experts within the 'OCT+FFA' arm of the study.**	In order to establish the added diagnostic value of using a third imaging technique for nAMD detection, OCT+FFA will be compared against OCT+FFA+OCTA, in terms of differences in sensitivity and specificity together with their 95% Cis.
**4. To compare the diagnostic accuracy of the combination of OCT+OCTA versus OCT+FFA+OCTA as interpreted by retinal experts for the detection of nAMD within the 'OCT+OCTA' arm of the study.**	In order to establish the added diagnostic value of using a third imaging technique for nAMD detection, OCT+OCTA will be compared against OCT+OCTA+FFA, in terms of differences in sensitivity and specificity together with their 95% Cis.
**5. For a subset of cases with OCT and clinical features suspicious of Polypoidal Choroidal Vasculopathy (PCV) that underwent Indocyanine-Green Angiogrpahy (ICGA), to assess diagnostic accuracy of OCTA, FFA, ICGA, alone and in combinations, for the detection of PCV.**	For a subset of cases with clinical and OCT features suspicious of PCV, the diagnostic accuracy of OCTA, FFA and ICGA alone and in combination will be assessed by calculating their sensitivity, specificity, PPV and NPV (including their 95% CIs).
**6. To assess intra- and inter-rater agreement in the detection of nAMD on OCTA and FFA as assessed by Reading Centre graders.**	For OCTA and FFA, intra- and inter-rater agreement will be determined using percentage agreement and Gwet's first-order agreement coefficient (Gwet's AC1). [24] [25]
**7. To develop and validate criteria for the OCTA-based diagnosis of nAMD.**	The framework for OCTA-based nAMD diagnostic criteria created through this study will be assessed against a reference standard from the Reading Centres using all available multi-modal imaging (OCT, OCTA, FFA and ICGA where available) enhanced with clinical vignette describing patient symptoms and follow-up clinical data.
**8. To estimate the incremental cost per true positive detected and incremental cost per correct diagnosis for nAMD through a model based cost-effectiveness analysis.**	A model-based cost-effectiveness analyses (CEA) will be conducted to estimate the incremental cost per true positive detected and incremental cost per correct diagnosis for nAMD.
**9. To assess the diagnostic accuracy of OCTA by lesion type (PCV, type 1-, type 2-, type 3- nAMD).**	Sensitivity, specificity, PPV and NPV (including their 95% CIs) of OCTA for detection on nAMD by lesion type (PCV, type 1-, type 2-, type 3- nAMD) will be computed based on assessment by expert graders in the Reading Centres.

**10. To report limitations of OCTA use and adverse events.**	Number and classification of adverse events

Reference Standard

To provide the reference standard we will use all available imaging information from multi-modal imaging (OCT, OCTA, FFA and ICGA where available) enhanced with clinical vignette describing patient symptoms and available follow-up clinical data. The images will be interpreted by Reading Centre graders different to those involved in the masked assessment of the individual imaging tests and they will review all imaging and clinical information for each patient. Uncertain cases by primary grading will be reviewed by a senior adjudication panel (directors of the three Reading Centres) who will provide the reference standard in these cases.

Two versions of the reference standard will be provided:

- RS1 based on all imaging modalities except OCTA
- RS2 based on all imaging modalities including OCTA

This will allow assessment of the potential effect of incorporation bias from inclusion of the primary index test (OCTA) in determining the reference standard. The primary analyses will be based on RS1.

Economic Evaluation

In addition to the clinical diagnostic accuracy study, a model based cost effectiveness analysis (CEA) will be carried out to estimate the costs and benefits of each approach. The model will compare different strategies for tests (i.e. different combination of tests, different rules for diagnosis or subsequent testing). The time horizon for the model will cover the diagnostic testing period only and the evaluation perspective will be from the NHS perspective. The model structure will be finalised throughout the course of the study but will likely take the form of a decision tree. The model results will be expressed an incremental cost effectiveness ratio (ICER). Specifically, this will be presented as a cost per true positive detected for each of the strategies.

Both deterministic sensitivity analysis (DSA) and probabilistic sensitivity analysis (PSA) will be carried out. The DSA will assess plausible variation for parameters (e.g. differences in number of resources or unit costs) and how this impacts conclusions. The PSA will assess joint impression around the parameters and how this impacts the model estimates. This will likely take the form of a Monte Carlo simulation to address uncertainty. This model will adhere to best practice for economic decision [26].

Data Management

Processes will be employed to ensure the accuracy of data included in the final report. Coding and validation will be agreed on by the study manager, statistician and programmer; and the study database will be signed off once the implementation of these has been assured.

Data will be entered onto the study database by staff involved in the study or delegated staff at sites. Data entries within the electronic database will be scrutinised for completeness and accuracy, with discrepancies checked against paper data forms. The data

For peer review only

capture system will conduct automatic range checks for specific data values to ensure high levels of data quality. Any queries raised will be registered using the integrated data query system in the study database, with the expectation to be resolved within 30 days.
Every attempt will be made to collect full imaging data on all study participants, and the proportion of missing data is expected to be minimal. Participants with indeterminate or missing results will not be included in the primary analysis.

Study Termination

The end of study will be 6 months after the last data capture. This will allow sufficient time for the completion of protocol procedures, data collection and data input. The Trials Office will notify the Research Ethics Committee (REC) that the study has ended within 90 days of completion. In the instance of terminating the study early, the Trials Office will inform the REC within 15 days of study termination. The REC and Trial Office will receive a summary of the clinical study report within 12 months of study completion.

Consent and Ethics

The study will be performed in accordance with the recommendations guiding physicians in biomedical research involving human subjects, adopted by the 18th World Medical Association General Assembly, Helsinki, Finland, 1964, amended by the 48th WMA General Assembly, Somerset West, Republic of South Africa, 1996
Favourable opinion has been provided by the Research Ethics Committee and the study has received approval by the UK Health Research Authority.
Study participants will be required to give informed consent and sign a consent form. Trial participation is entirely voluntary, and the consent may be withdrawn at any time without this affecting patients' care.

Dissemination

Study dataset dissemination

The final pseudonymised study dataset will be made available to members of the study management group so that publications describing the outcomes (Table 2) can be prepared. Following publication of findings, the fully anonymised study dataset will be made available to external researchers upon approval from the study management group, the study steering committee, and the Sponsor in line with best data sharing practices for clinical study datasets.

Dissemination of Results

Results of this study will be submitted for publication in high impact peer reviewed journals.

The study question is important and has the potential to change clinical practice. Should the study results support this, the change in practice is expected to be rapidly incorporated into guidelines including the National Institute of Health and Care Excellence (NICE) and the Scientific Advisory Committees in the Department of Health.

The findings will be presented and disseminated via national and international conferences. A copy of the monograph will be lodged with the NIHR Journals Library as a permanent and comprehensive record of the study. In consultation with the investigators

For peer review only

and relevant journal, a press release will be issued to the media upon publication of the results.

Results of the study will be shared with study participants, staff members at research sites and investigators of other studies related to any of the imaging techniques investigated in ATHENA. Outreach to other key stakeholders (study networks, health advocates) involved in related studies is planned.

Acknowledgement and Contributors

MN collected methodological analysis plans and drafted the manuscript. JD, YT, SS, PJP, PAK, AK, LV, AKD and RG provided intellectual input in study methodology and design, contributed to drafting and critical review of the manuscript. ARK and WM are responsible for the planning of the statistical analysis. RA, GS, EM, PB, UC, NW. SM and TP provided intellectual input in study methodology and statistical analysis planning and critical review of the manuscript. KB conceived the study, initiated the project, provided intellectual input in study methodology and design and critical review of the manuscript.

The research team expresses its gratitude to the Study Oversight Committees for their valuable expert advice, steer, and support throughout the entire duration of the study.

Study Steering Committee: Rupert Bourne (Chair, Cambridge University Hospital and Anglia Ruskin University), Catey Bunce (The Royal Marsden NHS Foundation Trust), Geraldine Hoad (the Macular Society).

Data Monitoring Committee: Yit Yang (Chair, The Royal Wolverhampton NHS Trust), Irene Stratton (Gloucestershire Hospitals NHS Foundation Trust), Tomas Cudrnak (University Hospitals Plymouth NHS Foundation Trust).

We would also wish to acknowledge the valuable contribution to study pathway development and delivery of the Moorfields Ophthalmic Reading Centre team: Khadra Adan, Rosana Lima, Georgina Wignall, Alexandra Winston, Afreen Begum.

Funding Statement

The Sponsor for this study is Moorfields Eye Hospital NHS Foundation Trust who are also the Head Organisation (i.e. the contracting party with the funder).

Funding for the ATHENA study is provided by an award from the National Institute of Health Research, Health Technology Assessment Programme with Reference: NIHR131432.

Conflicts of Interest

None

For peer review only

References

1. Colijn JM, Buitendijk GHS, Prokofyeva E, al. e. Prevalence of Age-Related Macular Degeneration in Europe: The Past and the Future. Ophthalmology. 2017; 124(12): 1753-1763.

2. Jonas JB. Global prevalence of age-related macular degeneration.. Lancet Glob Health. 2014; 2(2): 65–66.

3. Wong WL, X S, Li X, Cheung CM, Klein R, Cheng CY, et al. Global prevalence of age-related macular degeneration and disease burden projection for 2020 and 2040: a systematic review and meta-analysis.. Lancet Glob Health. 2014; 2(2): 106–116.

4. Deng Y, Qiao L, Du M, Qu C, Wan L, Li J, et al. Age-related macular degeneration: Epidemiology, genetics, pathophysiology, diagnosis, and targeted therapy. Genes Dis. 2022; 9(1): 62–79.

5. Bird AC, Bressler NM, Bressler SB, Chisholm IH, Coscas G, Davis MD, et al. An international classification and grading system for age-related maculopathy and age-related macular degeneration. The International ARM Epidemiological Study Group. Surv Ophthalmol. 1995; 39(5): 367-74.

6. Bae K, Noh SR, Kang SW, Kim ES, Yu SY. Angiographic Subtypes of Neovascular Age-related Macular Degeneration in Korean: A New Diagnostic Challenge. Sci Rep. 2019; 9(1): 9701.

7. Farecki ML, Gutfleisch M, Faatz H, Rothaus K, Heimes B, Spital G, et al. Characteristics of type 1 and 2 CNV in exudative AMD in OCT-Angiography. Graefes Arch Clin Exp Ophthalmol. 2017; 255(5): 913-921.

8. Wong T, Chakravarthy U, Klein R, Mitchell P, Zlateva G, Buggage R, et al. The natural history and prognosis of neovascular age-related macular degeneration: a systematic review of the literature and meta-analysis. Ophthalmology. 2008; 115(1): 116-26.

9. Gess AJ, Fung AE, Rodriguez JG. Imaging in Neovascular Age-Related Macular Degeneration. Semin Ophthalmol. 2011; 26(3): 225-33.

10. Coleman HR, Chan CC, Ferris FL, Ferris 3rd CEY. Age-related macular degeneration. Lancet. 2008; 372(9652): 1835-45.

11. Tan CS NWCJTNLT. EVEREST study report 2: imaging and grading protocol, and baseline characteristics of a randomised controlled trial of polypoidal choroidal vasculopathy. Br J Ophthalmol. 2015; 99(1): 624–628.

12. Koh AH CLCSea. Expert PCV Panel Polypoidal choroidal vasculopathy: evidence-based guidelines for clinical diagnosis and treatment. Retina. 2013; 33(1): 686–716.

13. Fukuyama H IHATIHINGF. Indocyanine green dye filling time for polypoidal lesions in polypoidal choroidal vasculopathy affects the visibility of the lesions on OCT angiography. Ophthalmol Retina. 2018; 2(1): 803–807.

For peer review only

14. Liu R LJLZea. Distinguishing polypoidal choroidal vasculopathy from typical neovascular age-related macular degeneration based on spectral domain optical coherence tomography. Retina. 2016; 36(1): 778–786.

15. Faes L, Bodmer N, Locher S, Keane PA, Balaskas K, Bachmann LM, et al. Test performance of optical coherence tomography angiography in detecting retinal diseases: a systematic review and meta-analysis. Eye (Lond). 2019; 33(8): 1327-1338.

16. Cicinelli MV, Cavalleri M, Consorte A, Rabiolo A, Sacconi R, Bandello F, et al. Swept-source and spectral domain optical coherence tomography angiography versus dye angiography in the measurement of type 1 neovascularization. Retina. 2020; 40(3): 499-506.

17. Moult E, Choi W, Waheed N, Adhi M, Lee B, Lu C, et al. Ultrahigh-speed swept-source OCT angiography in exudative AMD. Ophthalmic Surg Lasers Imaging Retina. 2014; 45(6):496-505(45(6):496-505): 45(6):496-505.

18. Deng Y, Qiao L, Du M, Qu C, Wan L, Li J, et al. Quantitative optical coherence tomography angiography of choroidal neovascularization in age-related macular degeneration. Ophthalmology. 2014; 121(7): 1435–1444.

19. Cheung CMG, Yanagi Y, Akiba M, Tan A, Mathur R, Chan CM, et al. Improved detection and diagnosis of polypoidal choroidal vasculopathy using a combination of optical coherence tomography and optical coherence tomography angiography.. Retina. 2019; 39(9): 9.

20. Wolff B, De Bats F, Tick S, Cornut P, Souied É, Cohen S. Update from France Macula Federation: Diagnosis of wet AMD. J Fr Ophtalmol. 2018; 41(9): 857-861.

21. Faridi A, Jia Y, Gao SS, Huang D, Bhavsar K, Wilson DJ, et al. Sensitivity and Specificity of OCT Angiography to Detect Choroidal Neovascularization. Ophthalmol Retina. 2017; 1(4): 294-303.

22. Gong J, Yu S, Gong Y, Wang F, Sun X. The Diagnostic Accuracy of Optical Coherence Tomography Angiography for Neovascular Age-Related Macular Degeneration: A Comparison with Fundus Fluorescein Angiography. J Ophthalmol. 2016.

23. Gualino V, Tadayoni R, Cohen SY, Erginay A, Fajnkuchen F, Haouchine B, et al. OPTICAL COHERENCE TOMOGRAPHY, FLUORESCEIN ANGIOGRAPHY, AND DIAGNOSIS OF CHOROIDAL NEOVASCULARIZATION IN AGE-RELATED MACULAR DEGENERATION. Retina. 2019; 39(9): 1664-1671.

24. K. G. Computing inter-rater reliability and its variance in the presence of high agreement. British Journal of Mathematical and Statistical Psychology. 2010; 60(1): 29-48.

25. De Raadt A WMBRKH. Educational and Psychological Measurement. Educ Psychol Meas. 2019; 79(3): 558-576.

26. Briggs AH, ea. Model Parameter Estimation and Uncertainty: A Report of the ISPOR-SMDM Modeling Good Research Practices Task Force-6. Value in Health. 2012; 15(6): 835-842.

For peer review only

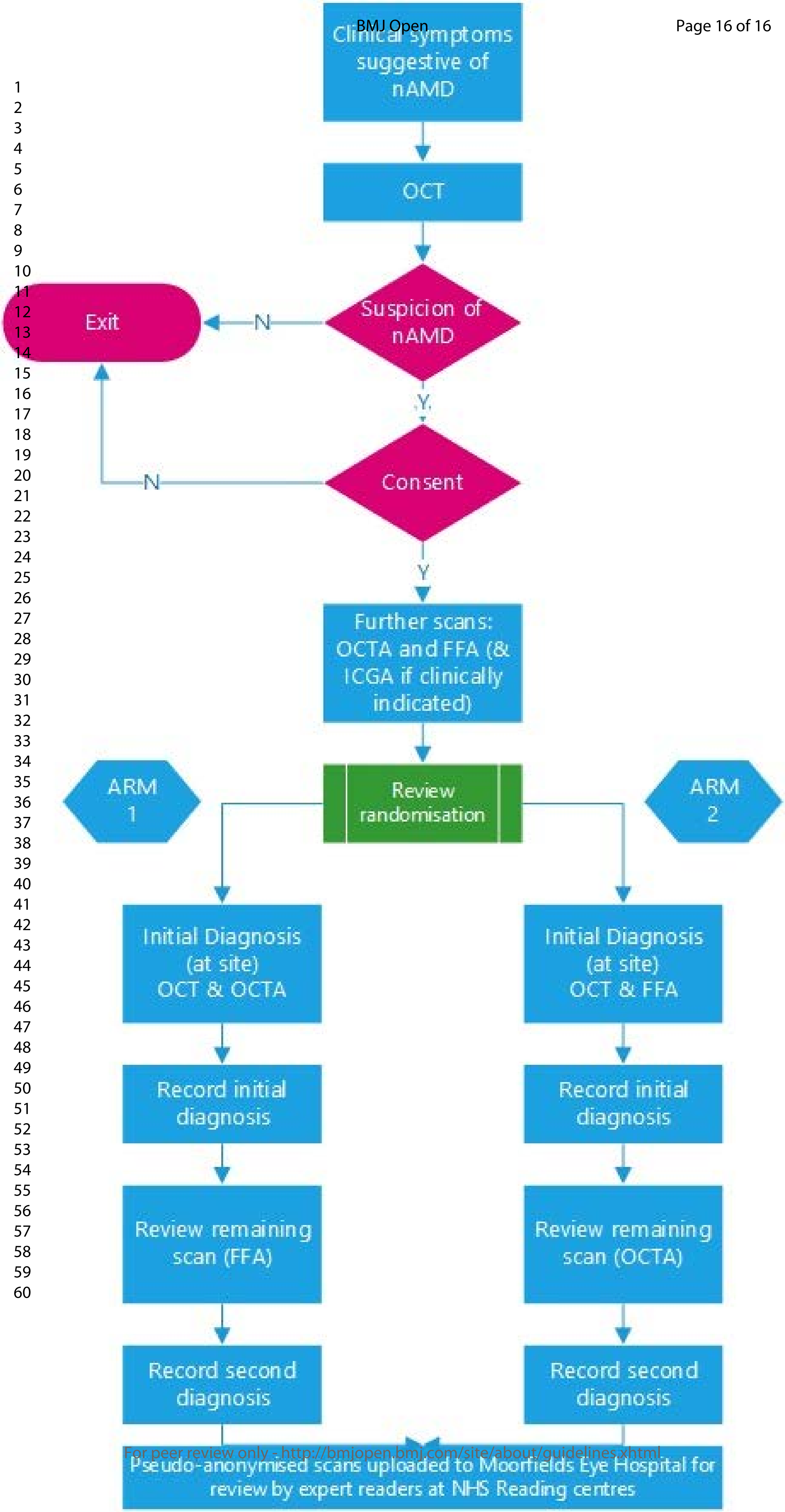
Clinical symptoms suggestive of nAMD
OCT
Suspicion of nAMD
N
Exit
Y
Consent
N
Y
Further scans: OCTA and FFA (& ICGA if clinically indicated)
Review randomisation
ARM 1
ARM 2
Initial Diagnosis (at site) OCT & OCTA
Initial Diagnosis (at site) OCT & FFA
Record initial diagnosis
Record initial diagnosis
Review remaining scan (FFA)
Review remaining scan (OCTA)
Record second diagnosis
Record second diagnosis
Pseudo-anonymised scans uploaded to Moorfields Eye Hospital for review by expert readers at NHS Reading centres

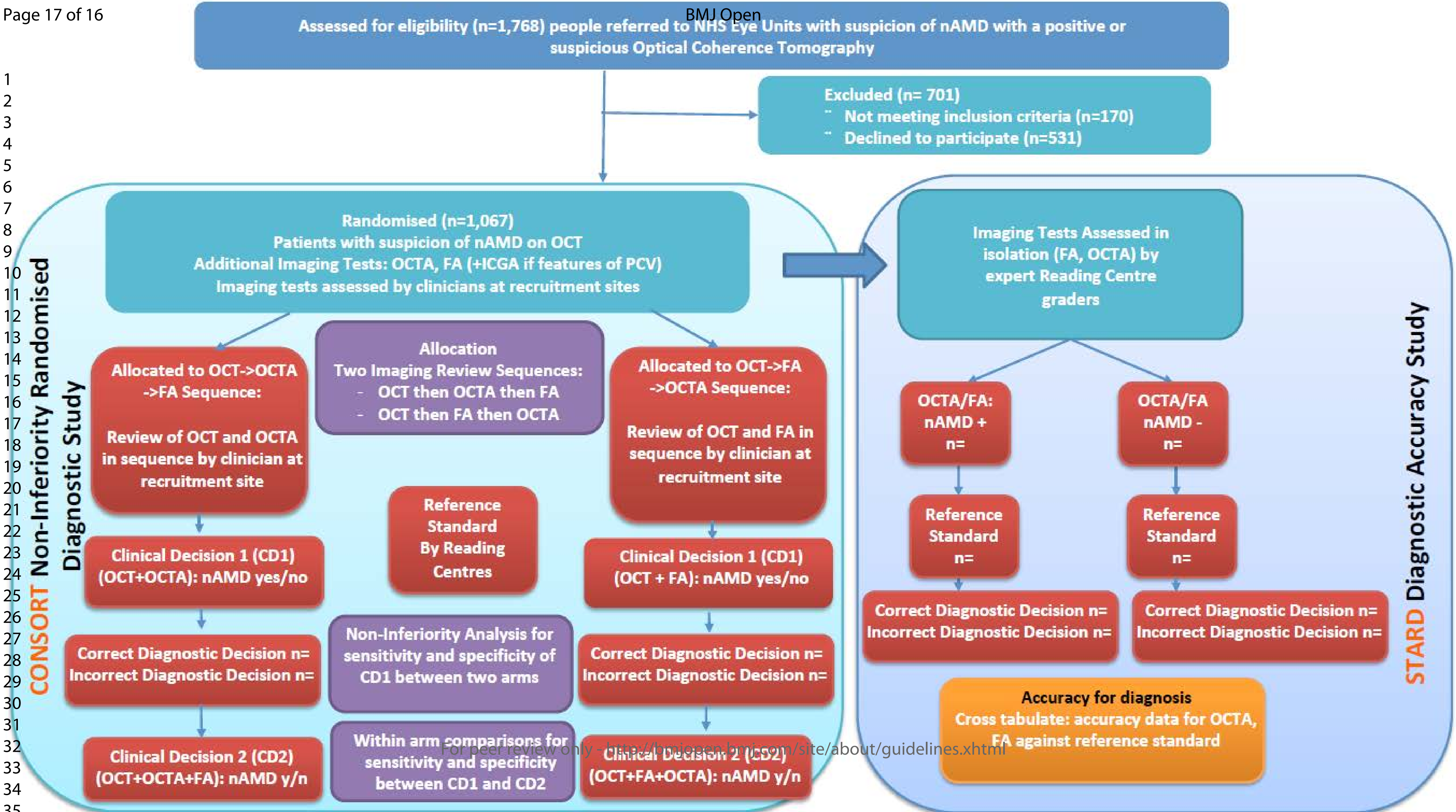
Assessed for eligibility (n=1,768) people referred to NHS Eye Units with suspicion of nAMD with a positive or suspicious Optical Coherence Tomography
Excluded (n= 701)
Not meeting inclusion criteria (n=170)
Declined to participate (n=531)
CONSORT Non-Inferiority Randomised Diagnostic Study
Randomised (n=1,067)
Patients with suspicion of nAMD on OCT
Additional Imaging Tests: OCTA, FA (+ICGA if features of PCV)
Imaging tests assessed by clinicians at recruitment sites
Allocated to OCT->OCTA ->FA Sequence:
Review of OCT and OCTA in sequence by clinician at recruitment site
Allocation
Two Imaging Review Sequences:
- OCT then OCTA then FA
- OCT then FA then OCTA
Allocated to OCT->FA ->OCTA Sequence:
Review of OCT and FA in sequence by clinician at recruitment site
Reference Standard By Reading Centres
Clinical Decision 1 (CD1) (OCT+OCTA): nAMD yes/no
Clinical Decision 1 (CD1) (OCT + FA): nAMD yes/no
Correct Diagnostic Decision n=
Incorrect Diagnostic Decision n=
Non-Inferiority Analysis for sensitivity and specificity of CD1 between two arms
Correct Diagnostic Decision n=
Incorrect Diagnostic Decision n=
Clinical Decision 2 (CD2) (OCT+OCTA+FA): nAMD y/n
Within arm comparisons for sensitivity and specificity between CD1 and CD2
Clinical Decision 2 (CD2) (OCT+FA+OCTA): nAMD y/n
STARD Diagnostic Accuracy Study
Imaging Tests Assessed in isolation (FA, OCTA) by expert Reading Centre graders
OCTA/FA: nAMD + n=
OCTA/FA nAMD - n=
Reference Standard n=
Reference Standard n=
Correct Diagnostic Decision n=
Incorrect Diagnostic Decision n=
Correct Diagnostic Decision n=
Incorrect Diagnostic Decision n=
Accuracy for diagnosis
Cross tabulate: accuracy data for OCTA, FA against reference standard

BMJ Open

Study Protocol: Optical Coherence Tomography Angiography for the detection of Neovascular Age-related Macular Degeneration: a Comprehensive Multi Centre Diagnostic Accuracy Study in the UK - the ATHENA study

Journal:	*BMJ Open*
Manuscript ID	bmjopen-2022-070857.R2
Article Type:	Protocol
Date Submitted by the Author:	25-May-2023
Complete List of Authors:	Niestrata, Magdalena; Moorfields Eye Hospital City Road Campus, Ophthalmology<br>Deeks, Jonathan; University of Birmingham, Public Health, Epidemiology and Biostatistics<br>Takwoingi, Yemisi; University of Birmingham, Public Health and Epidemiology<br>Sivaprasad, Sobha; Moorfields Eye Hospital City Road Campus<br>Patel, Praveen; Moorfields Eye Hospital NHS Foundation Trust, NIHR Biomedical Research Centre<br>Keane, Pearse; Moorfields Eye Hospital NHS Foundation Trust<br>Kernohan, Ashleigh; Newcastle University<br>Vale, Luke; Newcastle University<br>Denniston, Alastair; Queen Elizabeth Hospital Birmingham, UK<br>Gale, Richard; York Teaching Hospital NHS Foundation Trust<br>Khan, Adam R.; University of Birmingham<br>McKinnon, William; University of Birmingham<br>Agarwal, Ridhi; University of Birmingham, Test Evaluation Research Group, Institute of Applied Health Research,<br>de Salvo, Gabriella; Southampton University Hospitals NHS Trust<br>Minos, Evangelos; North West Anglia NHS Foundation Trust, Ophthalmology<br>Barbeiro, Paulo; BlueWorks - United Kingdom<br>Chakravarthy, Usha; Queen's University Belfast, Ophthalmology<br>Waheed, Nadia K; New England Eye Center<br>Madhusudhan, Savita; Liverpool University Hospitals NHS Foundation Trust<br>Peto, Tunde; Queen's University Belfast Faculty of Medicine Health and Life Sciences<br>Balaskas, Konstantinos; NIHR Moorfields Biomedical Research Centre, NHS Foundation Trust and UCL Institute of Ophthalmology
**Primary Subject Heading**:	Ophthalmology
Secondary Subject Heading:	Ophthalmology
Keywords:	Ophthalmology < SURGERY, OPHTHALMOLOGY, Medical retina < OPHTHALMOLOGY

SCHOLARONE™
Manuscripts

Study Protocol: Optical Coherence Tomography Angiography for the detection of Neovascular Age-related Macular Degeneration: a Comprehensive Multi Centre Diagnostic Accuracy Study in the UK - the ATHENA study

Magdalena Niestrata [1],

Jon Deeks [2],

Yemisi Takwoingi [2],

Sobha Sivaprasad [1],

Praveen J. Patel [1],

Pearse A. Keane [1],

Ashleigh Kernohan [3],

Luke Vale [3],

Alastair K. Denniston [4],

Richard Gale [5],

Adam R. Khan [6],

William McKinnon [6],

Ridhi Agarwal [2],

Gabriella de Salvo [7],

Evangelos Minos [8],

Paulo Barbeiro [9],

Usha Chakravarthy [10],

Nadia Waheed [11],

Savita Madhusudhan [12],

Tunde Peto [10],

Konstantinos Balaskas [1]

1. NIHR Biomedical Research Centre at Moorfields Eye Hospital NHS Foundation Trust and UCL Institute of Ophthalmology, London, UK

For peer review only

2. Institute of Applied Health Research, University of Birmingham, Birmingham, UK
3. Health Economics Group, Population Health Sciences Institute, Newcastle University, Newcastle, UK
4. University Hospitals Birmingham NHS Foundation Trust, Birmingham, UK
5. York Teaching Hospital NHS Foundation Trust, York, UK
6. Birmingham Clinical Trials Unit, University of Birmingham, Birmingham, UK
7. Southampton University Hospital NHS Foundation Trust, Southampton, UK
8. North West Anglia NHS Foundation Trust, Hinchingbrooke and Peterborough, UK
9. Blueworks Medical Expert Diagnosis, Lisbon, Portugal
10. Queen's University Belfast, Belfast, UK
11. New England Eye Centre, Boston, USA
12. Liverpool University Hospitals NHS Foundation Trust, Liverpool, UK

Corresponding author: Konstantinos Balaskas, Moorfields Reading Centre and Clinical AI Lab, Moorfields Eye Hospital NHS Foundation Trust, 162 City Rd, London EC1V 2PD, k.balaskas@nhs.net

Wordcount: 3692

MeSH terms:

- Diagnostic Techniques, Ophthalmological
- Humans
- Reproducibility of Results
- Sensitivity and Specificity
- Tomography, Optical Coherence / methods
- Wet Macular Degeneration / diagnosis

For peer review only

Abstract

Introduction

The diagnosis of neovascular Age-related Macular Degeneration (nAMD), the leading cause of visual impairment in the developed world, relies on the interpretation of various imaging tests of the retina. These include invasive angiographic methods, such as Fundus Fluorescein Angiography (FFA) and, on occasion, Indocyanine-Green Angiography (ICGA). Newer, non-invasive imaging modalities, predominately Optical Coherence Tomography (OCT) and Optical Coherence Tomography Angiography (OCTA), have drastically transformed the diagnostic approach to nAMD. The aim of this study is to undertake a comprehensive diagnostic accuracy assessment of the various imaging modalities used in clinical practice for the diagnosis of nAMD (OCT, OCTA, FFA and, when a variant of nAMD called Polypoidal Choroidal Vasculopathy is suspected, ICGA) both alone and in various combinations.

Methods and analysis

This is a non-inferiority, prospective, randomised diagnostic accuracy study of 1067 Participants. Participants are patients with clinical features consistent with nAMD who present to a National Health Service (NHS) secondary care ophthalmology unit in the UK. Patients will undergo OCT as per standard practice and those with suspicious features of nAMD on OCT will be approached for participation in the study. Patients who agree to take part will also undergo both OCTA and FFA (and ICGA if indicated). Interpretation of the imaging tests will be undertaken by clinicians at recruitment sites. A randomised design was selected to avoid bias from consecutive review of all imaging tests by the same clinician. The primary outcome of the study will be the difference in sensitivity and specificity between OCT+OCTA and OCT+FFA (+/- ICGA) for nAMD detection as interpreted by clinicians at recruitment sites.

Ethics and dissemination

The study has been approved by the South Central - Oxford B Research Ethics Committee with reference number 21/SC/0412.

Dissemination of study results will involve peer-review publications, presentations at major national and international scientific conferences.

Strengths and limitations

Strengths

- Large multicentre comprehensive diagnostic accuracy study in the uk of all imaging modalities in common clinical use for nAMD diagnosis.
- The study design maximises its applicability to NHS clinical practice because test interpretation is made by clinicians at recruitment sites and it has an embedded economic evaluation.
- All imaging tests are transferred, randomly and after pseudonymisation, to a laboratory environment for unbiased review and determination of diagnostic accuracy of different imaging tests in isolation.

Limitations

- The study design is complex because randomisation for sequence of image review is required to prevent bias from consecutive interpretation of all imaging tests for each case by the same clinician at recruitment sites.
- The eligibility criterion of presence of nAMD suspicious features on OCT permits the assessment of Positive Predictive Value only for OCT but this study design ensures the feasibility of the recruitment plan.

Background and Study Aims

Age-related macular degeneration (AMD) is the most frequent cause of severe visual impairment and blindness in elderly populations in the developed world including the UK, Europe, the United States and Australia. With age being the strongest risk factor for the

For peer review only

development of AMD, the numbers of affected patients are steadily increasing in view of the aging population. [1] Consequently, the incidence of visual impairment and blindness caused by AMD has significantly increased from 1990 to 2010. [2] Worldwide, the number of patients affected by AMD is estimated to increase to 288 million in 2040. [3] [4]

AMD is categorised into non-neovascular or dry and neovascular or wet, based on the absence or presence of macular neovascularisation (MNV) [5] Neovascular AMD (nAMD) is characterised by fluid leakage or bleeding from new permeable vessels originating from the choroidal or deep retinal capillary network. MNV can be subclassified into type 1, otherwise known as occult, type 2, or classic and type 3 or retinal angiomatous proliferation (RAP), depending on the location of the MNV - beneath the retinal pigment epithelium (RPE), within the subretinal space and intraretinally, respectively. [6] [7]

In nAMD regardless of MNV subtype, treatment with anti-vascular endothelial growth factor (VEGF) therapy has been shown to be effective. In those cases, early diagnosis and treatment is associated with improved visual prognosis and, improved patient quality of life and reduced morbidity. [8]

The diagnosis relies on imaging techniques visualising the retina and choroidal circulation. Optical Coherence Tomography (OCT), which has revolutionised the diagnosis and management of numerous retinal pathologies, features of nAMD disease activity including subretinal and intraretinal fluid, however, cannot directly detect a CNV. Until recently, FFA has been the gold-standard for the diagnosis of nAMD as it allows for visualisation of leakage from MNV. [4]

Nonetheless, this procedure is associated with the risk of complications, such as discoloration of urine, nausea and vomiting, and carries an extremely low risk of anaphylactic reaction. It is also contraindicated in kidney failure, which is not uncommon in elderly patients with systemic comorbidities who are investigated for nAMD. [9] The procedure is invasive, requiring intravenous cannulation and unpleasant for patients. In addition, the acquisition of images from FFA is time and labour-consuming, leading to significant difficulty due to limited resources and increasing numbers of patients requiring diagnostics. [10] Finally, occasionally FFA alone is not sufficient and additional imaging, such as Indocyanine-Green Angiography (ICGA) is required, particularly when Polypoidal Choroidal Vasculopathy (PCV), a variant of nAMD, is suspected.

ICGA has long been considered as the standard for PCV diagnosis and is generally considered as essential to make a definitive diagnosis of PCV. [11] Other imaging modalities have been assessed for the detection of PCV. [12] Fukuyama et al evaluated 62 polypoidal lesions detected on ICGA and 79% were detected on OCTA. [13] In a study which evaluated the SD-OCT findings of 188 eyes with PCV or neovascular AMD, results suggested that SD-OCT had a 89% sensitivity and 85% specificity in identifying PCV when 2 of the 3 signs were present: PED, double-layer sign, and thumb-like polyp. [14]

The advent of new non-invasive imaging techniques based on OCT, such as Optical Coherence Tomography Angiography (OCTA) has enabled a rapid direct visualisation of MNV without the need for intravenous contrast and without the side effects associated with FFA.

For peer review only

Faes and colleagues conducted a systematic review and meta-analysis of the performance of OCTA in detecting retinal disease. Over 1600 research articles involving OCTA have been published but only 17 studies were diagnostic accuracy studies. None of the studies were prospective and they provided scarce data on patient selection, data collection and analysis, masking, descriptions of the OCTA results and the reference standard. Out of the 17 studies, only 5 small studies evaluated detection of nAMD. These reported a wide range of sensitivity (0.50 to 1.00) and specificity (0.68 to 1.00), reinforcing the need for a well-designed diagnostic accuracy study to inform NHS decision-making on the role of OCTA. [15]

Cicinelli and colleagues (2020) have shown the overall sensitivity of OCTA for detection of active CNV to be 80.7% [16] In addition, the OCTA images have been shown to be less affected by subretinal haemorrhage compared to FFA. [17] [18] In terms of PCV detection, Cheung and colleagues showed a combination of structural OCT and OCTA achieved 82.6% sensitivity and 100% specificity [19] As a result, RCOphth guidance and some countries, such as France, have already changed their guidelines advocating increasing use of OCTA instead of FFA for nAMD diagnosis [20] However, there are no prospective, statistically powered, diagnostic accuracy studies that could inform the optimal diagnostic imaging protocol for nAMD.

In addition to the clinical implications of the performance of OCTA compared with the other imaging strategies there may be significant resource implications associated with the different strategies. As such it is important to understand the relationship between any additional costs associated with certain imaging strategies and any benefits that they may provide. Alongside this diagnostic accuracy study, an economic decision modelling study will be used to assess the cost effectiveness of different strategies.

Due to the lack of high quality evidence, there is variability in clinical practice with respect to the optimal imaging protocol for nAMD diagnosis. The original clinical interest in OCTA as a potential replacement of FFA in the diagnosis of nAMD has been superseded by the understanding that both techniques have relative and, on occasion, complementary merits, yet their optimal use for nAMD diagnosis remains unclear [15] The aim of ATHENA is to address these clinically relevant research questions.

There are also significant resource implications associated with the different strategies. Therefore, to guide decision-making the value for money of the different imaging strategies will be estimated.

Methods

Study Design

The ATHENA study is a non-inferiority, prospective, randomised diagnostic accuracy study with embedded economic evaluation and an internal pilot to confirm feasibility of the recruitment plan. The study started on the 1st March 2022, with a planned end date of 31st December 2024.

For peer review only

Patient and Public Involvement

There was extensive patient and public involvement, as detailed in the supplementary elements.

Patient Population

Participants with a suspicion of nAMD in the first or second eye on OCT that present to an NHS secondary care ophthalmology unit in the UK will be recruited. They will be randomised to either OCT + FFA or OCT + OCTA study arms (**Figure 1**).

Study Procedures

In addition to OCT, each patient will undergo both OCTA and FFA. Patients with clinical or OCT appearance suggestive of PCV will also undergo ICGA at the same time as FFA (**Figure 1**).

In half the cases, selected randomly, an expert clinician at the recruitment site will review the OCT and then the OCTA and provide a clinical diagnosis (nAMD: yes/no). In the other half of the cases the site clinician will review the OCT and then the FFA and provide a clinical diagnosis (nAMD: yes/no) (CD1). (**Figure 1**) Subsequently, the same clinician will review the remaining imaging test in each case (FFA or OCTA) and will either confirm or change their initial clinical diagnosis (nAMD: yes/no) – recorded as clinical diagnosis 2 (CD2). (**Figure 1**) Comparison of the two clinical diagnoses within each arm will provide additional evidence concerning the added value of using a combination of both OCTA and FFA in patients with an OCT suggestive of nAMD.

Figure 1. Study design

Setting

Recruitment will take place in 20 hospital-based eye units across the UK. Of those 6 will be involved in the pilot study and all sites will be involved in the main study. The study coordinating centre (Trial Office) is the Birmingham Clinical Trials Unit (BCTU), based at the University of Birmingham.

Participants will be recruited amongst patients referred by their community optometrist, GP, or self-referred to secondary care eye casualty departments with a suspicion of nAMD and amongst patients already under the care of NHS eye units for an established diagnosis of nAMD in one eye who develop symptoms of it in the fellow eye. Using Eligibility criteria to define inclusion and exclusion criteria for potential patients **(Table 1).**

Eligibility Criteria

Table 1

Inclusion Criteria:	Exclusion Criteria:
Patients with suspicion of nAMD in the first or second eye who have:	Significant media opacities (cataract, vitreous opacities) that would not allow good quality fundus imaging;

For peer review only

Signed informed consent form;	Diabetic retinopathy of severity worse than mild non-proliferative stage and with any degree of diabetic maculopathy;
Ability to perform study specific procedures.	Other causes of choroidal neovascularisation (myopic, angioid streaks, inflammatory, retinal dystrophies, secondary to central serous chorioretinopathy, idiopathic);
	Inability to undergo dye-based imaging (FFA or ICGA) due to history of allergy.

Randomisation and Blinding

The purpose of randomisation in this study is to minimise bias in the comparative accuracy of OCT+OCTA and OCT+FFA (primary objective) that may arise from non-masked interpretation of the findings of OCTA and FFA by the same clinician. Randomisation will be provided by a secure online randomisation system at BCTU (http://www.trials.bham.ac.uk/athena), which will be available 24 hours a day, 7 days a week, apart from short periods of scheduled maintenance. The system will randomise each patient to one of the two study arms as described in study design (**Figure 1**).

A minimisation algorithm will be used within the online randomisation system to ensure balance in the treatment allocation over the following variables: the recruiting centre, whether the affected eye is the first or second eye and whether the case is suspicious of PCV. A 'random element' will be included in the minimisation algorithm, so that each patient has a probability of being randomised to the opposite study arm than they would otherwise be assigned to.

Sample size calculation

According to current literature, the sensitivity of OCT + OCTA and OCT + FFA for detection of nAMD is 92% [15] [21] [22] and 90% [23], respectively. Based on the above, 240 patients with nAMD will need to be recruited to ensure, with 90% certainty, that the upper limit of a one-sided 97.5% confidence interval (or equivalently a 95% two-sided confidence interval) will exclude a difference in favour of OCT+FFA of more than 10 percentage points (i.e. the non-inferiority margin). A non-inferiority margin of 10 percentage points was deemed appropriate in view of the expected advantages of OCTA (i.e. improved patient experience through the use of a quick non-invasive imaging technique) and an already established role in clinical practice.

Audit data from Moorfields Eye Hospital indicated a prevalence of 75% of confirmed nAMD in patients with a positive or suspicious OCT, based on which, 320 patients with suspected nAMD will be required. However, as the aim of the study is to compare specificity as well as sensitivity, using the same estimates as for sensitivity, it is anticipated that 960 patients will be required. Allowing for 10% withdrawal rate, missing data and inconclusive test results, we would aim to recruit a total of 1,067 patients.

Objectives - Outcome Measures and their Analysis

The primary analysis, against the objectives **(Table 2)**, will be based on a pragmatic approach to image interpretation for nAMD detection by clinicians at recruitment sites (comparison of OCT+OCTA versus OCT+FFA in patients with positive or suspicious OCT). This approach reflects real-life clinical practice and will allow a meaningful assessment of the diagnostic accuracy of the most commonly used confirmatory tests for nAMD detection in a clinical setting. This approach will enhance the potential for generalisability of study results in the NHS.

Figure 2: Methodological flowchart

All images (OCT, OCTA, FA and where available ICGA) will also be transferred to NetwORC UK Reading Centres Network using a secure web-based image management system. The accuracy of the individual imaging tests in isolation (OCT, OCTA, FA) will be assessed in a masked and random fashion by expert graders in the controlled environment of the Reading Centres. This will ensure the masked assessment of individual imaging tests independent of each other with the use of a sophisticated grading queue that ensures random allocation of tests and minimises bias. Grading of the images will be performed at the three Reading Centres by trained graders who have achieved a minimum of 90% agreement in the accreditation test, as per figure 2 above which highlights the unique methodology that combines an RCT (CONSORT diagram) with a Diagnostic Accuracy study (STARD diagram). The allocation of images will take into account the study design so that graders involved in grading of the reference standard will not be involved in grading the index tests. Dedicated image management software will be used for the purposes of aggregating imaging data from recruitment sites and developing grading queues for Reading Centre graders. The imaging tests will be presented in random order to the graders to avoid bias associated with grading images from the same patient in sequence. Graders will be masked to all clinical information as well as whether the imaging tests being reviewed originate from the same patient or not.

Objectives

Table 2

Primary Objective	Primary Outcome Measure

For peer review only

**The main aim of this study is to assess whether the sensitivity and specificity of OCTA combined with OCT is non-inferior to that of FFA combined with OCT, for the detection of neovascular age-related macular degeneration, in patients with clinical and OCT features suggestive of the diagnosis; as interpreted by clinicians.**		The primary outcome measure is the difference in sensitivity and specificity between OCTA combined with OCT and FFA combined with OCT. The unit of analysis is the patient (one 'trial' eye per patient). The sensitivity, specificity, PPV and NPV of the tests will be calculated. The diagnostic accuracy of OCT+OCTA and OCT+FFA will be compared to assess non-inferiority. The differences in sensitivity and specificity will be established and two-sided 95% confidence intervals calculated using the Wilson CI score method. If the lower boundary of the two-sided 95% confidence interval (CI) for the difference in sensitivity and specificity of OCT+OCTA relative to OCT+FFA is higher than −10 percentage points, the sensitivity of OCT+OCTA will be considered as non-inferior

eer review

Secondary Objectives	Secondary Outcome Measures
**1. To assess the diagnostic accuracy of OCTA alone and FFA alone as reviewed by Reading Centre expert graders for the detection of nAMD in patients with a positive or suspicious OCT.**	Sensitivity, specificity, PPV and NPV of OCTA and FFA in isolation will be assessed on the basis of masked review by expert graders in the Reading Centres; 95% confidence intervals will be calculated using the Wilson method.
**2. To assess the positive predictive value (PPV) of OCT for the detection of nAMD in all patients presenting with suspicion of nAMD.**	The PPV of OCT for nAMD detection and its 95% CI will be estimated. Since only patients with a positive or suspicious OCT will be included, the sensitivity, specificity and NPV of OCT alone cannot be assessed.
**3. To compare the diagnostic accuracy of the combination of OCT+FFA versus OCT+FFA+OCTA as interpreted by retinal experts within the 'OCT+FFA' arm of the study.**	In order to establish the added diagnostic value of using a third imaging technique for nAMD detection, OCT+FFA will be compared against OCT+FFA+OCTA, in terms of differences in sensitivity and specificity together with their 95% Cis.

For peer review only - http://bmjopen.bmj.com/site/about/guidelines.xhtml

**4. To compare the diagnostic accuracy of the combination of OCT+OCTA versus OCT+FFA+OCTA as interpreted by retinal experts for the detection of nAMD within the 'OCT+OCTA' arm of the study.**	In order to establish the added diagnostic value of using a third imaging technique for nAMD detection, OCT+OCTA will be compared against OCT+OCTA+FFA, in terms of differences in sensitivity and specificity together with their 95% Cis.
**5. For a subset of cases with OCT and clinical features suspicious of Polypoidal Choroidal Vasculopathy (PCV) that underwent Indocyanine-Green Angiogrpahy (ICGA), to assess diagnostic accuracy of OCTA, FFA, ICGA, alone and in combinations, for the detection of PCV.**	For a subset of cases with clinical and OCT features suspicious of PCV, the diagnostic accuracy of OCTA, FFA and ICGA alone and in combination will be assessed by calculating their sensitivity, specificity, PPV and NPV (including their 95% CIs).
**6. To assess intra- and inter-rater agreement in the detection of nAMD on OCTA and FFA as assessed by Reading Centre graders.**	For OCTA and FFA, intra- and inter-rater agreement will be determined using percentage agreement and Gwet's first-order agreement coefficient (Gwet's AC1). [24] [25]
**7. To develop and validate criteria for the OCTA-based diagnosis of nAMD.**	The framework for OCTA-based nAMD diagnostic criteria created through this study will be assessed against a reference standard from the Reading Centres using all available multi-modal imaging (OCT, OCTA, FFA and ICGA where available) enhanced with clinical vignette describing patient symptoms and follow-up clinical data.
**8. To estimate the incremental cost per true positive detected and incremental cost per correct diagnosis for nAMD through a model based cost-effectiveness analysis.**	A model-based cost-effectiveness analyses (CEA) will be conducted to estimate the incremental cost per true positive detected and incremental cost per correct diagnosis for nAMD.
**9. To assess the diagnostic accuracy of OCTA by lesion type (PCV, type 1-, type 2-, type 3- nAMD).**	Sensitivity, specificity, PPV and NPV (including their 95% CIs) of OCTA for detection on nAMD by lesion type (PCV, type 1-, type 2-, type 3- nAMD) will be computed based on assessment by expert graders in the Reading Centres.
**10. To report limitations of OCTA use and adverse events.**	Number and classification of adverse events

Reference Standard

To provide the reference standard we will use all available imaging information from multi-modal imaging (OCT, OCTA, FFA and ICGA where available) enhanced with clinical vignette describing patient symptoms and available follow-up clinical data. The images will be interpreted by Reading Centre graders different to those involved in the masked assessment of the individual imaging tests and they will review all imaging and clinical information for each patient. Uncertain cases by primary grading will be reviewed by a senior adjudication panel (directors of the three Reading Centres) who will provide the reference standard in these cases.

Two versions of the reference standard will be provided:

- RS1 based on all imaging modalities except OCTA
- RS2 based on all imaging modalities including OCTA

This will allow assessment of the potential effect of incorporation bias from inclusion of the primary index test (OCTA) in determining the reference standard. The primary analyses will be based on RS1.

Economic Evaluation

In addition to the clinical diagnostic accuracy study, a model based cost effectiveness analysis (CEA) will be carried out to estimate the costs and benefits of each approach. The model will compare different strategies for tests (i.e. different combination of tests, different rules for diagnosis or subsequent testing). The time horizon for the model will cover the diagnostic testing period only and the evaluation perspective will be from the NHS perspective. The model structure will be finalised throughout the course of the study but will likely take the form of a decision tree. The model results will be expressed an incremental cost effectiveness ratio (ICER). Specifically, this will be presented as a cost per true positive detected for each of the strategies.

Both deterministic sensitivity analysis (DSA) and probabilistic sensitivity analysis (PSA) will be carried out. The DSA will assess plausible variation for parameters (e.g. differences in number of resources or unit costs) and how this impacts conclusions. The PSA will assess joint impression around the parameters and how this impacts the model estimates. This will likely take the form of a Monte Carlo simulation to address uncertainty. This model will adhere to best practice for economic decision [26].

Data Management

Processes will be employed to ensure the accuracy of data included in the final report. Coding and validation will be agreed on by the study manager, statistician and programmer; and the study database will be signed off once the implementation of these has been assured.

Data will be entered onto the study database by staff involved in the study or delegated staff at sites. Data entries within the electronic database will be scrutinised for completeness and accuracy, with discrepancies checked against paper data forms. The data capture system will conduct automatic range checks for specific data values to ensure high

For peer review only

levels of data quality. Any queries raised will be registered using the integrated data query system in the study database, with the expectation to be resolved within 30 days.
Every attempt will be made to collect full imaging data on all study participants, and the proportion of missing data is expected to be minimal. Participants with indeterminate or missing results will not be included in the primary analysis.

Study Termination

The end of study will be 6 months after the last data capture. This will allow sufficient time for the completion of protocol procedures, data collection and data input. The Trials Office will notify the Research Ethics Committee (REC) that the study has ended within 90 days of completion. In the instance of terminating the study early, the Trials Office will inform the REC within 15 days of study termination. The REC and Trial Office will receive a summary of the clinical study report within 12 months of study completion.

Consent and Ethics

The study will be performed in accordance with the recommendations guiding physicians in biomedical research involving human subjects, adopted by the 18th World Medical Association General Assembly, Helsinki, Finland, 1964, amended by the 48th WMA General Assembly, Somerset West, Republic of South Africa, 1996
Favourable opinion has been provided by the Research Ethics Committee and the study has received approval by the UK Health Research Authority.
Study participants will be required to give informed consent and sign a consent form. Trial participation is entirely voluntary, and the consent may be withdrawn at any time without this affecting patients' care.

Dissemination

Study dataset dissemination

The final pseudonymised study dataset will be made available to members of the study management group so that publications describing the outcomes (Table 2) can be prepared. Following publication of findings, the fully anonymised study dataset will be made available to external researchers upon approval from the study management group, the study steering committee, and the Sponsor in line with best data sharing practices for clinical study datasets.

Dissemination of Results

Results of this study will be submitted for publication in high impact peer reviewed journals.

The study question is important and has the potential to change clinical practice. Should the study results support this, the change in practice is expected to be rapidly incorporated into guidelines including the National Institute of Health and Care Excellence (NICE) and the Scientific Advisory Committees in the Department of Health.

The findings will be presented and disseminated via national and international conferences. A copy of the monograph will be lodged with the NIHR Journals Library as a permanent and comprehensive record of the study. In consultation with the investigators and relevant journal, a press release will be issued to the media upon publication of the results.

For peer review only

Results of the study will be shared with study participants, staff members at research sites and investigators of other studies related to any of the imaging techniques investigated in ATHENA. Outreach to other key stakeholders (study networks, health advocates) involved in related studies is planned.

Contributors

MN collected methodological analysis plans and drafted the manuscript. JD, YT, SS, PJP, PAK, AK, LV, AKD and RG provided intellectual input in study methodology and design, contributed to drafting and critical review of the manuscript. ARK and WM are responsible for the planning of the statistical analysis. RA, GS, EM, PB, UC, NW. SM and TP provided intellectual input in study methodology and statistical analysis planning and critical review of the manuscript. KB conceived the study, initiated the project, provided intellectual input in study methodology and design and critical review of the manuscript.

Acknowledgements

The research team expresses its gratitude to the Study Oversight Committees for their valuable expert advice, steer, and support throughout the entire duration of the study.

Study Steering Committee: Rupert Bourne (Chair, Cambridge University Hospital and Anglia Ruskin University), Catey Bunce (The Royal Marsden NHS Foundation Trust), Geraldine Hoad (the Macular Society).

Data Monitoring Committee: Yit Yang (Chair, The Royal Wolverhampton NHS Trust), Irene Stratton (Gloucestershire Hospitals NHS Foundation Trust), Tomas Cudrnak (University Hospitals Plymouth NHS Foundation Trust).

We would also wish to acknowledge the valuable contribution to study pathway development and delivery of the Moorfields Ophthalmic Reading Centre team: Khadra Adan, Rosana Lima, Georgina Wignall, Alexandra Winston, Afreen Begum.

Funding Statement

The Sponsor for this study is Moorfields Eye Hospital NHS Foundation Trust who are also the Head Organisation (i.e. the contracting party with the funder).

Funding for the ATHENA study is provided by an award from the National Institute of Health Research, Health Technology Assessment Programme with Reference: NIHR131432.

Conflicts of Interest

None

For peer review only

References

1. Colijn JM, Buitendijk GHS, Prokofyeva E, al. e. Prevalence of Age-Related Macular Degeneration in Europe: The Past and the Future. Ophthalmology. 2017; 124(12): 1753-1763.

2. Jonas JB. Global prevalence of age-related macular degeneration.. Lancet Glob Health. 2014; 2(2): 65–66.

3. Wong WL, X S, Li X, Cheung CM, Klein R, Cheng CY, et al. Global prevalence of age-related macular degeneration and disease burden projection for 2020 and 2040: a systematic review and meta-analysis.. Lancet Glob Health. 2014; 2(2): 106–116.

4. Deng Y, Qiao L, Du M, Qu C, Wan L, Li J, et al. Age-related macular degeneration: Epidemiology, genetics, pathophysiology, diagnosis, and targeted therapy. Genes Dis. 2022; 9(1): 62–79.

5. Bird AC, Bressler NM, Bressler SB, Chisholm IH, Coscas G, Davis MD, et al. An international classification and grading system for age-related maculopathy and age-related macular degeneration. The International ARM Epidemiological Study Group. Surv Ophthalmol. 1995; 39(5): 367-74.

6. Bae K, Noh SR, Kang SW, Kim ES, Yu SY. Angiographic Subtypes of Neovascular Age-related Macular Degeneration in Korean: A New Diagnostic Challenge. Sci Rep. 2019; 9(1): 9701.

7. Farecki ML, Gutfleisch M, Faatz H, Rothaus K, Heimes B, Spital G, et al. Characteristics of type 1 and 2 CNV in exudative AMD in OCT-Angiography. Graefes Arch Clin Exp Ophthalmol. 2017; 255(5): 913-921.

8. Wong T, Chakravarthy U, Klein R, Mitchell P, Zlateva G, Buggage R, et al. The natural history and prognosis of neovascular age-related macular degeneration: a systematic review of the literature and meta-analysis. Ophthalmology. 2008; 115(1): 116-26.

9. Gess AJ, Fung AE, Rodriguez JG. Imaging in Neovascular Age-Related Macular Degeneration. Semin Ophthalmol. 2011; 26(3): 225-33.

10. Coleman HR, Chan CC, Ferris FL, Ferris 3rd CEY. Age-related macular degeneration. Lancet. 2008; 372(9652): 1835-45.

11. Tan CS NWCJTNLT. EVEREST study report 2: imaging and grading protocol, and baseline characteristics of a randomised controlled trial of polypoidal choroidal vasculopathy. Br J Ophthalmol. 2015; 99(1): 624–628.

12. Koh AH CLCSea. Expert PCV Panel Polypoidal choroidal vasculopathy: evidence-based guidelines for clinical diagnosis and treatment. Retina. 2013; 33(1): 686–716.

13. Fukuyama H IHATIHINGF. Indocyanine green dye filling time for polypoidal lesions in polypoidal choroidal vasculopathy affects the visibility of the lesions on OCT angiography. Ophthalmol Retina. 2018; 2(1): 803–807.

For peer review only

14. Liu R LJLZea. Distinguishing polypoidal choroidal vasculopathy from typical neovascular age-related macular degeneration based on spectral domain optical coherence tomography. Retina. 2016; 36(1): 778–786.

15. Faes L, Bodmer N, Locher S, Keane PA, Balaskas K, Bachmann LM, et al. Test performance of optical coherence tomography angiography in detecting retinal diseases: a systematic review and meta-analysis. Eye (Lond). 2019; 33(8): 1327-1338.

16. Cicinelli MV, Cavalleri M, Consorte A, Rabiolo A, Sacconi R, Bandello F, et al. Swept-source and spectral domain optical coherence tomography angiography versus dye angiography in the measurement of type 1 neovascularization. Retina. 2020; 40(3): 499-506.

17. Moult E, Choi W, Waheed N, Adhi M, Lee B, Lu C, et al. Ultrahigh-speed swept-source OCT angiography in exudative AMD. Ophthalmic Surg Lasers Imaging Retina. 2014; 45(6):496-505(45(6):496-505): 45(6):496-505.

18. Deng Y, Qiao L, Du M, Qu C, Wan L, Li J, et al. Quantitative optical coherence tomography angiography of choroidal neovascularization in age-related macular degeneration. Ophthalmology. 2014; 121(7): 1435–1444.

19. Cheung CMG, Yanagi Y, Akiba M, Tan A, Mathur R, Chan CM, et al. Improved detection and diagnosis of polypoidal choroidal vasculopathy using a combination of optical coherence tomography and optical coherence tomography angiography.. Retina. 2019; 39(9): 9.

20. Wolff B, De Bats F, Tick S, Cornut P, Souied É, Cohen S. Update from France Macula Federation: Diagnosis of wet AMD. J Fr Ophtalmol. 2018; 41(9): 857-861.

21. Faridi A, Jia Y, Gao SS, Huang D, Bhavsar K, Wilson DJ, et al. Sensitivity and Specificity of OCT Angiography to Detect Choroidal Neovascularization. Ophthalmol Retina. 2017; 1(4): 294-303.

22. Gong J, Yu S, Gong Y, Wang F, Sun X. The Diagnostic Accuracy of Optical Coherence Tomography Angiography for Neovascular Age-Related Macular Degeneration: A Comparison with Fundus Fluorescein Angiography. J Ophthalmol. 2016.

23. Gualino V, Tadayoni R, Cohen SY, Erginay A, Fajnkuchen F, Haouchine B, et al. OPTICAL COHERENCE TOMOGRAPHY, FLUORESCEIN ANGIOGRAPHY, AND DIAGNOSIS OF CHOROIDAL NEOVASCULARIZATION IN AGE-RELATED MACULAR DEGENERATION. Retina. 2019; 39(9): 1664-1671.

24. K. G. Computing inter-rater reliability and its variance in the presence of high agreement. British Journal of Mathematical and Statistical Psychology. 2010; 60(1): 29-48.

25. De Raadt A WMBRKH. Educational and Psychological Measurement. Educ Psychol Meas. 2019; 79(3): 558-576.

26. Briggs AH, ea. Model Parameter Estimation and Uncertainty: A Report of the ISPOR-SMDM Modeling Good Research Practices Task Force-6. Value in Health. 2012; 15(6): 835-842.

For peer review only

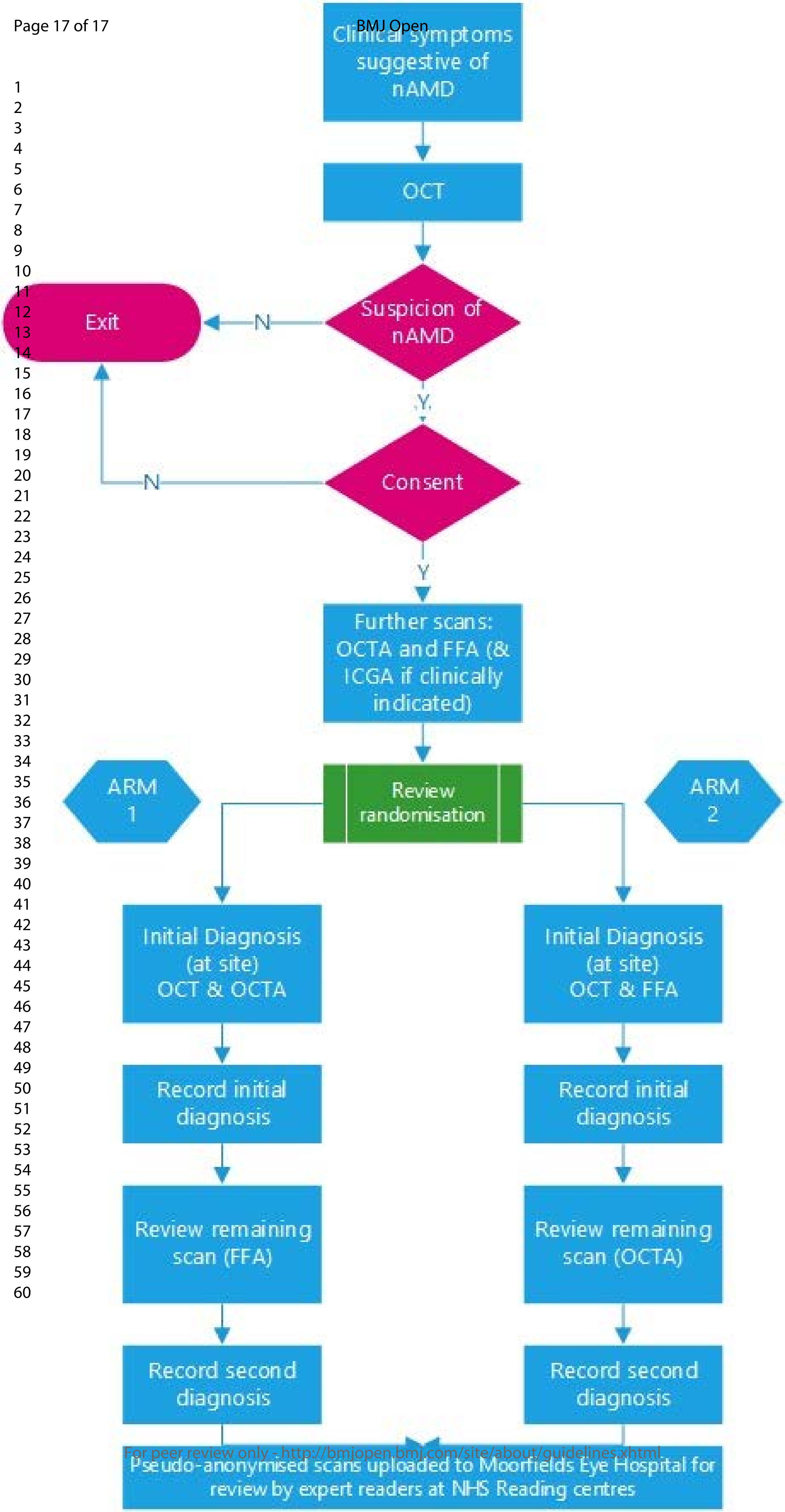
Clinical symptoms suggestive of nAMD
OCT
Suspicion of nAMD
N
Exit
Y
Consent
N
Y
Further scans: OCTA and FFA (& ICGA if clinically indicated)
ARM 1
Review randomisation
ARM 2
Initial Diagnosis (at site) OCT & OCTA
Initial Diagnosis (at site) OCT & FFA
Record initial diagnosis
Record initial diagnosis
Review remaining scan (FFA)
Review remaining scan (OCTA)
Record second diagnosis
Record second diagnosis
Pseudo-anonymised scans uploaded to Moorfields Eye Hospital for review by expert readers at NHS Reading centres

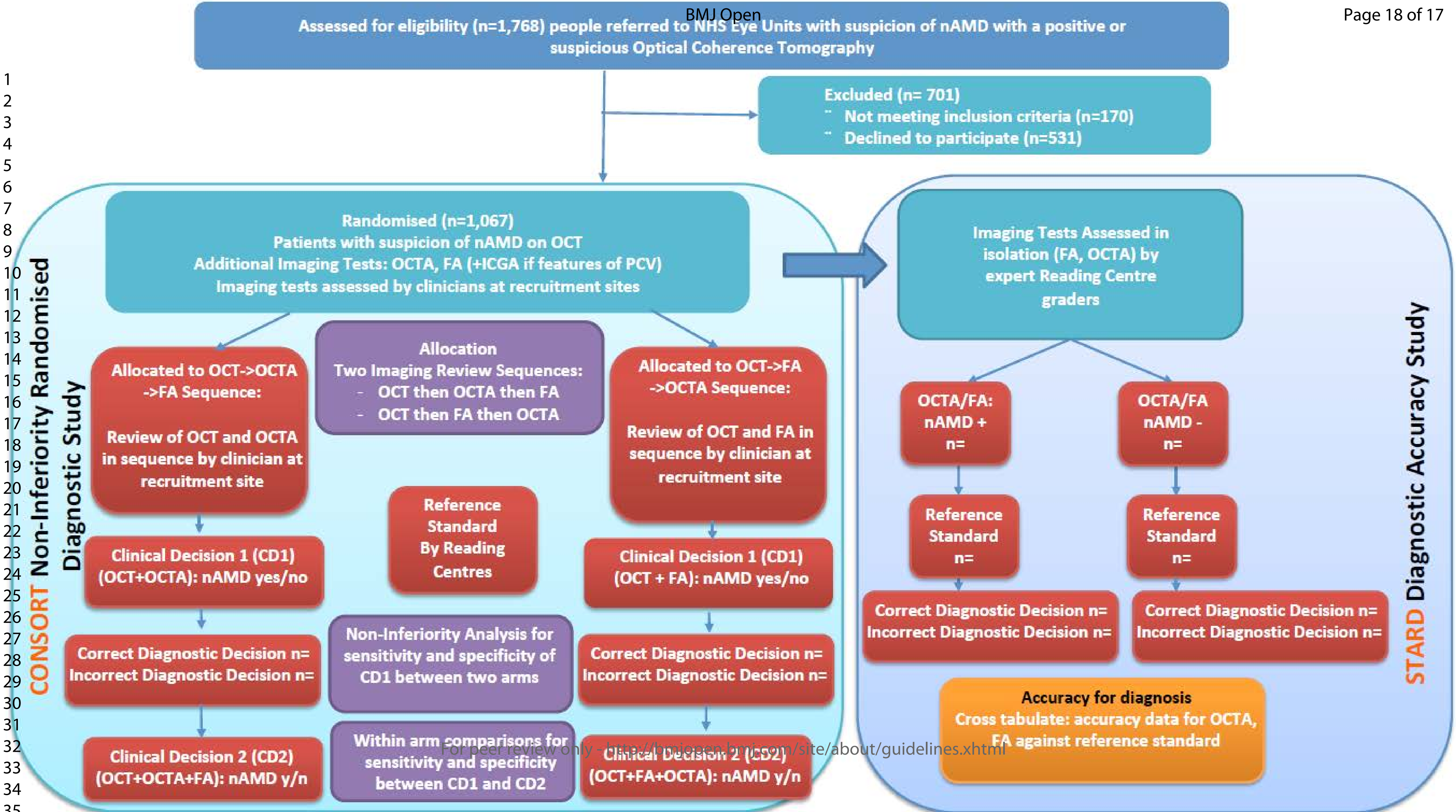
Assessed for eligibility (n=1,768) people referred to NHS Eye Units with suspicion of nAMD with a positive or suspicious Optical Coherence Tomography
Excluded (n= 701)
Not meeting inclusion criteria (n=170)
Declined to participate (n=531)
Randomised (n=1,067)
Patients with suspicion of nAMD on OCT
Additional Imaging Tests: OCTA, FA (+ICGA if features of PCV)
Imaging tests assessed by clinicians at recruitment sites
Allocated to OCT->OCTA ->FA Sequence:
Review of OCT and OCTA in sequence by clinician at recruitment site
Allocation
Two Imaging Review Sequences:
- OCT then OCTA then FA
- OCT then FA then OCTA
Allocated to OCT->FA ->OCTA Sequence:
Review of OCT and FA in sequence by clinician at recruitment site
Reference Standard By Reading Centres
Clinical Decision 1 (CD1) (OCT+OCTA): nAMD yes/no
Clinical Decision 1 (CD1) (OCT + FA): nAMD yes/no
Correct Diagnostic Decision n=
Incorrect Diagnostic Decision n=
Non-Inferiority Analysis for sensitivity and specificity of CD1 between two arms
Correct Diagnostic Decision n=
Incorrect Diagnostic Decision n=
Clinical Decision 2 (CD2) (OCT+OCTA+FA): nAMD y/n
Within arm comparisons for sensitivity and specificity between CD1 and CD2
Clinical Decision 2 (CD2) (OCT+FA+OCTA): nAMD y/n
CONSORT Non-Inferiority Randomised Diagnostic Study
Imaging Tests Assessed in isolation (FA, OCTA) by expert Reading Centre graders
OCTA/FA: nAMD + n=
OCTA/FA nAMD - n=
Reference Standard n=
Reference Standard n=
Correct Diagnostic Decision n=
Incorrect Diagnostic Decision n=
Correct Diagnostic Decision n=
Incorrect Diagnostic Decision n=
Accuracy for diagnosis
Cross tabulate: accuracy data for OCTA, FA against reference standard
STARD Diagnostic Accuracy Study
