# Supplementary material for: Study protocol: optical coherence tomography angiography for the detection of neovascular age-related macular degeneration: a comprehensive multicentre diagnostic accuracy study in the UK—the ATHENA study
Source: BMJ Open. 2024 May 31;14(5):e070857. doi: 10.1136/bmjopen-2022-070857 (PMC11149127; doi:10.1136/bmjopen-2022-070857)
Supplement: Reviewer comments [file bmjopen-2022-070857.reviewer_comments.pdf]

PEER REVIEW HISTORY

BMJ Open publishes all reviews undertaken for accepted manuscripts. Reviewers are asked to complete a checklist review form (**http://bmjopen.bmj.com/site/about/resources/checklist.pdf**) and are provided with free text boxes to elaborate on their assessment. These free text comments are reproduced below.

ARTICLE DETAILS

**TITLE (PROVISIONAL)**	Study Protocol: Optical Coherence Tomography Angiography for the detection of Neovascular Age-related Macular Degeneration: a Comprehensive Multi Centre Diagnostic Accuracy Study in the UK - the ATHENA study
**AUTHORS**	Niestrata, Magdalena; Deeks, Jonathan; Takwoingi, Yemisi; Sivaprasad, Sobha; Patel, Praveen; Keane, Pearse; Kernohan, Ashleigh; Vale, Luke; Denniston, Alastair; Gale, Richard; Khan, Adam R.; McKinnon, William; Agarwal, Ridhi; de Salvo, Gabriella; Minos, Evangelos; Barbeiro, Paulo; Chakravarthy, Usha; Waheed, Nadia K; Madhusudhan, Savita; Peto, Tunde; Balaskas, Konstantinos

This article was not externally reviewed at BMJ Open. Protocols that have been independently assessed prior to submission to BMJ Open are usually fast-tracked to publication on the grounds that further substantial changes will not be possible. This independent assessment will usually be external, independent review for both a substantial grant award from a non-commercial or government funder and ethics approval.
